# Supplementary material for: Two folds, many faces: The Magnaporthe oryzae MAX effector AVR-Pia targets novel rice HMA domain-containing proteins
Source: PLoS Pathog. 2026 Jul 13;22(7):e1014382. doi: 10.1371/journal.ppat.1014382 (PMC13395435; doi:10.1371/journal.ppat.1014382)
Supplement: S1 Fig — Comprehensive pairwise Y2H analysis using AVR-Pia, MAX58, Pwl2, and AVR-PikD as prey, and a library of HMA domains from OsH(I)PPs as bait [49]. The left panel shows yeast growth on selective media, while the right panel identifies the rice H(I)PP proteins from which the HMA domains were derived. Diploid yeast were spotted onto synthetic defined (SD) media to monitor growth (SD/-LW) or to assess protein-protein interactions (SD/-LWH). Pictures were taken after 7 days of incubation. AD, activating domain; BD, binding domain; *, Autoactive BD construct leading to yeast growth regardless of the presence or identity of the AD construct; +++, strong yeast growth; ++, normal yeast growth, + , weak yeast growth; No HMA, proteins for which no HMA domain was identified by sequence analysis or structural prediction; Wrong annotation, constructs based on misannotated genes, resulting in missing or truncated HMA domains, thereby compromising Y2H assay. (PDF) [file ppat.1014382.s001.pdf]

BD:HMA of OsH(I)PPs

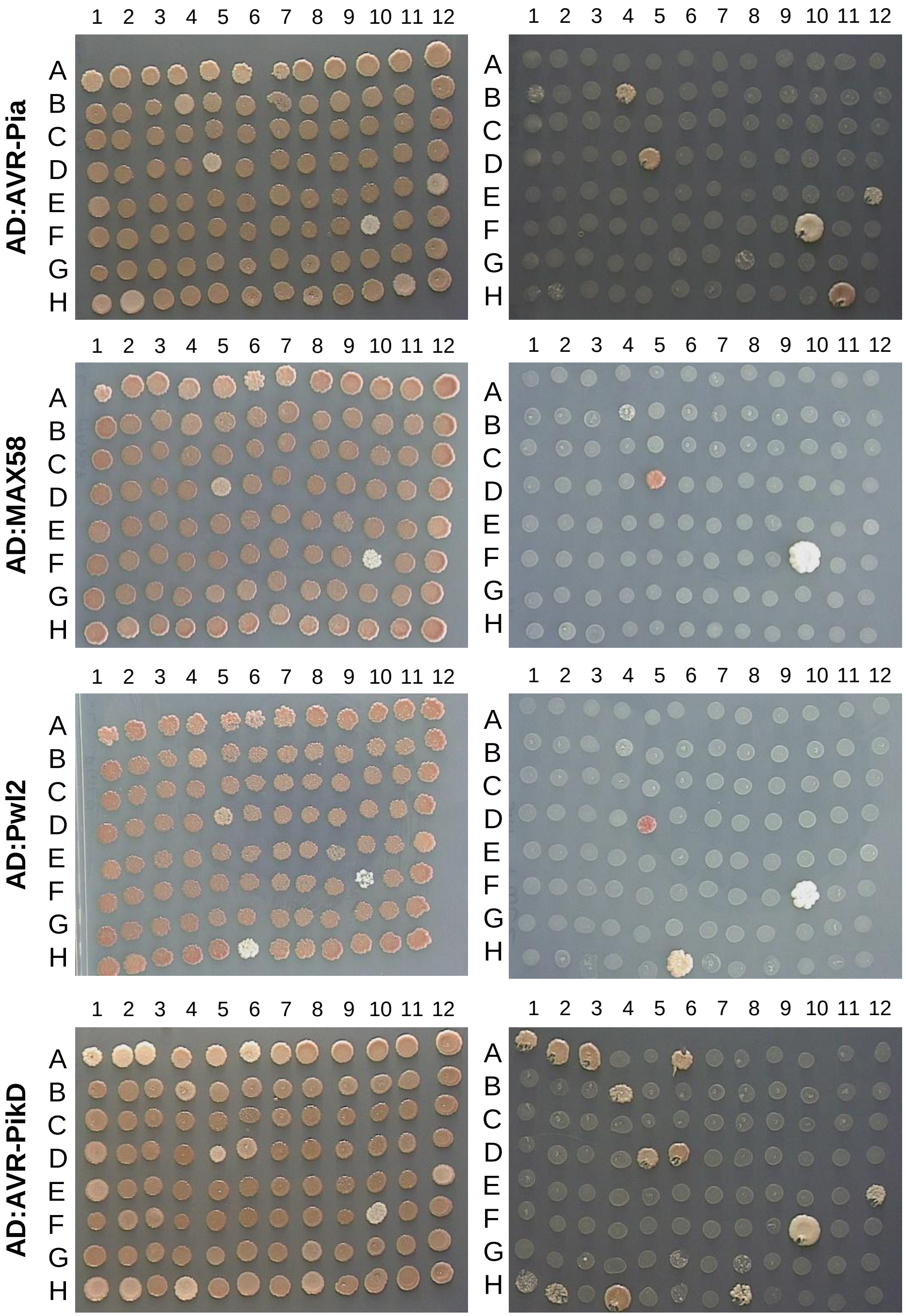

SD/-L-W   SD/-L-W-H

BD:HMA of OsH(I)PPs

	1	2	3	4	5	6	7	8	9	10	11	12
A	HIPP19	HIPP20	HIPP20	HPP04	HPP03	LOC_ Os04g39380	HPP02	LOC_ Os10g36200	LOC_ Os02g37320	LOC_ Os01g41200	HIPP38	HIPP47
B	LOC_ Os04g45130	HIPP54	LOC_ Os09g15860	Os09g0408550 *	LOC_ Os08g40130	HIPP29	HIPP49	HIPP04	HIPP15	HIPP50	HIPP22	LOC_ Os09g37730
C	HIPP02	HPP01	LOC_ Os09g15850	HIPP55	HIPP48	HIPP23	Os09g0408500	LOC_ Os01g52160	HIPP26	HIPP58	LOC_ Os05g45820	HIPP24
D	HIPP34	OJ1124_B05.17	LOC_ Os03g25610	LOC_ Os04g32830	LOC_ Os01g09660 *	HIPP18	HIPP06	HIPP09	HIPP32	HPP06	OsATX1	HIPP01
E	HIPP45	Os11g0629100	OsCCH	HIPP31	HIPP56	HIPP59	HIPP51	LOC_ Os06g30480	HIPP30	HPP07	HIPP25	HIPP41
F	LOC_ Os01g70240	HIPP40	LOC_ Os04g32860	HIPP10	HIPP17	OSJNBb0039L24.20	LOC_ Os04g42350	HIPP08	Os01g0249800	Os01g0595100 *	HIPP13	HIPP57
G	HIPP16	HIPP12	LOC_ Os02g48170	HIPP27	HIPP36	HIPP42	Os03g0829200	HIPP14	LOC_ Os02g37180	LOC_ Os04g040600	LOC_ Os01g61070	HIPP21
H	HPP05	HIPP39	HIPP07	LOC_ Os04g39370	LOC_ Os09g14240	HIPP43	HIPP28	HIPP46	HIPP33	HIPP05 (Pi21)	LOC_ Os03g02070	HIPP52

	1	2	3	4	5	6	7	8	9	10	11	12
A	HIPP19	HIPP20	HIPP20	HPP04	HPP03	LOC_ Os04g39380	HPP02	LOC_ Os10g36200	LOC_ Os02g37320	LOC_ Os01g41200	HIPP38	HIPP47
B	LOC_ Os04g45130	HIPP54	LOC_ Os09g15860	Os09g0408550 *	LOC_ Os08g40130	HIPP29	HIPP49	HIPP04	HIPP15	HIPP50	HIPP22	LOC_ Os09g37730
C	HIPP02	HPP01	LOC_ Os09g15850	HIPP55	HIPP48	HIPP23	Os09g0408500	LOC_ Os01g52160	HIPP26	HIPP58	LOC_ Os05g45820	HIPP24
D	HIPP34	OJ1124_B05.17	LOC_ Os03g25610	LOC_ Os04g32830	LOC_ Os01g09660 *	HIPP18	HIPP06	HIPP09	HIPP32	HPP06	OsATX1	HIPP01
E	HIPP45	Os11g0629100	OsCCH	HIPP31	HIPP56	HIPP59	HIPP51	LOC_ Os06g30480	HIPP30	HPP07	HIPP25	HIPP41
F	LOC_ Os01g70240	HIPP40	LOC_ Os04g32860	HIPP10	HIPP17	OSJNBb0039L24.20	LOC_ Os04g42350	HIPP08	Os01g0249800	Os01g0595100 *	HIPP13	HIPP57
G	HIPP16	HIPP12	LOC_ Os02g48170	HIPP27	HIPP36	HIPP42	Os03g0829200	HIPP14	LOC_ Os02g37180	LOC_ Os04g040600	LOC_ Os01g61070	HIPP21
H	HPP05	HIPP39	HIPP07	LOC_ Os04g39370	LOC_ Os09g14240	HIPP43	HIPP28	HIPP46	HIPP33	HIPP05 (Pi21)	LOC_ Os03g02070	HIPP52

	1	2	3	4	5	6	7	8	9	10	11	12
A	HIPP19	HIPP20	HIPP20	HPP04	HPP03	LOC_ Os04g39380	HPP02	LOC_ Os10g36200	LOC_ Os02g37320	LOC_ Os01g41200	HIPP38	HIPP47
B	LOC_ Os04g45130	HIPP54	LOC_ Os09g15860	Os09g0408550 *	LOC_ Os08g40130	HIPP29	HIPP49	HIPP04	HIPP15	HIPP50	HIPP22	LOC_ Os09g37730
C	HIPP02	HPP01	LOC_ Os09g15850	HIPP55	HIPP48	HIPP23	Os09g0408500	LOC_ Os01g52160	HIPP26	HIPP58	LOC_ Os05g45820	HIPP24
D	HIPP34	OJ1124_B05.17	LOC_ Os03g25610	LOC_ Os04g32830	LOC_ Os01g09660 *	HIPP18	HIPP06	HIPP09	HIPP32	HPP06	OsATX1	HIPP01
E	HIPP45	Os11g0629100	OsCCH	HIPP31	HIPP56	HIPP59	HIPP51	LOC_ Os06g30480	HIPP30	HPP07	HIPP25	HIPP41
F	LOC_ Os01g70240	HIPP40	LOC_ Os04g32860	HIPP10	HIPP17	OSJNBb0039L24.20	LOC_ Os04g42350	HIPP08	Os01g0249800	Os01g0595100 *	HIPP13	HIPP57
G	HIPP16	HIPP12	LOC_ Os02g48170	HIPP27	HIPP36	HIPP42	Os03g0829200	HIPP14	LOC_ Os02g37180	LOC_ Os04g040600	LOC_ Os01g61070	HIPP21
H	HPP05	HIPP39	HIPP07	LOC_ Os04g39370	LOC_ Os09g14240	HIPP43	HIPP28	HIPP46	HIPP33	HIPP05 (Pi21)	LOC_ Os03g02070	HIPP52

	1	2	3	4	5	6	7	8	9	10	11	12
A	HIPP19	HIPP20	HIPP20	HPP04	HPP03	LOC_ Os04g39380	HPP02	LOC_ Os10g36200	LOC_ Os02g37320	LOC_ Os01g41200	HIPP38	HIPP47
B	LOC_ Os04g45130	HIPP54	LOC_ Os09g15860	Os09g0408550 *	LOC_ Os08g40130	HIPP29	HIPP49	HIPP04	HIPP15	HIPP50	HIPP22	LOC_ Os09g37730
C	HIPP02	HPP01	LOC_ Os09g15850	HIPP55	HIPP48	HIPP23	Os09g0408500	LOC_ Os01g52160	HIPP26	HIPP58	LOC_ Os05g45820	HIPP24
D	HIPP34	OJ1124_B05.17	LOC_ Os03g25610	LOC_ Os04g32830	LOC_ Os01g09660 *	HIPP18	HIPP06	HIPP09	HIPP32	HPP06	OsATX1	HIPP01
E	HIPP45	Os11g0629100	OsCCH	HIPP31	HIPP56	HIPP59	HIPP51	LOC_ Os06g30480	HIPP30	HPP07	HIPP25	HIPP41
F	LOC_ Os01g70240	HIPP40	LOC_ Os04g32860	HIPP10	HIPP17	OSJNBb0039L24.20	LOC_ Os04g42350	HIPP08	Os01g0249800	Os01g0595100 *	HIPP13	HIPP57
G	HIPP16	HIPP12	LOC_ Os02g48170	HIPP27	HIPP36	HIPP42	Os03g0829200	HIPP14	LOC_ Os02g37180	LOC_ Os04g040600	LOC_ Os01g61070	HIPP21
H	HPP05	HIPP39	HIPP07	LOC_ Os04g39370	LOC_ Os09g14240	HIPP43	HIPP28	HIPP46	HIPP33	HIPP05 (Pi21)	LOC_ Os03g02070	HIPP52

Interaction

* Autoactive   +++   ++   +   No HMA   Wrong annotation
