# Supplementary figures and images for: Two folds, many faces: The Magnaporthe oryzae MAX effector AVR-Pia targets novel rice HMA domain-containing proteins
Source: PLoS Pathog. 2026 Jul 13;22(7):e1014382. doi: 10.1371/journal.ppat.1014382 (PMC13395435; doi:10.1371/journal.ppat.1014382)

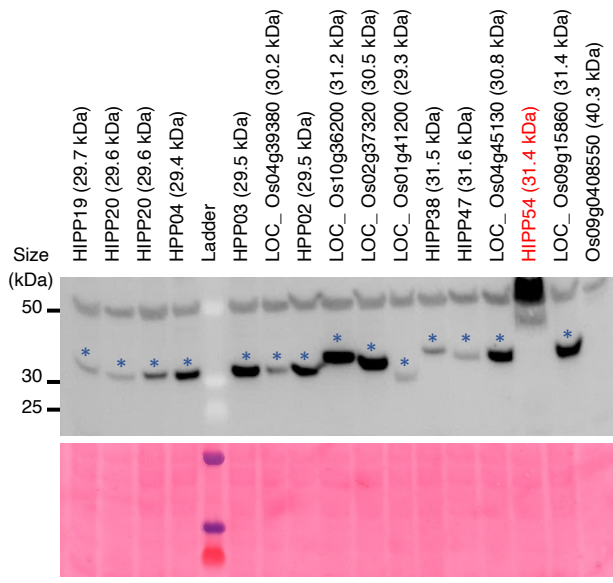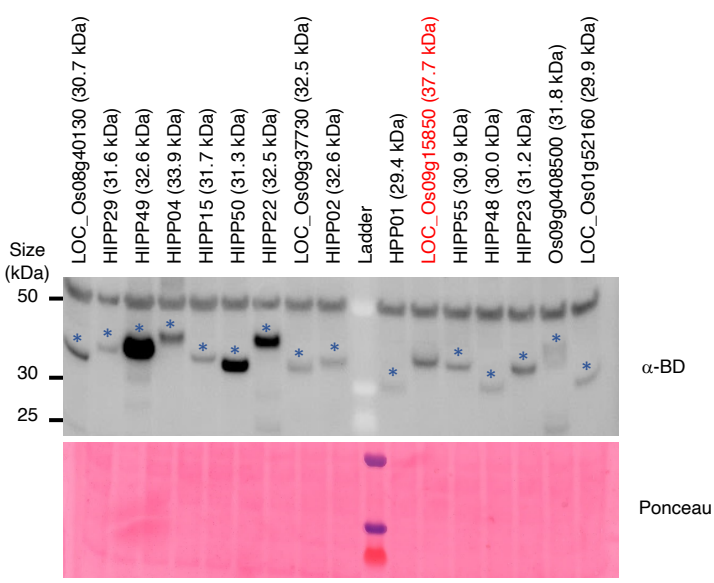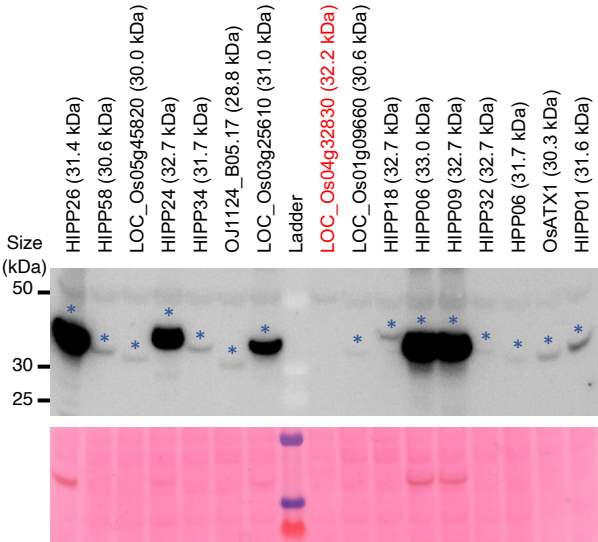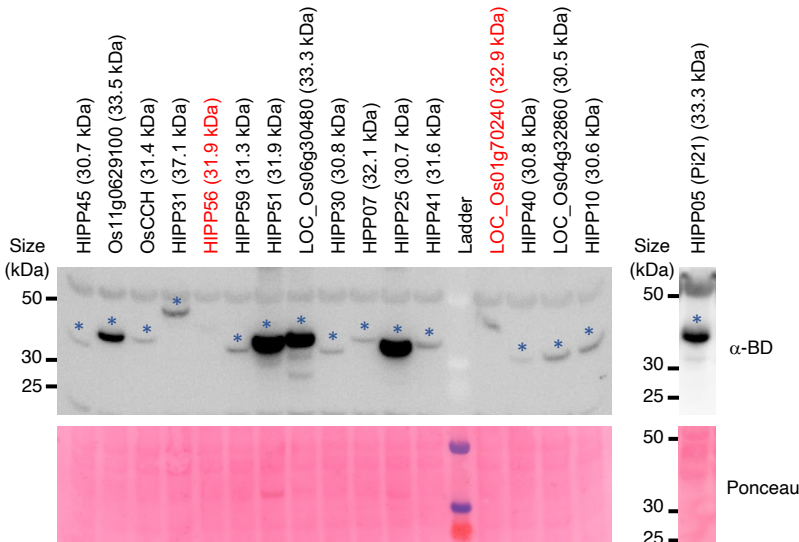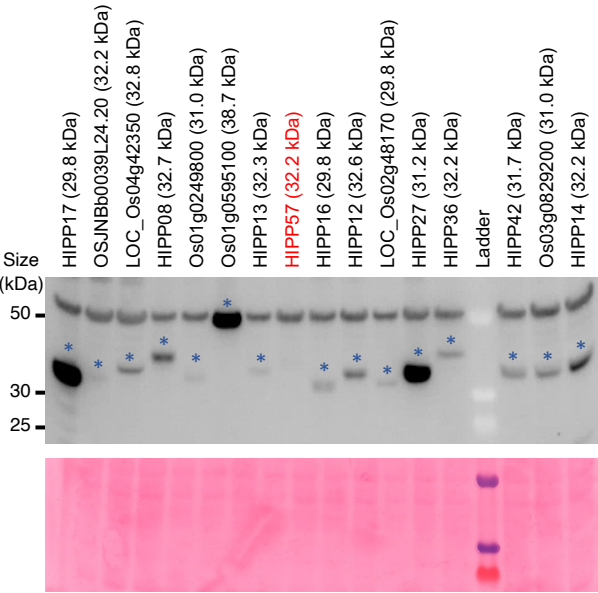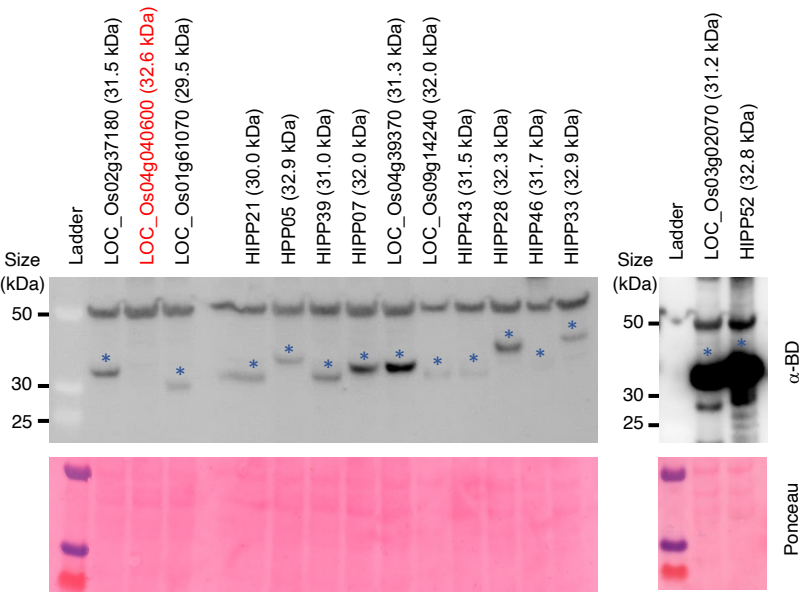

Supplement: S2 Fig — Total yeast protein extracts from haploid yeast were separated on a 10% NuPAGE Bis-Tris gel and analysed by immunoblot using an anti-BD antibody to detect Gal4-BD fusion proteins with HMA domains from rice H(I)PPs. Blue asterisks indicate the band corresponding to the expected construct, while non-expressed constructs are marked in red. Protein loading is indicated by Ponceau S staining. (PDF) [file ppat.1014382.s002.pdf]

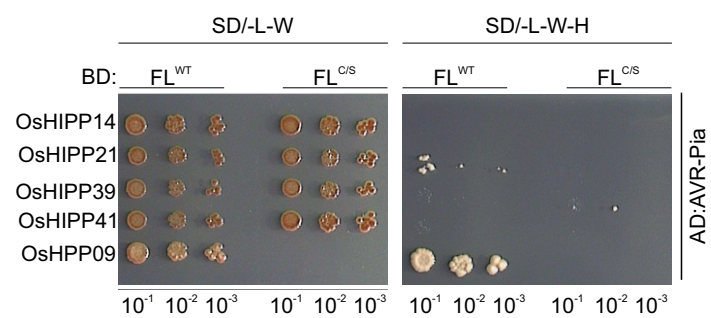

Supplement: S3 Fig — Y2H interaction analysis between AVR-Pia and either wildtype (WT) full-length (FL) OsHIPPs, including OsHIPP14, OsHIPP21, OsHIPP39 and OsHIPP41, or the corresponding FL OsHIPP isoprenylation mutants, in which the cysteine (C) residue was replaced by a non-prenylatable serine (S) residue. OsHPP09/AVR-Pia and was included as positive control. Serial dilutions of diploid yeast were spotted onto synthetic defined (SD) media to monitor growth (SD/-LW) or to assess protein-protein interactions (SD/-LWH). Photos were taken after 7 days of incubation. AD, activating domain; BD, binding domain. OsHIPPC/S mutants: OsHIPP14C187S, OsHIPP21C133S, OsHIPP39C190S, OsHIPP41C152S. (PDF) [file ppat.1014382.s003.pdf]

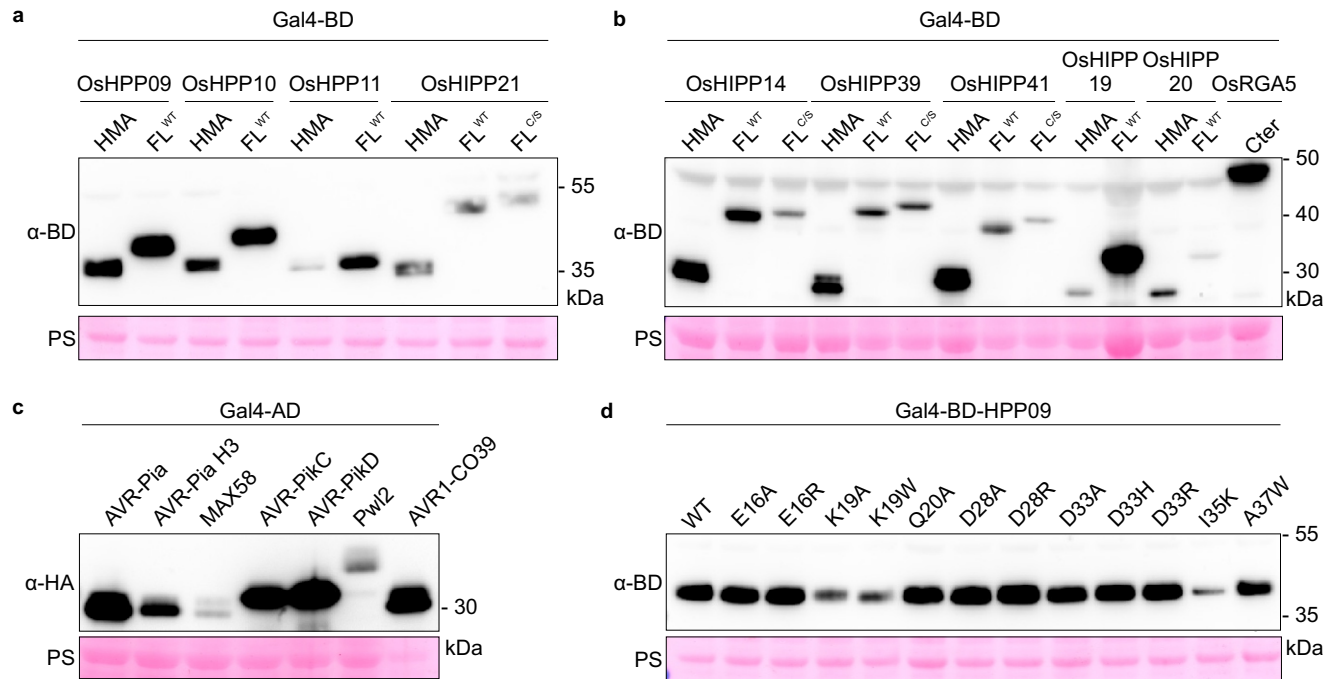

Supplement: S4 Fig — a Total yeast protein extracts from haploid yeast were separated on a 10 – 20% Tricine SDS-PAGE gel and analysed by immunoblot using an anti-BD antibody to detect Gal4-BD fusion proteins with either the heavy metal-associated (HMA) domain, full-length (FL) OsH(I)PPs and the OsHIPP21 isoprenylation mutant (C/S).Protein loading is indicated by Ponceau S staining (PS). b Total yeast protein extracts from haploid yeast were separated on a 10% NuPAGE Bis-Tris gel and analysed by immunoblot using an anti-BD antibody to detect Gal4-BD fusion proteins with either the HMA domain, FL OsHIPPs and their corresponding isoprenylation mutants (C/S). OsHIPPC/S mutants: OsHIPP14C187S, OsHIPP21C133S, OsHIPP39C190S, OsHIPP41C152S. Protein loading is indicated by Ponceau S staining (PS). c Total yeast protein extracts from haploid yeast were separated on a NuPAGE Bis-Tris gel and analysed by immunoblot using an anti-HA antibody to detect Gal4-AD fusion proteins with MAX effectors. Protein loading is indicated by Ponceau S staining (PS). d Total yeast protein extracts from haploid yeast were separated on a 10 – 20% Tricine SDS-PAGE gel and analysed by immunoblot using an anti-BD antibody to detect Gal4-BD fusion proteins with FL OsHPP09 wildtype (WT) and mutant versions. Protein loading is indicated by Ponceau S staining (PS). (PDF) [file ppat.1014382.s004.pdf]

HMA domain CaaX motif

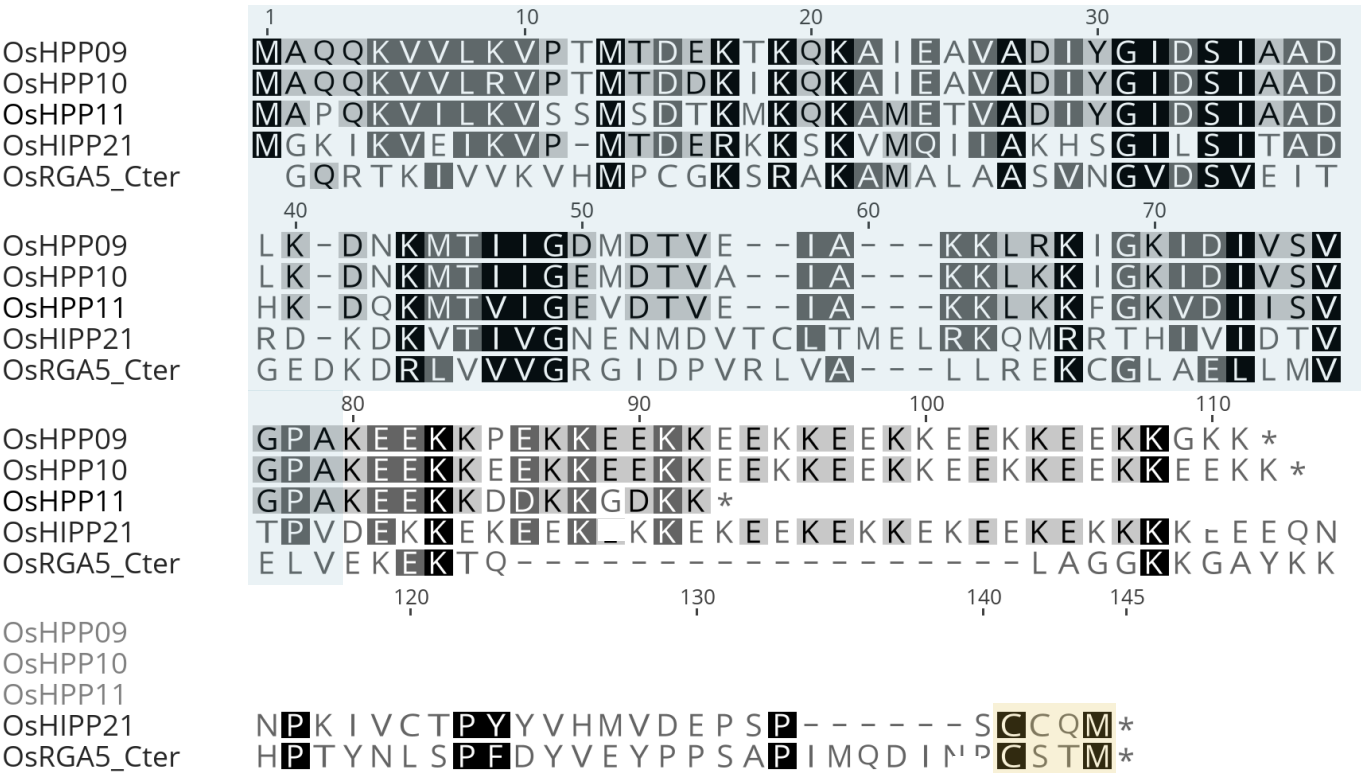

Supplement: S5 Fig — Amino acid alignment of full-length OsHPP09, OsHPP10, OsHPP11 and OsHIPP21 and residues 995–1116 of OsRGA5, including the HMA domain and the C-terminal region of the protein which concludes with a putative CaaX isoprenylation motif (CSTM). Sequence alignment was carried out with Clustal Omega. The blue shaded box highlights the HMA domain, while the yellow shaded box highlights the CaaX motif present in OsHIPP21 and OsRGA5. (PDF) [file ppat.1014382.s005.pdf]

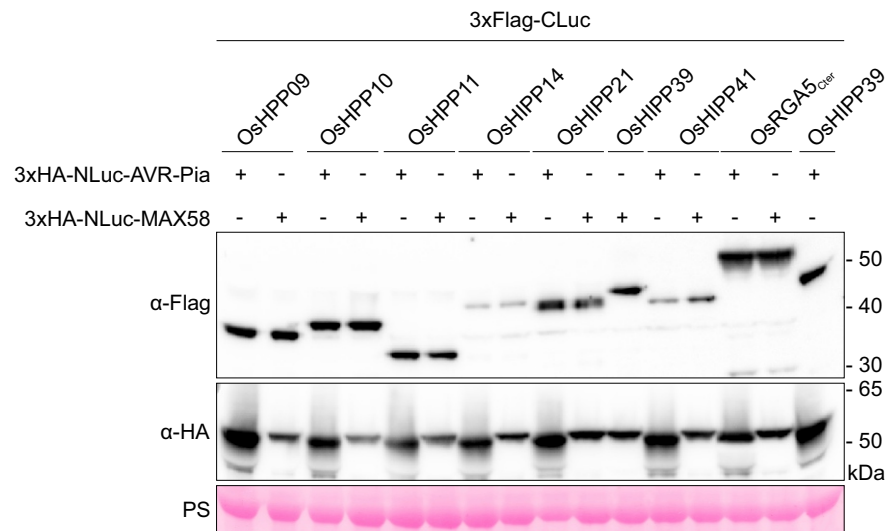

Supplement: S6 Fig — Immunoblot analysis of transiently expressed proteins in N. benthamiana, including OsH(I)PPs N-terminally tagged with a 3xFlag epitope fused to the C-terminal part of luciferase (CLuc), and AVR-Pia and MAX58 N-terminally tagged with 3xHA and the N-terminal part of luciferase (NLuc). This experiment corresponds to replicate 1 of Fig 1. Detection was performed using anti-Flag and anti-HA antibodies. Membrane was stripped after anti-Flag detection and re-probed with anti-HA. Protein loading is indicated by the Rubisco band visualized by Ponceau S. staining (PS). (PDF) [file ppat.1014382.s006.pdf]

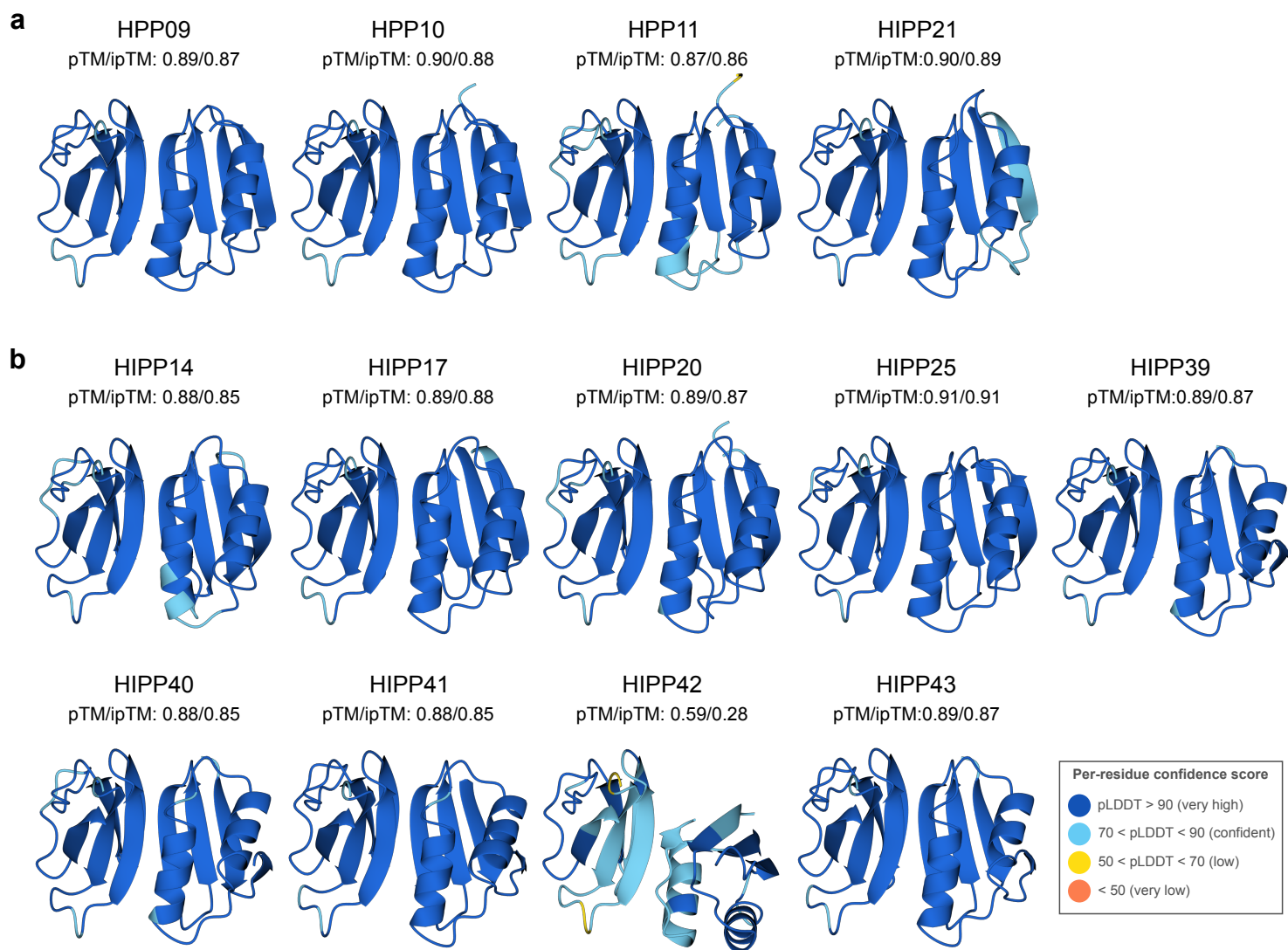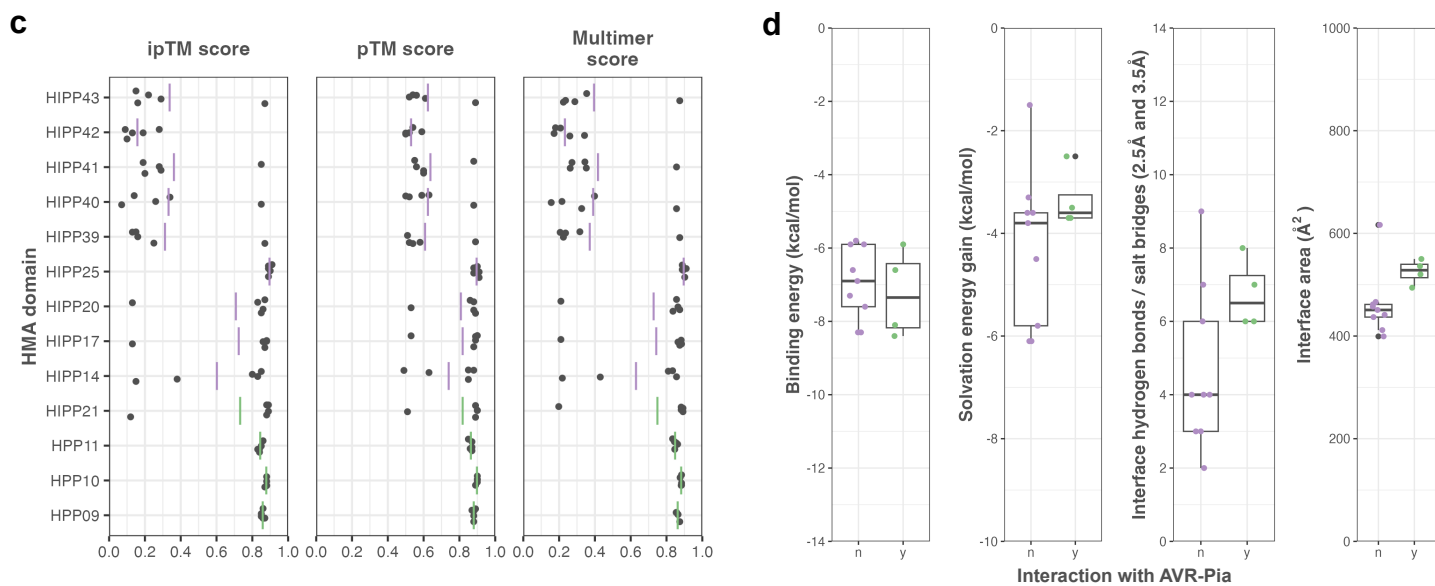

Supplement: S7 Fig — AlphaFold2 (ColabFold v1.5.5) models of AVR-Pia in complex with HMA domains which a were experimentally shown to interact with AVR-Pia and b do not interact with AVR-Pia. Structure models are represented as ribbons and coloured by pLDDT score using the classical AlphaFold colour scheme as shown in the key. c Plots of ipTM, pTM and Multimer (0.8*iPTM + 0.2*pTM) scores for each of the five models generated for each HMA/AVR-Pia complex. Points indicate scores of individual models; green and purple lines indicate mean values for AVR-Pia-interacting and non-interacting HMA domains, respectively. d Comparison of interface parameters determined by qtPISA [57]) for the top ranked model of each HMA/AVR-Pia complex. Green and purple points indicate values for individual HMA/AVR-Pia models. The centre line of the box represents the median and the limits of the box represent the upper and lower quartiles. Whiskers extend to the smallest value within (Q1 − 1.5 × the interquartile range (IQR)) and the largest value within (Q3 + 1.5 × IQR). (PDF) [file ppat.1014382.s007.pdf]

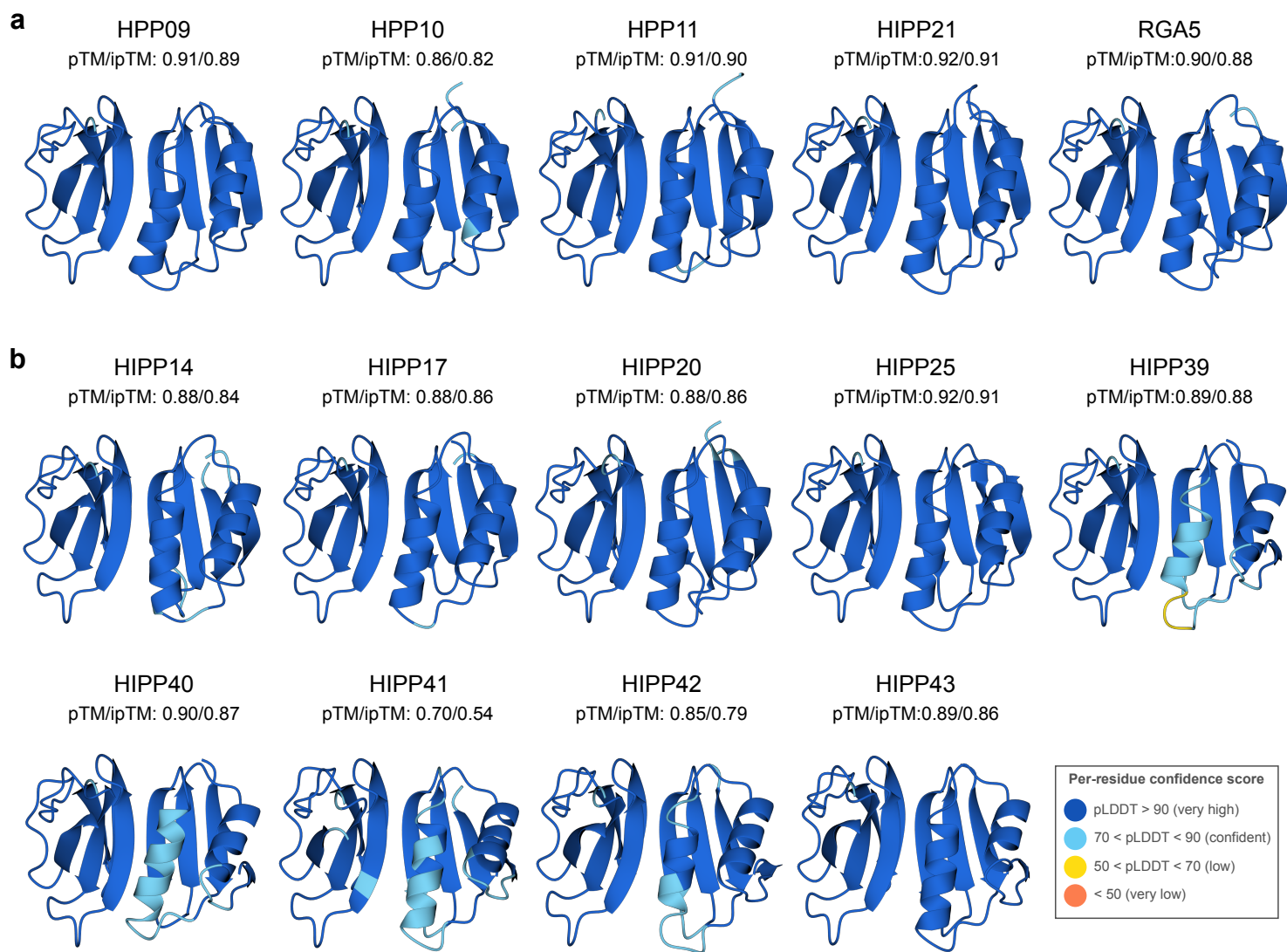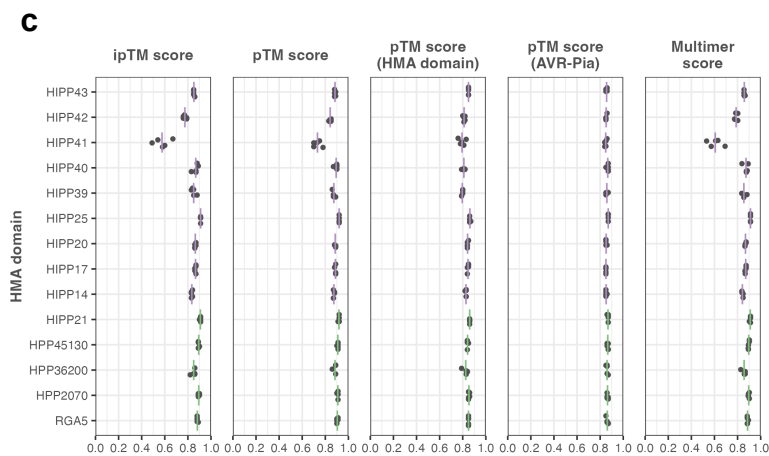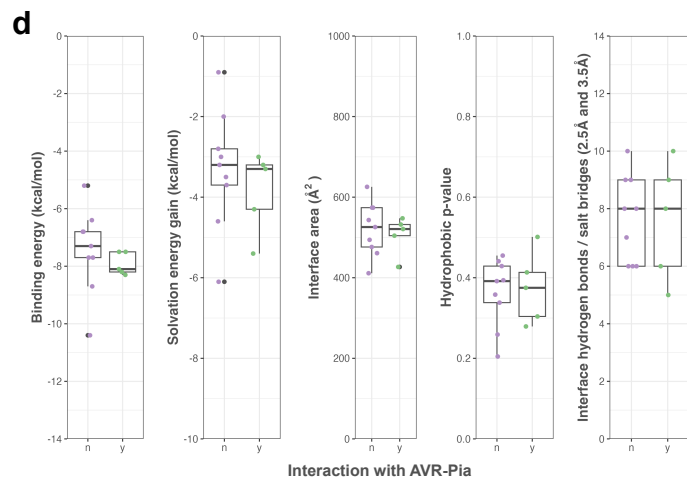

Supplement: S8 Fig — AlphaFold3 models of AVR-Pia in complex with HMA domains which a were experimentally shown to interact with AVR-Pia and b do not interact with AVR-Pia. Structure models are represented as ribbons and coloured by pLDDT score using the classical AlphaFold colour scheme as shown in the key. c Plots of ipTM, pTM and Multimer (0.8*iPTM + 0.2*pTM) scores for each of the top ranked models from each of the five seeds used to model each HMA/AVR-Pia complex. Points indicate scores of individual models; green and purple lines indicate mean values for AVR-Pia-interacting and non-interacting HMA domains, respectively. d Comparison of interface parameters determined by qtPISA [57]) for the top ranked model (selected from models from all five seeds) of each HMA/AVR-Pia complex. Green and purple points indicate values for individual HMA/AVR-Pia models. The centre line of the box represents the median and the limits of the box represent the upper and lower quartiles. Whiskers extend to the smallest value within (Q1 − 1.5 × the interquartile range (IQR)) and the largest value within (Q3 + 1.5 × IQR). (PDF) [file ppat.1014382.s008.pdf]

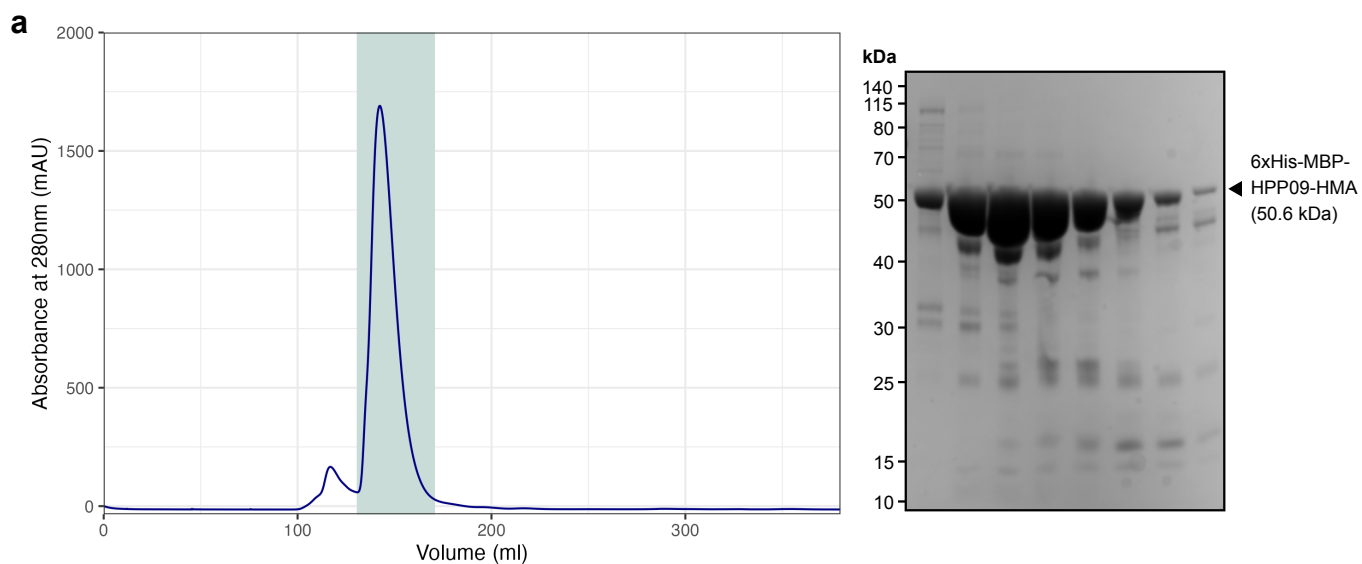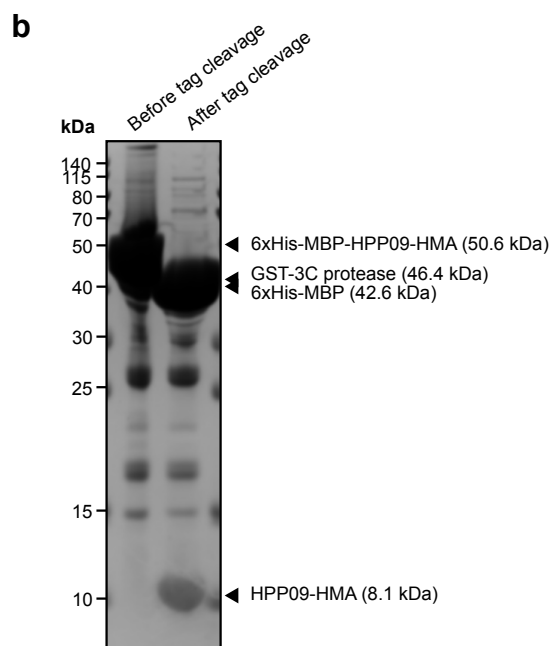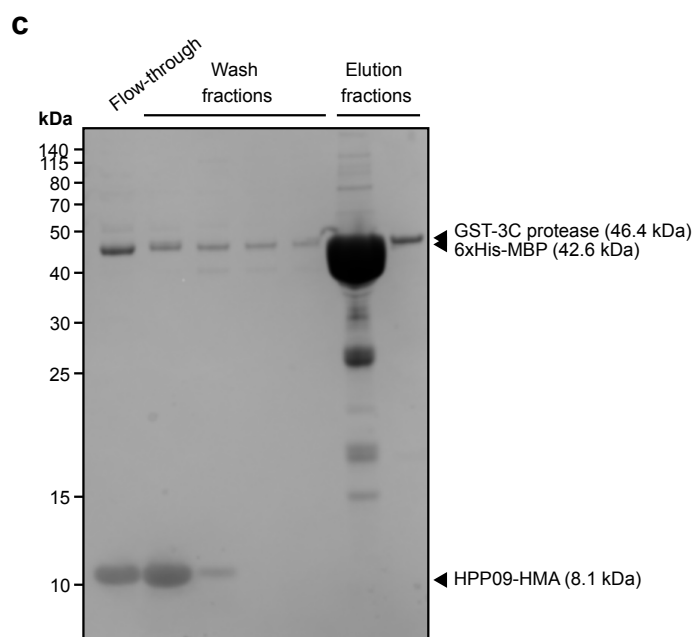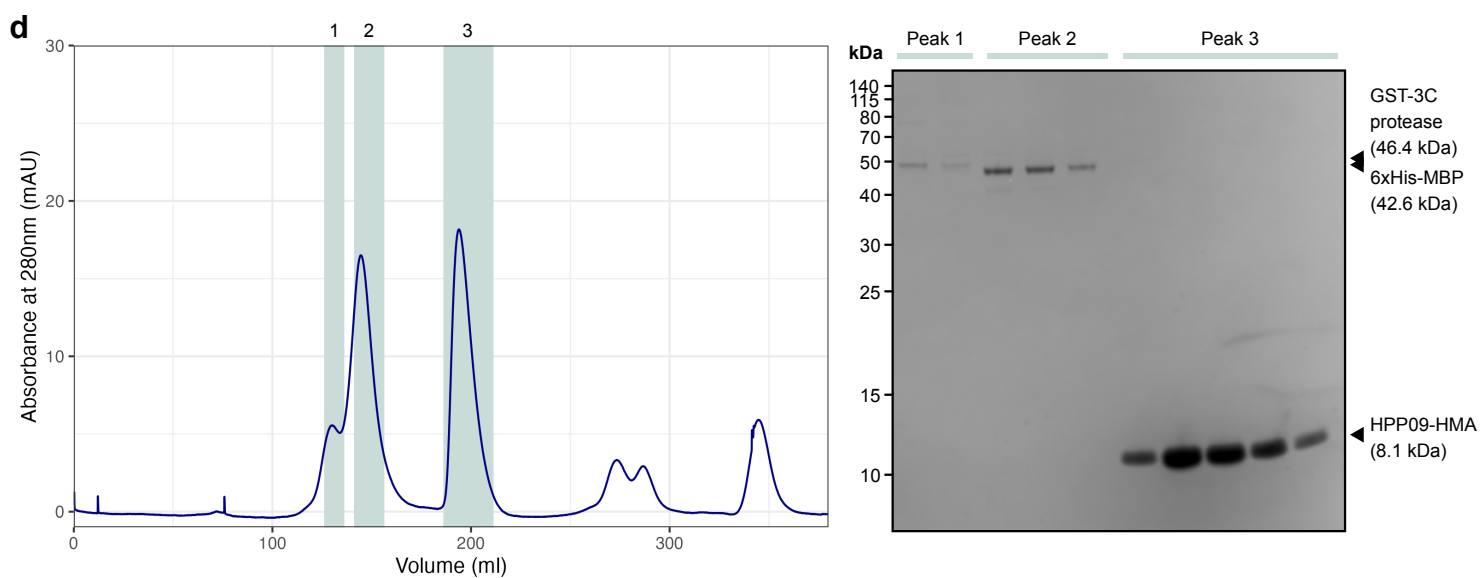

Supplement: S9 Fig — a Size exclusion chromatography elution trace for 6xHis-MBP-OsHPP09-HMA (following the initial IMAC purification step). The SDS-PAGE gel shows fractions corresponding to the peak in the shaded area. b SDS-PAGE gel of pooled fractions following size exclusion chromatography before and after cleavage of the 6xHis-MBP tag with GST-tagged 3C protease. c SDS-PAGE gel showing flow-through, wash and elution fractions from tandem HisTrap, MBPTrap and GSTrap affinity columns. d Size exclusion chromatography elution trace for OsHPP09-HMA. OsHPP09-HMA absorbs light at 280 nm poorly (molar extinction coefficient of 1490 M-1 cm-1). The SDS-PAGE gel shows fractions corresponding to the three peaks in the shaded areas. The peak elution volume of OsHPP09-HMA (193.9 ml) is consistent with dimerisation of the HMA domain. (PDF) [file ppat.1014382.s009.pdf]

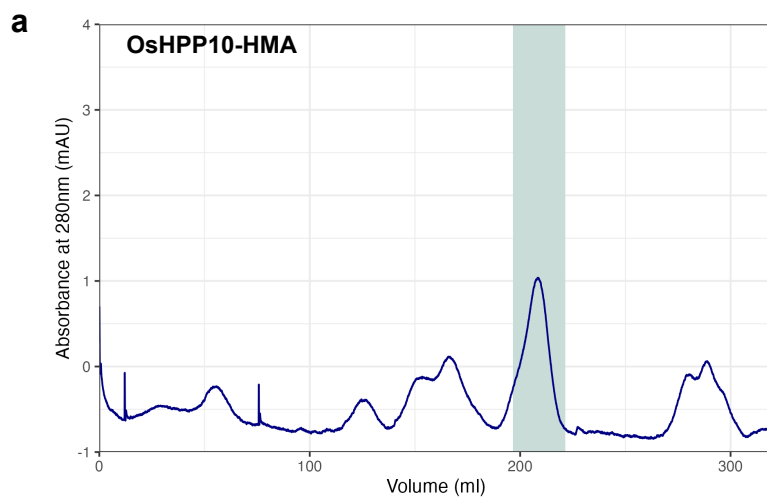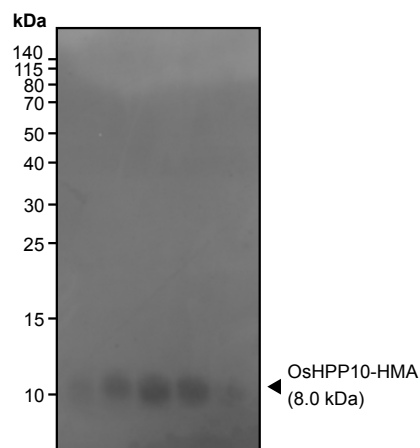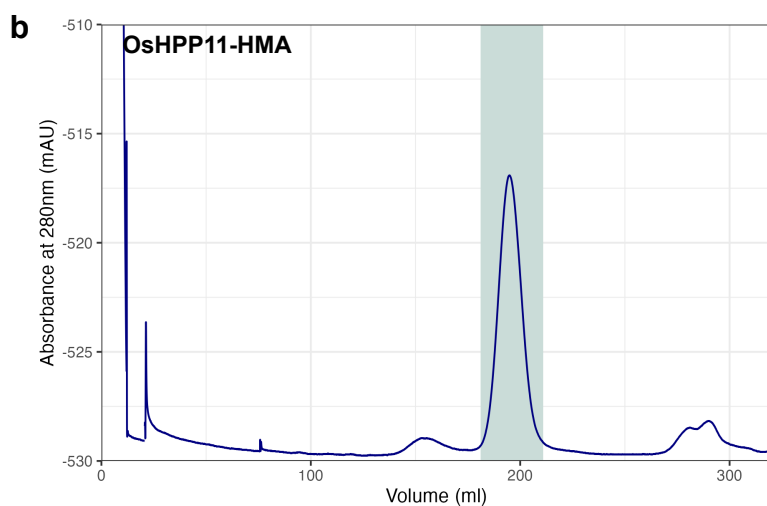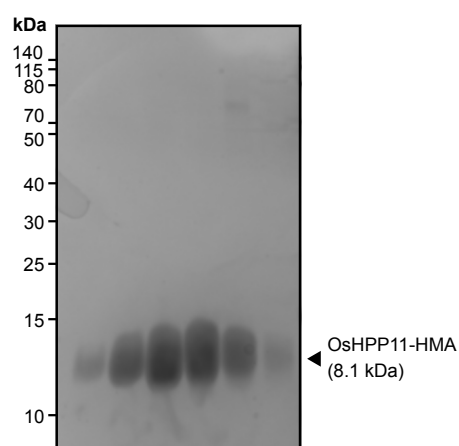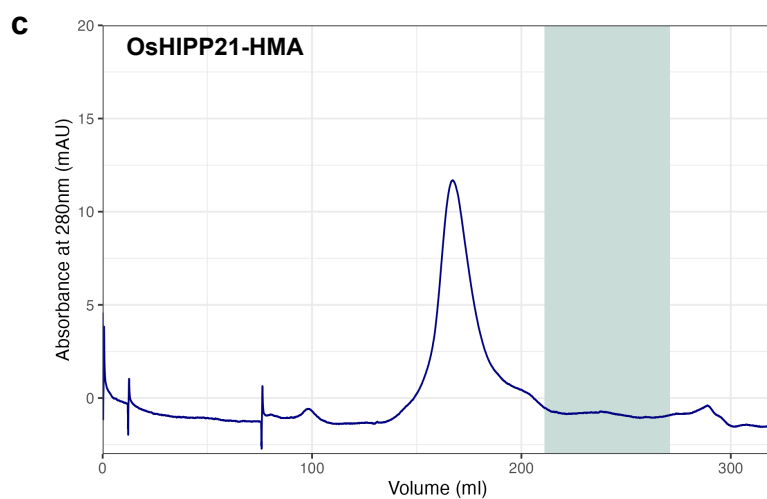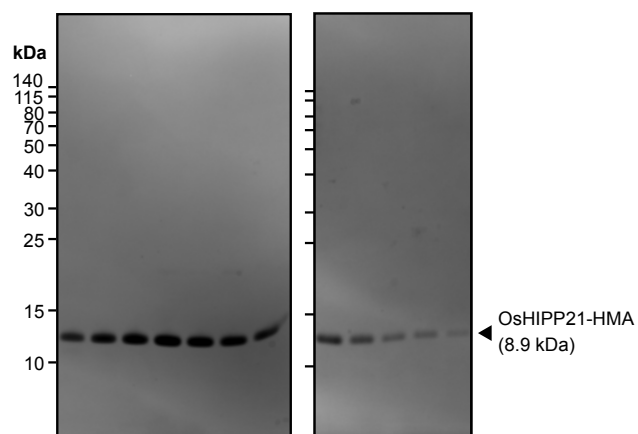

Supplement: S10 Fig — Representative size exclusion chromatography elution traces for a OsHPP10-HMA, b OsHPP11-HMA and c OsHIPP21-HMA. All three HMA domains absorb light at 280 nm poorly (molar extinction coefficients of 1490 M-1 cm-1, 1490 M1 cm-1 and 0 M1 cm-1, respectively) so the corresponding peaks are small for OsHPP10-HMA and OsHPP11-HMA and negligible for OsHIPP21). SDS-PAGE gels show fractions corresponding to the shaded areas of the trace. These fractions were pooled and concentrated to obtain the purified protein for subsequent analyses. (PDF) [file ppat.1014382.s010.pdf]

**a**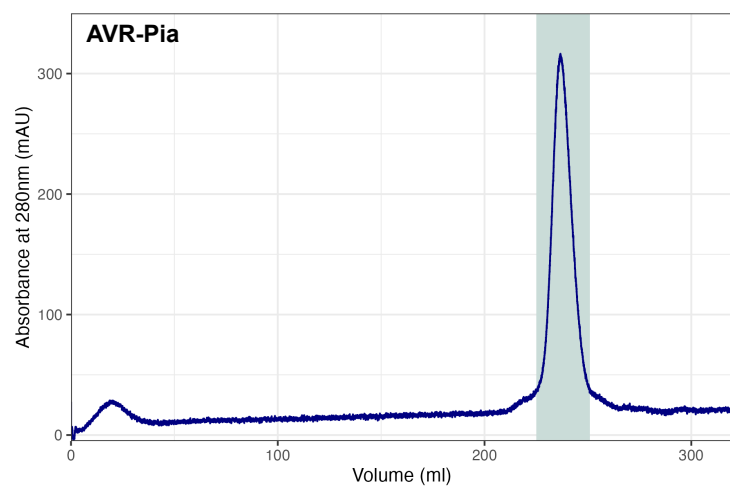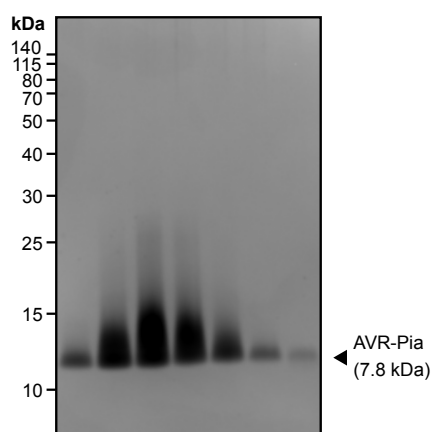**b**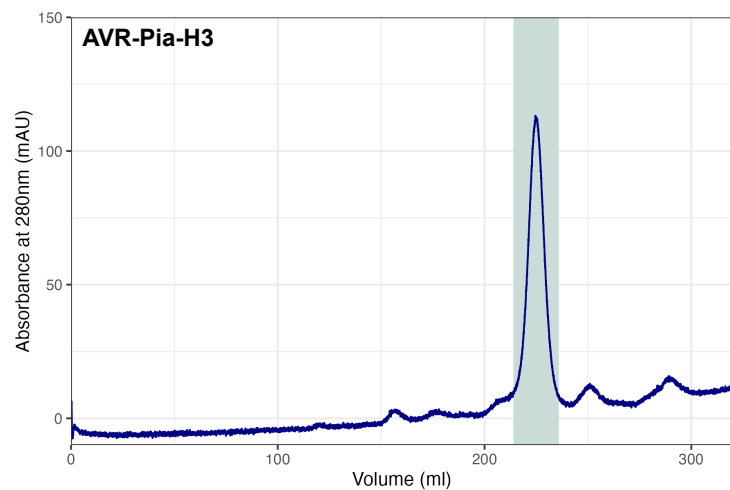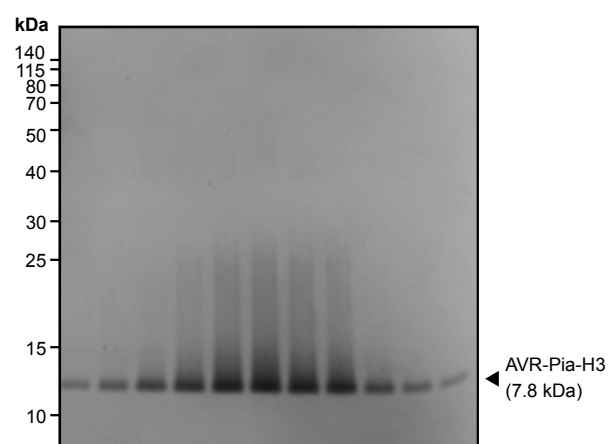**c**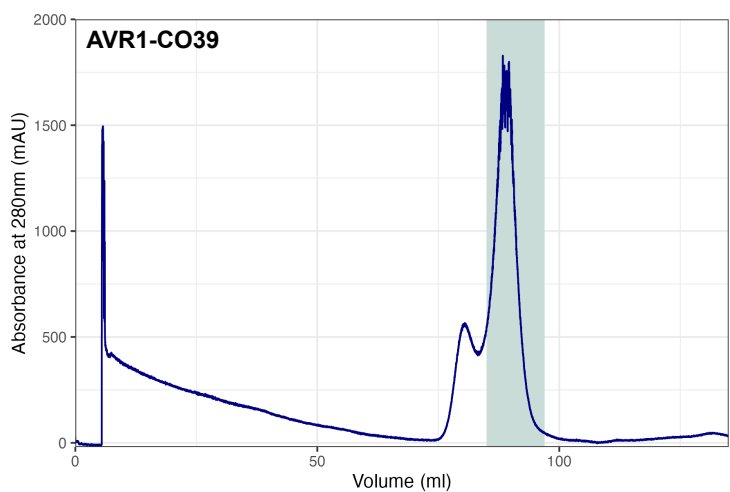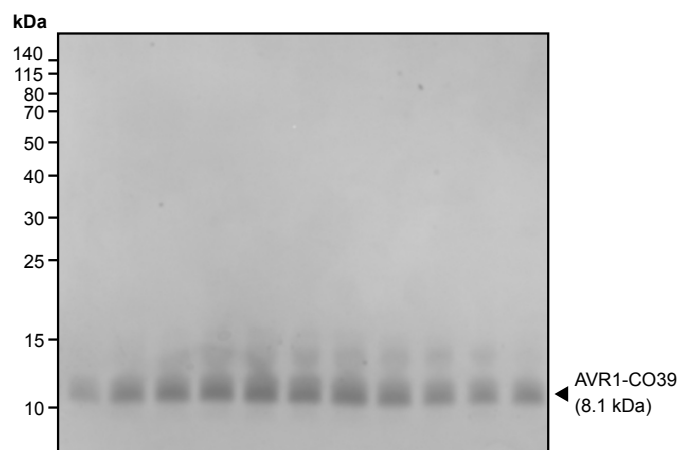

Supplement: S11 Fig — Representative size exclusion chromatography elution traces for a AVR-Pia, b AVR-Pia-H3 and c AVR1-CO39. SDS-PAGE gels show fractions corresponding to the peaks in the shaded areas of the trace. These fractions were pooled and concentrated to obtain the purified protein for subsequent analyses. (PDF) [file ppat.1014382.s011.pdf]

Replicate 2

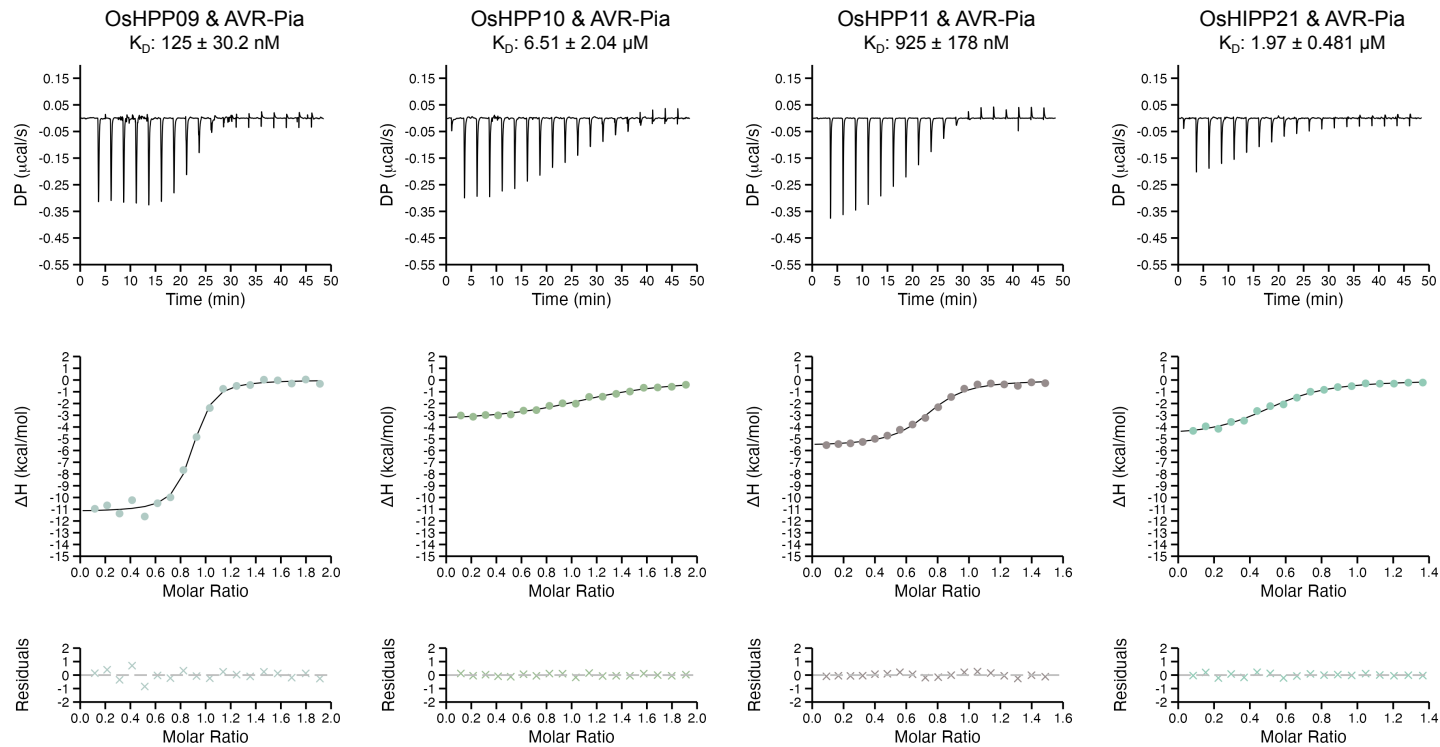

Replicate 3

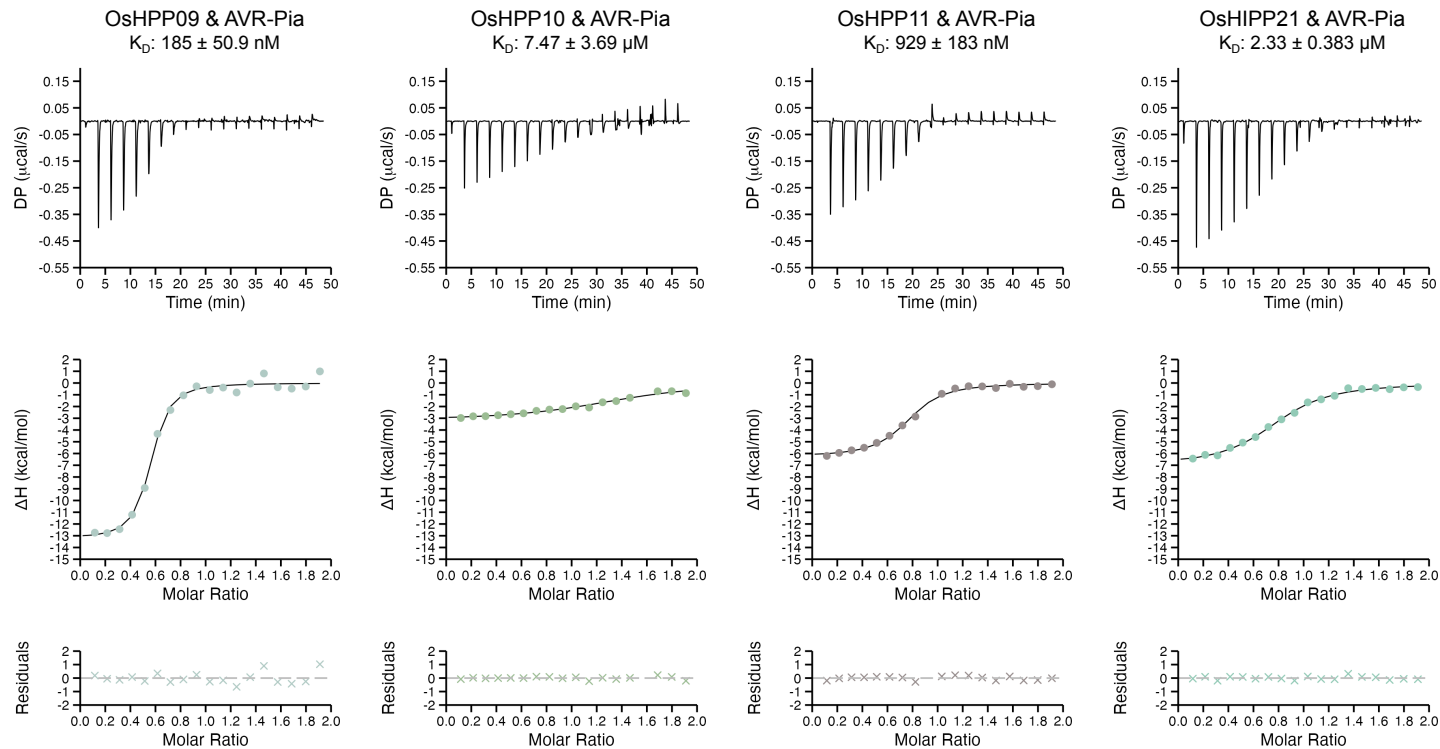

Supplement: S13 Fig — Top panels show the raw thermograms obtained from titration of AVR-Pia into a solution containing the purified HMA domains. Central panels show the integrated heats (coloured dots) and binding isotherms fitted to a single site model (black line) using the MicroCal PEAQ-ITC analysis software (Malvern Panalytical). Bottom panels show the differences (coloured crosses) between the modelled and observed values (residuals). (PDF) [file ppat.1014382.s013.pdf]

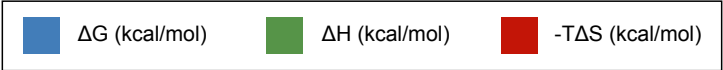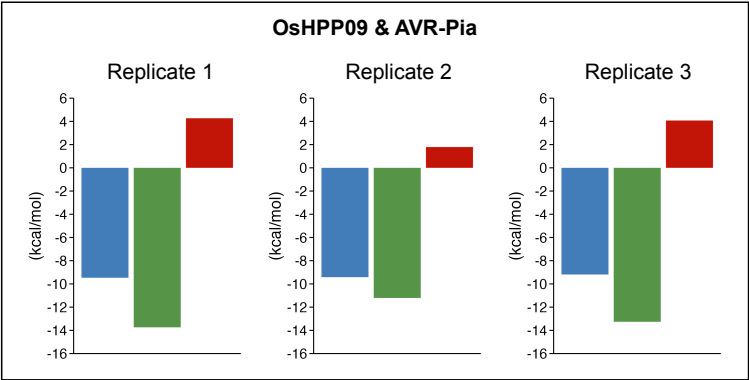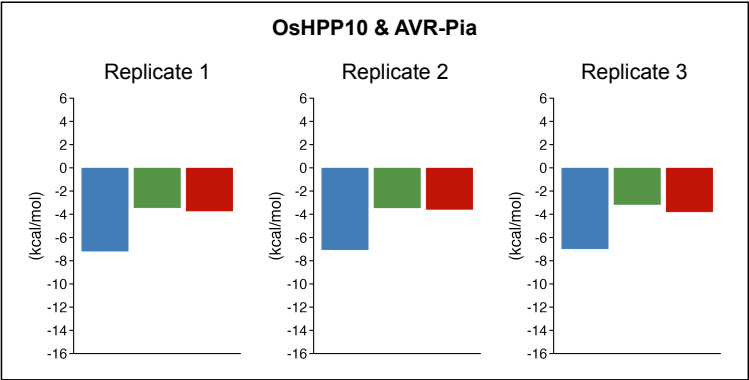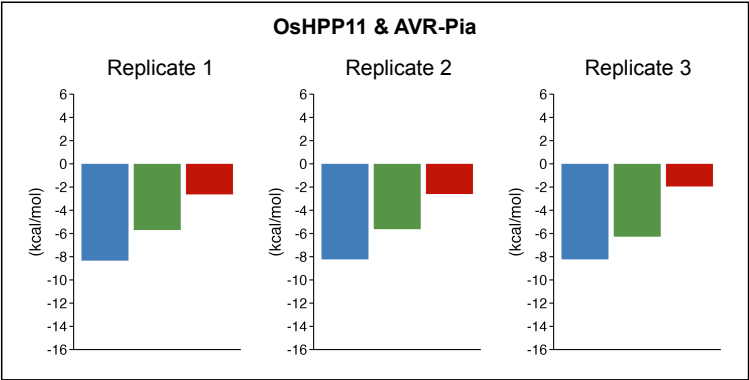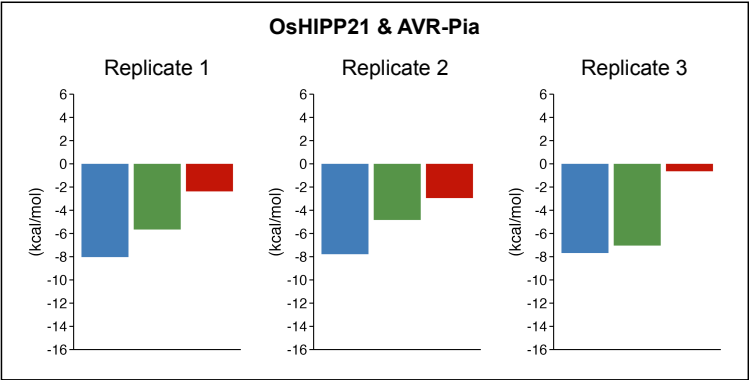

Supplement: S14 Fig — Bars represent the magnitude of the determined thermodynamic parameters ΔG (blue bar), ΔH (green bar) and -TΔS (red bar). (PDF) [file ppat.1014382.s014.pdf]

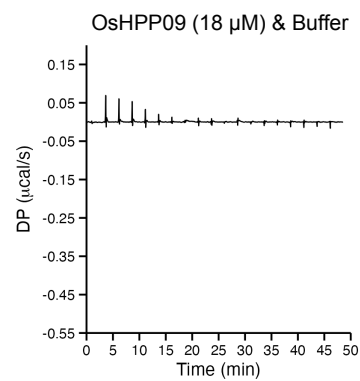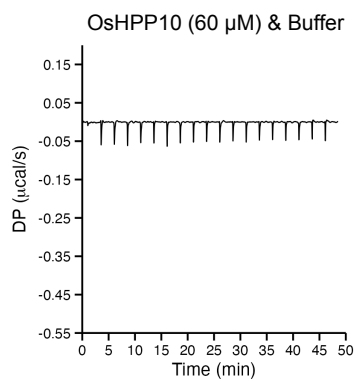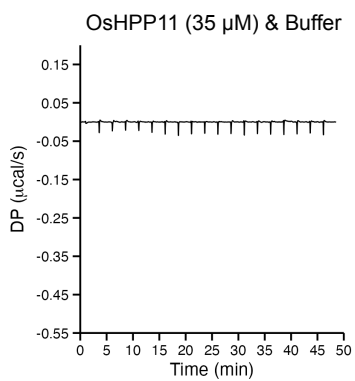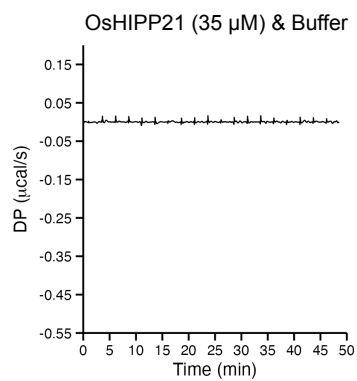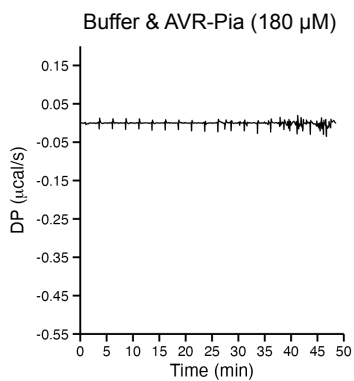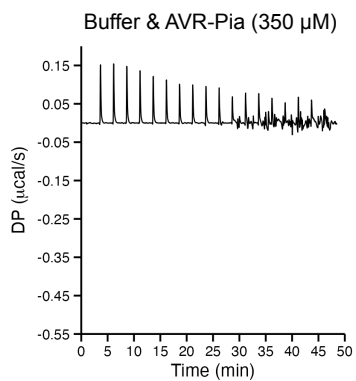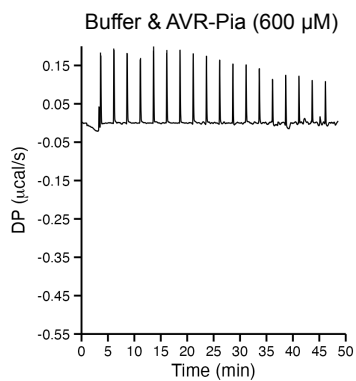

Supplement: S15 Fig — Representative raw thermograms from titration of buffer into a solution containing the purified HMA domains (top row) and titration of AVR-Pia into buffer (bottom row) at the concentrations used in the experiments presented in Figs 2 and S13. (PDF) [file ppat.1014382.s015.pdf]

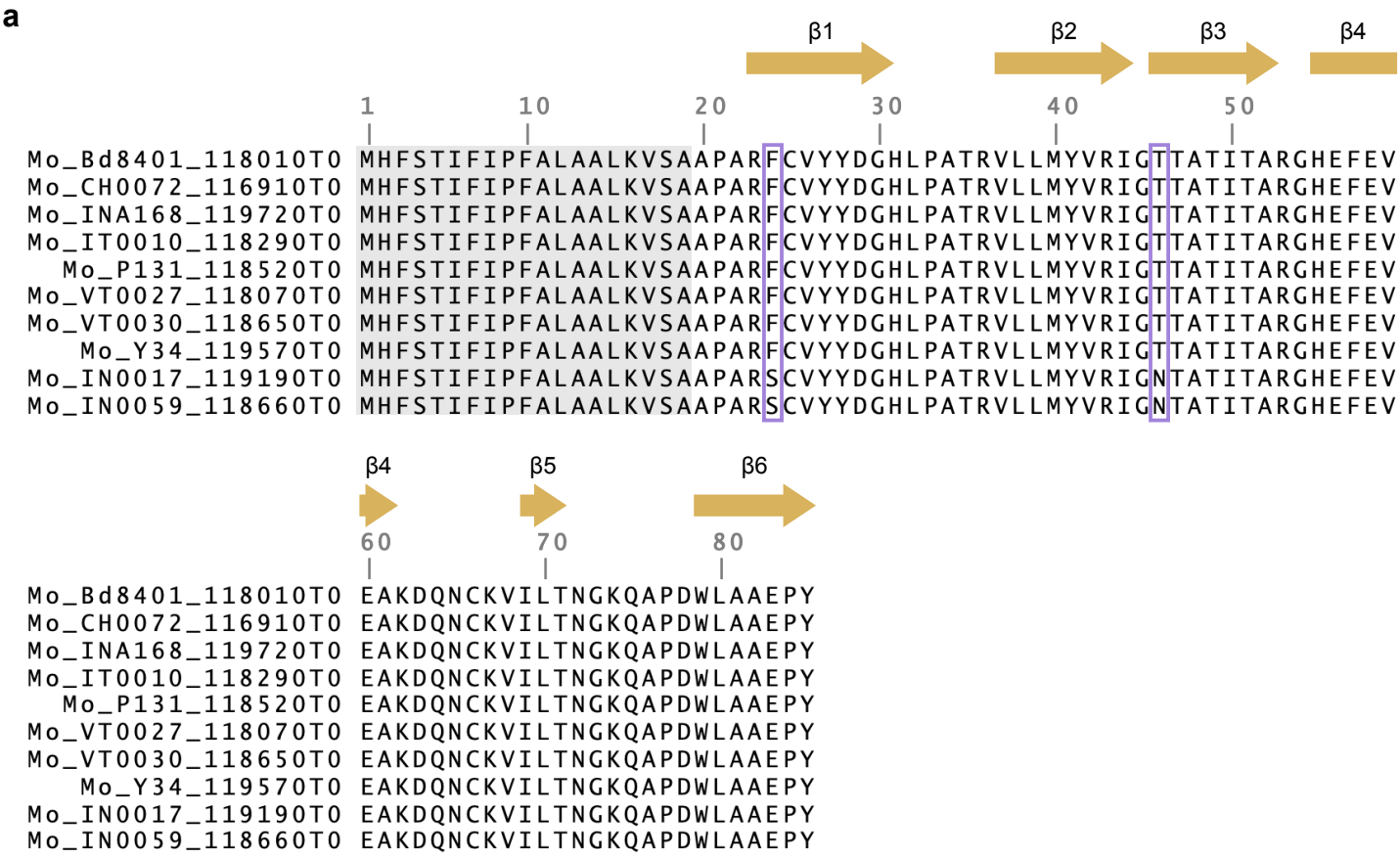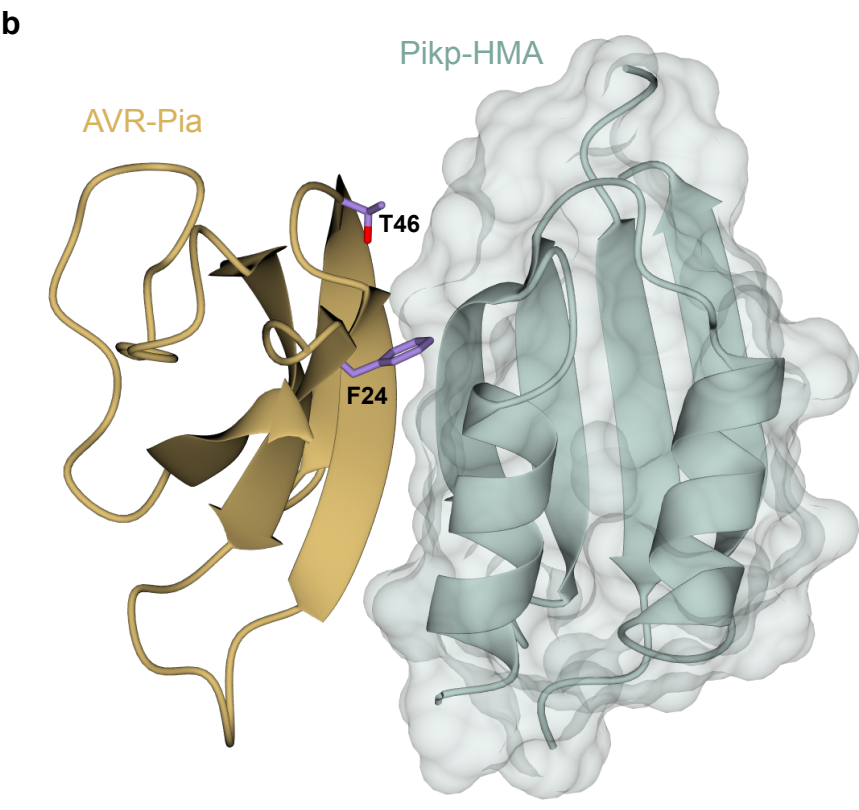

Supplement: S16 Fig — a Multiple sequence alignment of AVR-Pia variants from Magnaporthe oryzae isolates described in [7]. Signal peptide is highlighted by a shaded grey box. Polymorphic residues 24 (F, S) and 46 (T, N) are indicated in a purple outlined box. Arrows above the alignment indicate secondary structure elements. b The polymorphic residues F24 and T46 are located at the HMA binding interface. Crystal structure of AVR-Pia in complex with Pikp-1-HMA (PDB 6Q76 [48]). AVR-Pia and Pikp-1-HMA are represented as gold and teal ribbons, respectively, with the side chains of AVR-Pia T46 and F24S shown in purple. (PDF) [file ppat.1014382.s016.pdf]

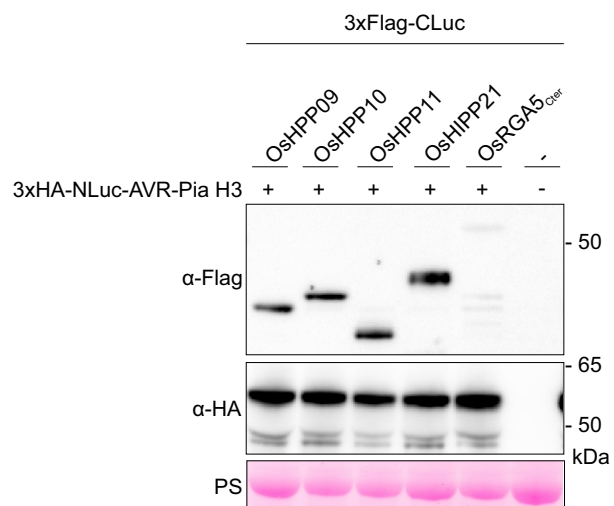

Supplement: S17 Fig — Immunoblot analysis of transiently expressed proteins in N. benthamiana, including OsH(I)PPs and OsRGA5Cter (883 – 1116 aa) N-terminally tagged with a 3xFlag epitope fused to the C-terminal part of luciferase (CLuc), and AVR-Pia-H3 N-terminally tagged with 3xHA and the N-terminal part of luciferase (NLuc). This experiment corresponds to replicate 2 of Fig 3. Detection was performed using anti-Flag and anti-HA antibodies. Membrane was stripped after anti-Flag detection and re-probed with anti-HA. Protein loading is indicated by the Rubisco band visualized by Ponceau S. staining (PS). S18 Fig. Expression of CLuc- and NLuc fusion proteins (related to Fig 4). (PDF) [file ppat.1014382.s017.pdf]

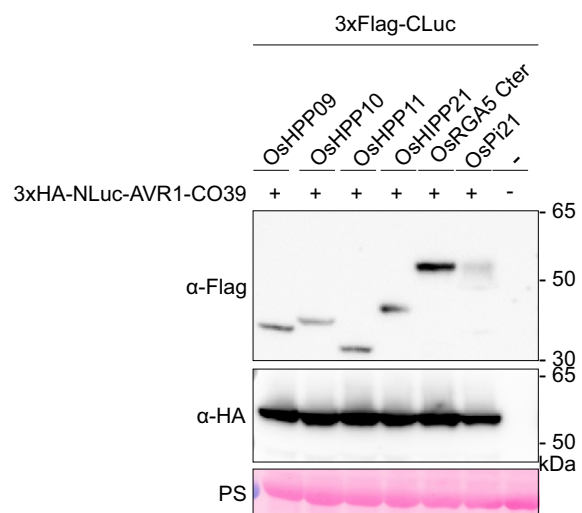

Supplement: S18 Fig — This experiment corresponds to replicate 1 of Fig 4. Detection was performed using anti-Flag and anti-HA antibodies. Membrane was stripped after anti-Flag detection and re-probed with anti-HA. Protein loading is indicated by the Rubisco band visualized by Ponceau S. staining (PS). (PDF) [file ppat.1014382.s018.pdf]

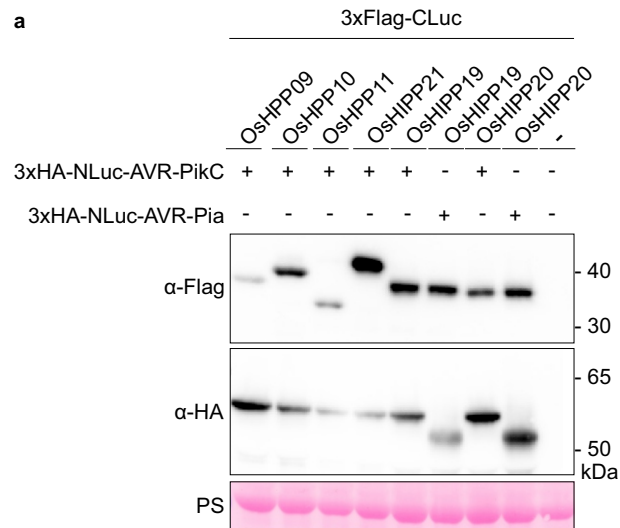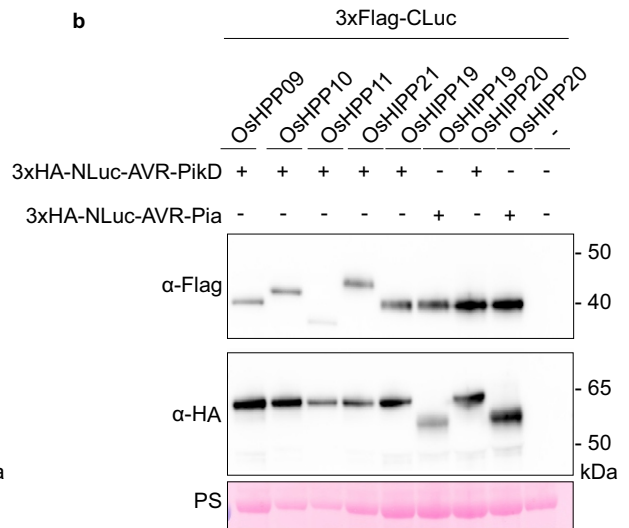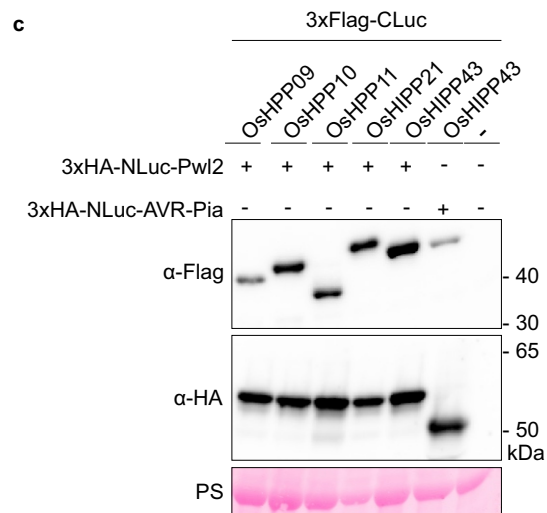

Supplement: S19 Fig — Immunoblot analysis of transiently expressed proteins in N. benthamiana, including OsH(I)PPs N-terminally tagged with a 3xFlag epitope fused to the C-terminal part of luciferase (CLuc), and AVR-Pia, AVR-PikC, AVR-PikD and Pwl2 N-terminally tagged with 3xHA and the N-terminal part of luciferase (NLuc). This experiment corresponds to replicate 1 of Fig 5. Detection was performed using anti-Flag and anti-HA antibodies. Membrane was stripped after anti-Flag detection and re-probed with anti-HA. Protein loading is indicated by the Rubisco band visualized by Ponceau S. staining (PS). (PDF) [file ppat.1014382.s019.pdf]

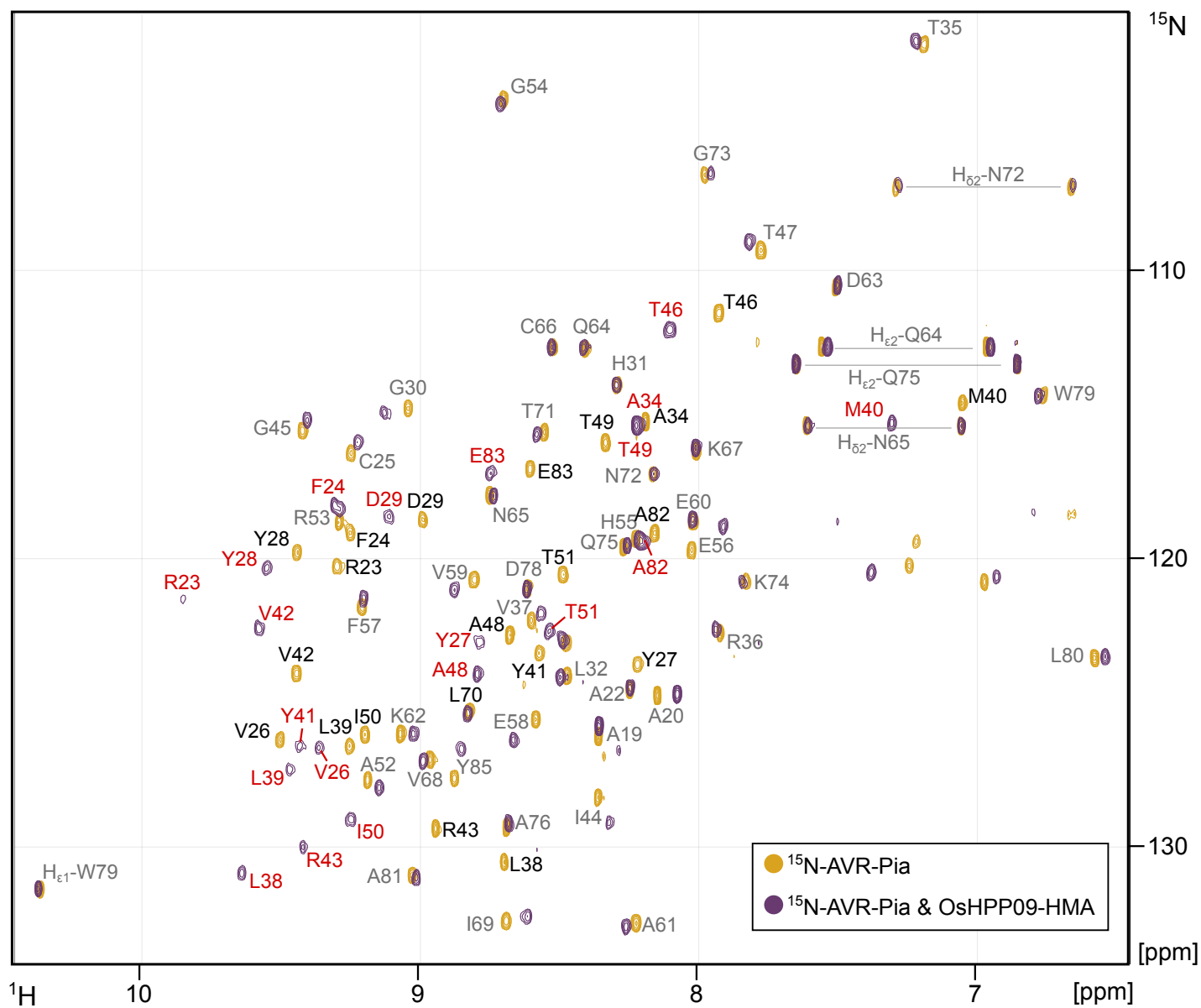

Supplement: S20 Fig — Grey cross-peak labels are used for residues with little or no chemical shift perturbation. For substantial chemical shifts, black cross-peak labels correspond to spectra for 15N-AVR-Pia alone (previously assigned [4]) and red cross-peak labels correspond to spectra for 15N-AVR-Pia in the presence of OsHPP09-HMA. (PDF) [file ppat.1014382.s020.pdf]

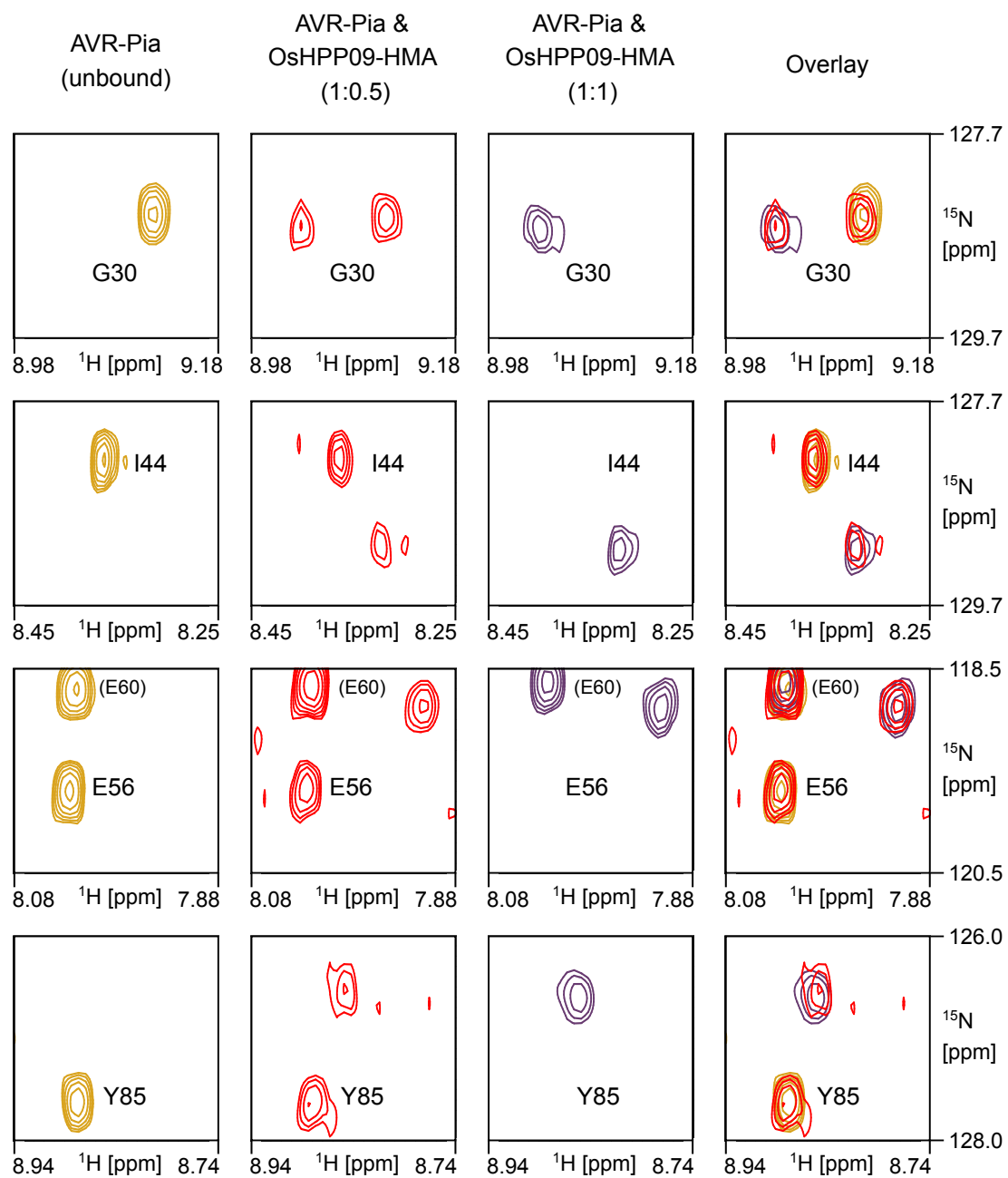

Supplement: S21 Fig — Cross-peaks corresponding to residues G30, I44, E56 and Y85 extracted from [1H,15N] HSQC spectra from NMR titrations performed with 15N-labelled AVR-Pia and OsHPP09-HMA at molar ratios of 1:0 (free AVR-Pia; orange cross-peaks), 1:0.5 (intermediate; red cross-peaks) and 1:1 (bound AVR-Pia; purple cross-peaks). The final column shows the overlay of the different spectra. (PDF) [file ppat.1014382.s021.pdf]

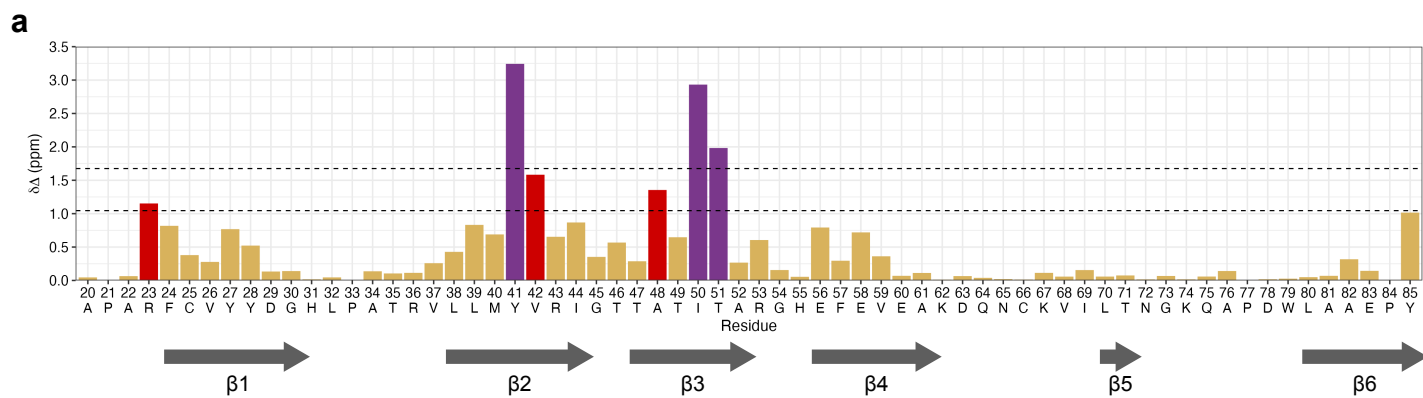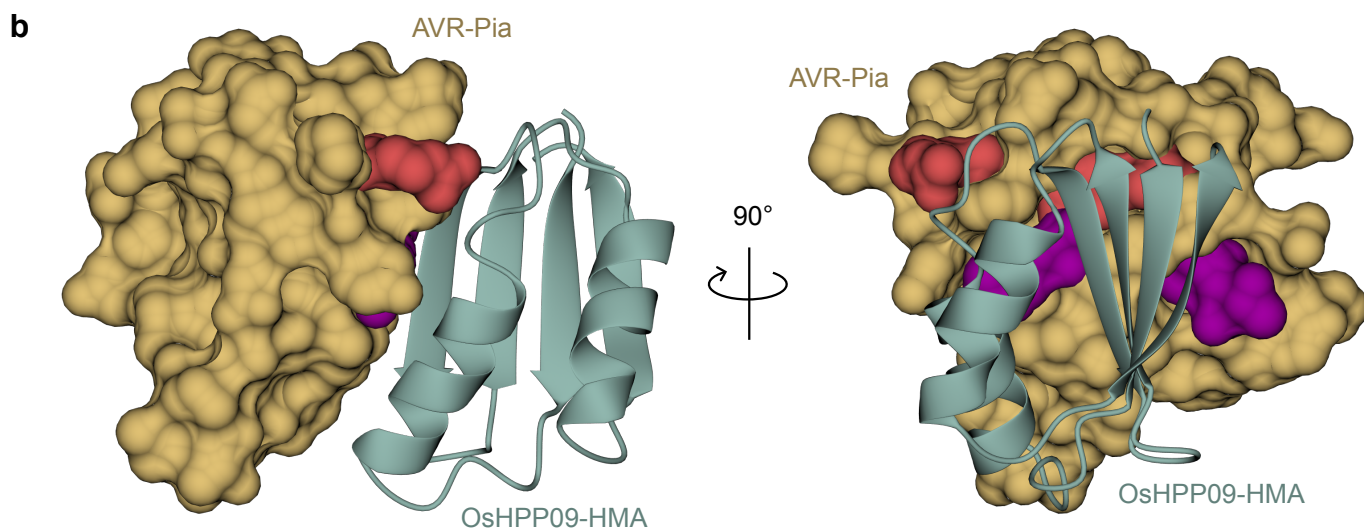

Supplement: S22 Fig — a Barplot of 1H/15N chemical shift perturbations (Δδ) for the amide groups of AVR-Pia. Dashed lines represent Δδ thresholds of Δδ― + 1σ and Δδ― + 2σ. Significant chemical shift variations (> Δδ― + 1σ or> Δδ― + 2σ) are indicated in red and purple, respectively. b Residues giving significant chemical shift variations of> Δδ― + 1σ or> Δδ― + 2σ represented in red or purple, respectively, on the crystal structure (PDB 9RSV) of AVR-Pia (surface representation) bound to OsHPP09-HMA (teal ribbon representation). The two views are rotated by 90° as indicated. (PDF) [file ppat.1014382.s022.pdf]

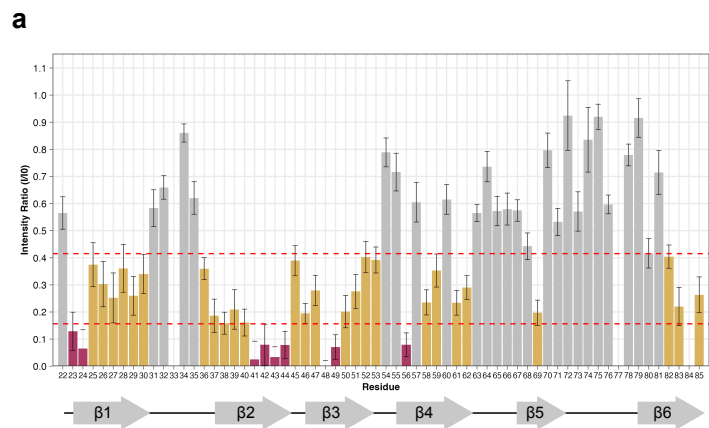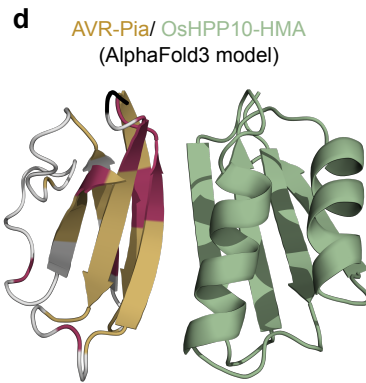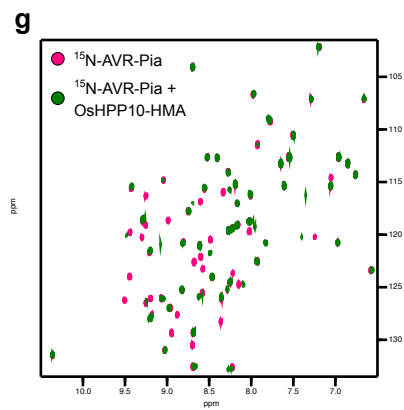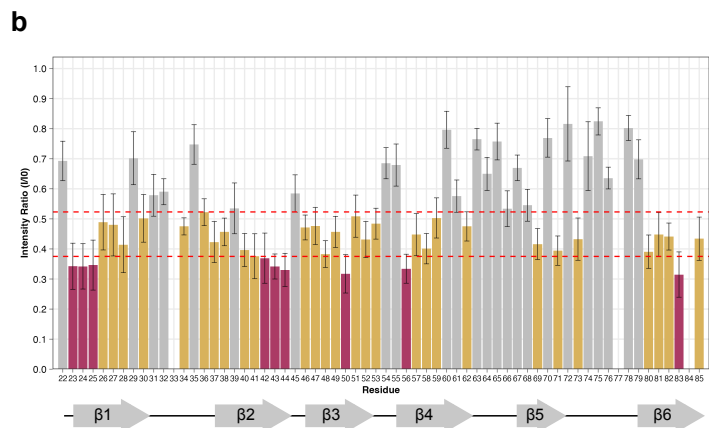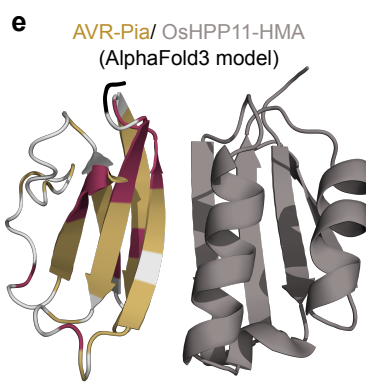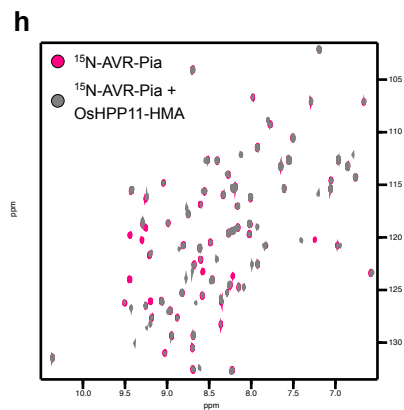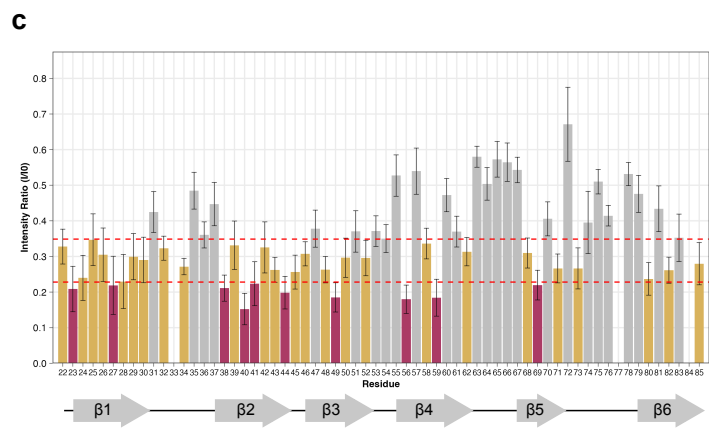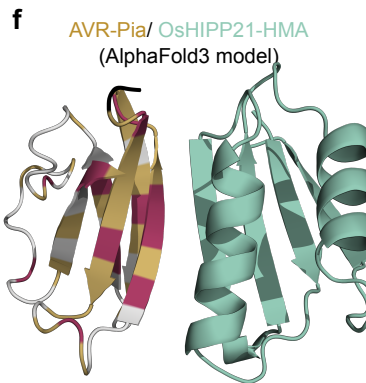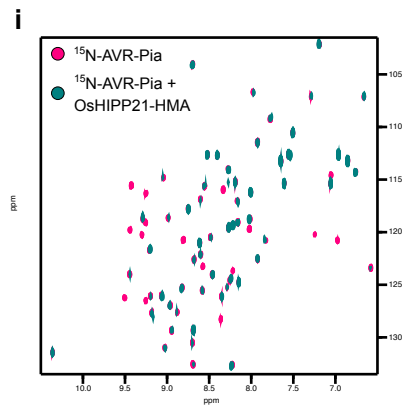

Supplement: S23 Fig — a-c Residue-resolved NMR signal intensity ratios I/I0, where I0 represents the signal intensity for free AVR-Pia and I represents the signal intensity for AVR-Pia bound to OsHPP10-HMA (a), OsHPP11-HMA (b) or OsHIPP21-HMA (c). The boundaries of the six β-strands of the AVR-Pia MAX fold are shown at the bottom of the plot (grey arrows). Bars are coloured grey for values of I/I0 > x̄, gold if x̄ > I/I0> (x̄ - σ), and pink if I/I0 <(x̄ - σ). Red dashed lines indicate I/I0 thresholds x̄ (upper) and x̄-σ (lower). d-f Ribbon representation of AlphaFold3 models of the complexes between AVR-Pia and OsHPP10-HMA (d), OsHPP11-HMA (e) or OsHIPP21-HMA (f). AVR-Pia residues are coloured by I/I0. g-i Overlay of HSQC spectra of 40 μM 15N-AVR-Pia alone (pink) and presence of 40 μM unlabelled OsHPP10-HMA (green) (g), OsHPP11-HMA (grey) (h) or OsHIPP21-HMA (cyan) (i). Upon complex formation with AVR-Pia, the majority of peaks for residues in β2 and β3 were broadened out beyond detection, whereas residues on the opposite face of AVR-Pia were less affected. (PDF) [file ppat.1014382.s023.pdf]

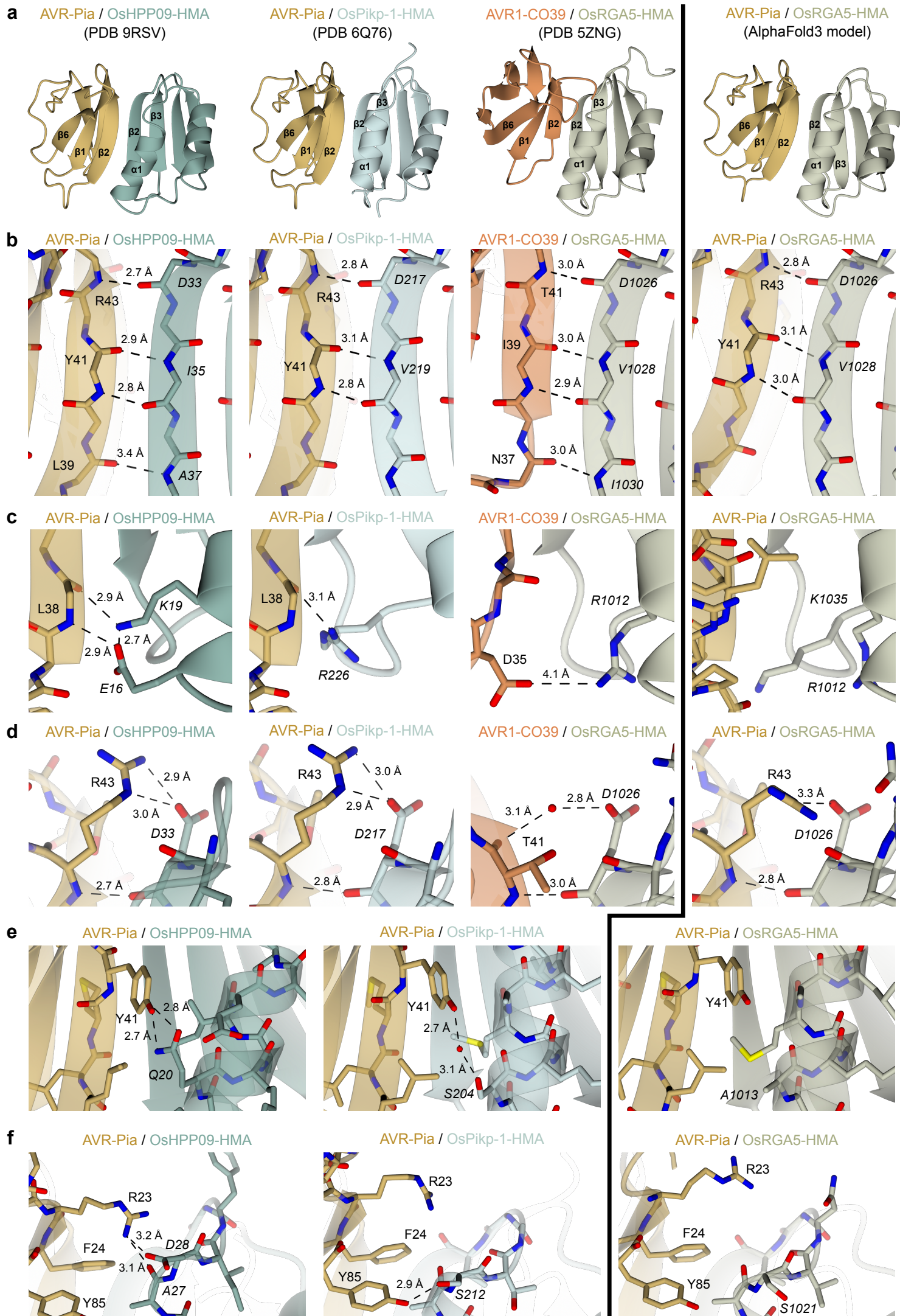

Supplement: S24 Fig — Structures are represented as gold (AVR-Pia), teal (OsHPP09-HMA), brown (AVR-Pia), pale blue (Pikp-1-HMA), orange (AVR1-CO39) and green (RGA5-HMA) ribbons with relevant secondary structure elements indicated. b Hydrogen bonds between residues in β2 of the effector and β2 of the HMA domain. Main chain atoms are represented as cylinders (only the main chain, and not the side chains, are included for clarity). Hydrogen bonds are represented as black dashed lines with lengths (determined by qtPISA [57]) indicated. c-f Comparison of residues involved in forming intermolecular contacts at the effector/HMA interface. Structures are presented in ribbon representation with relevant residues shown as cylinders. Hydrogen bonds are represented as black dashed lines with lengths (determined by qtPISA [57]) indicated. Water molecules are represented as red spheres. (PDF) [file ppat.1014382.s024.pdf]

**a**AVR-Pia / OsHPP09-HMA  
(PDB 9RSV)AVR-Pia / OsHPP09-HMA  
(AlphaFold2 model)AVR-Pia / OsHPP09-HMA  
(AlphaFold3 model)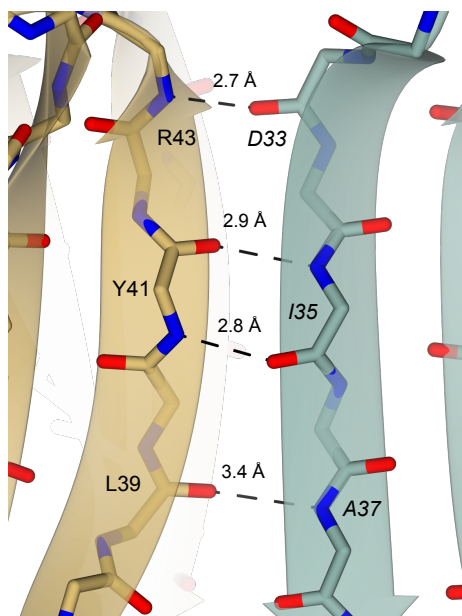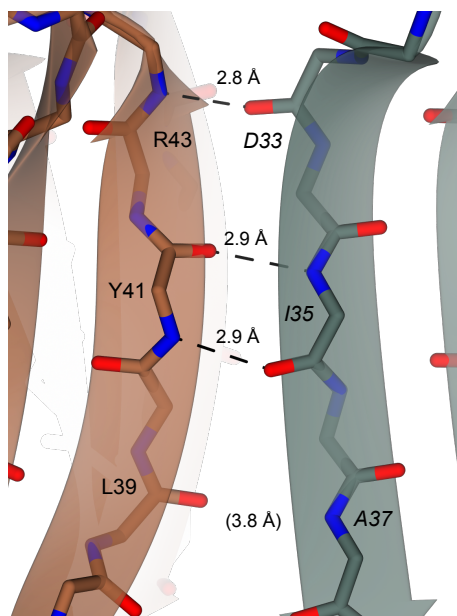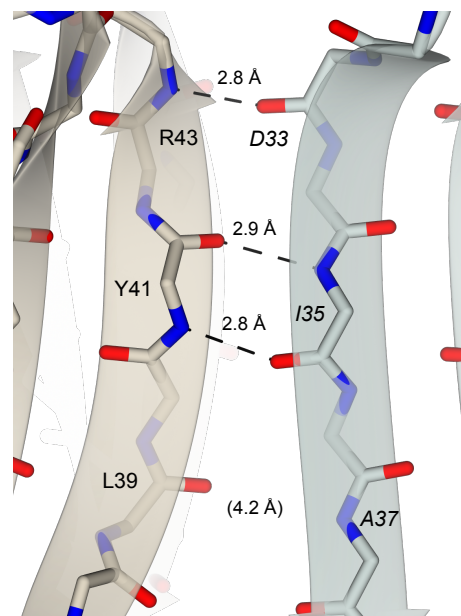**b**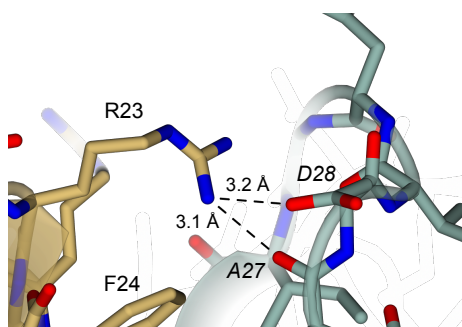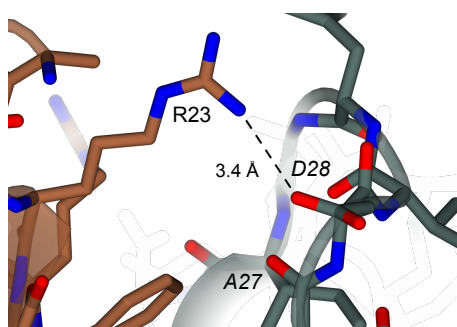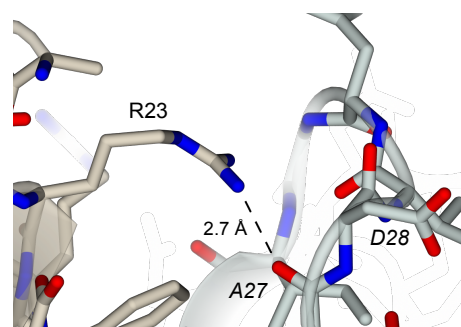**c**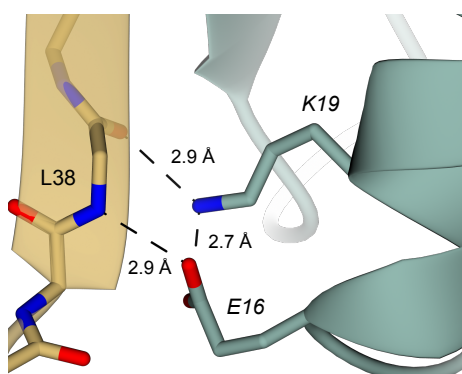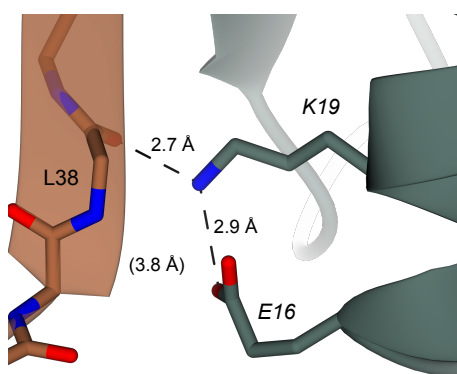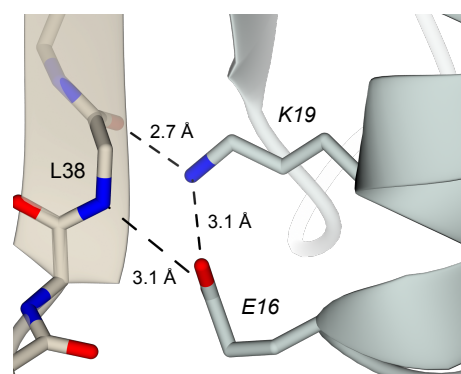

Supplement: S25 Fig — a Hydrogen bonds between residues in β2 of the effector and β2 of the HMA domain. Structures are represented as gold (AVR-Pia, experimental structure), teal (OsHPP09-HMA, experimental structure), brown (AVR-Pia, AlphaFold2 model), dark teal (OsHPP09-HMA, AlphaFold2 model), pale brown (AVR-Pia, AlphaFold3 model) and pale blue (OsHPP09-HMA, AlphaFold3 model). Main chain atoms are represented as cylinders (only the main chain, and not the side chains, are included for clarity). Hydrogen bonds are represented as black dashed lines with lengths (determined by qtPISA [57]) indicated. Where hydrogen bonds are present in the OsHPP09-HMA/AVR-Pia crystal structure but the corresponding atoms in the AlphaFold models are separated by a distance greater than 3.5 Å, the distance (measured with PyMOL [92]) is indicated in brackets. b Position of the sidechain of AVR-PiaR43 in the crystal structure and AlphaFold models. c Position of the sidechain of OsHPP09-HMAE16 in the crystal structure and AlphaFold models. (PDF) [file ppat.1014382.s025.pdf]

# Crystal structure

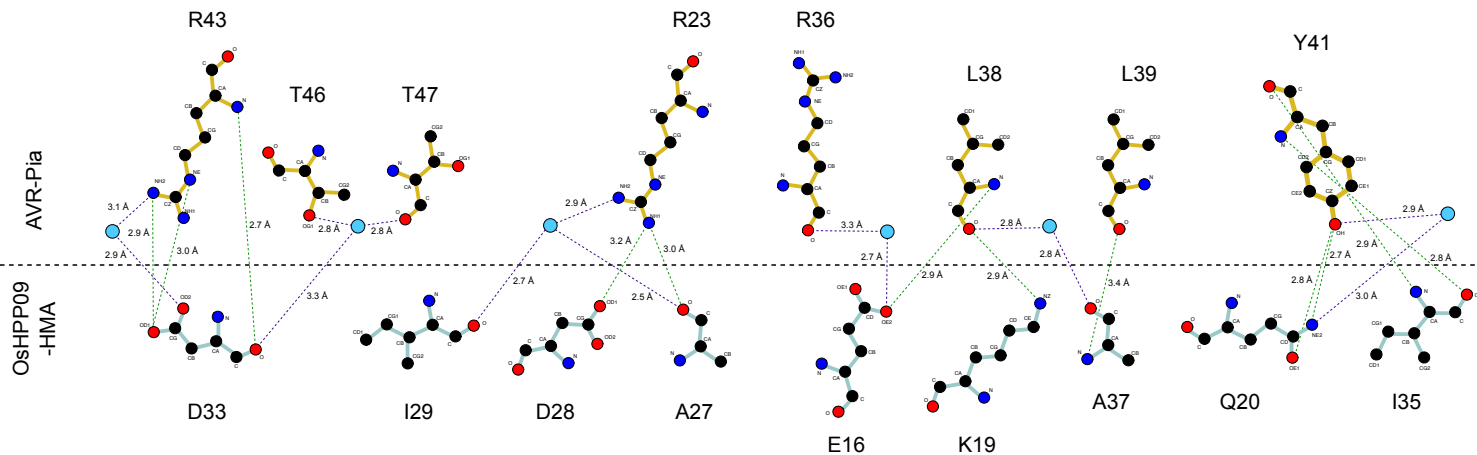

## AlphaFold2 model

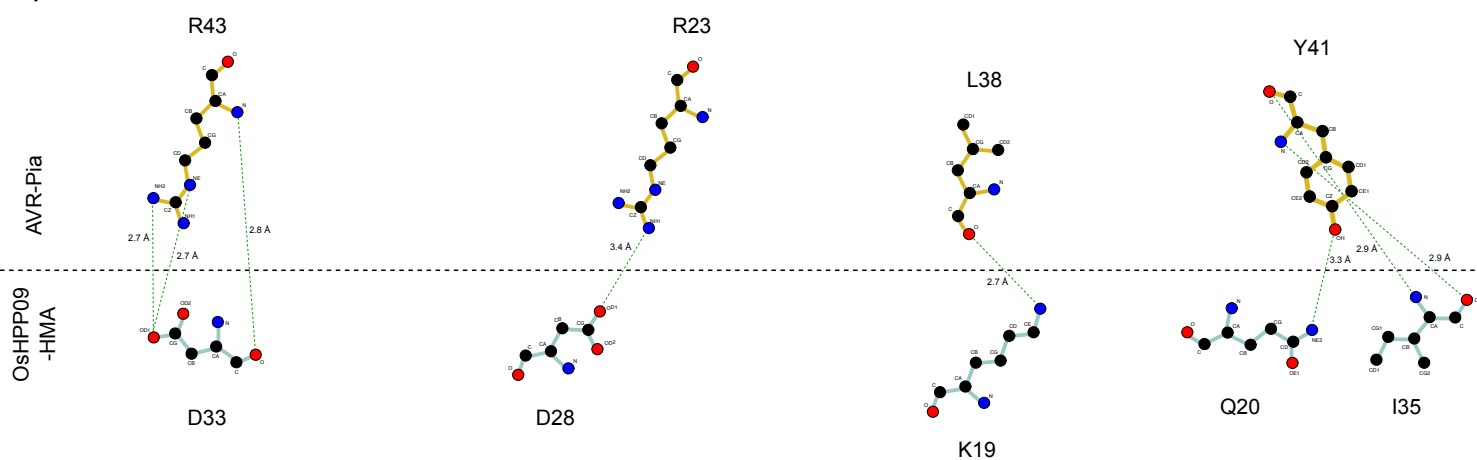

## AlphaFold3 model

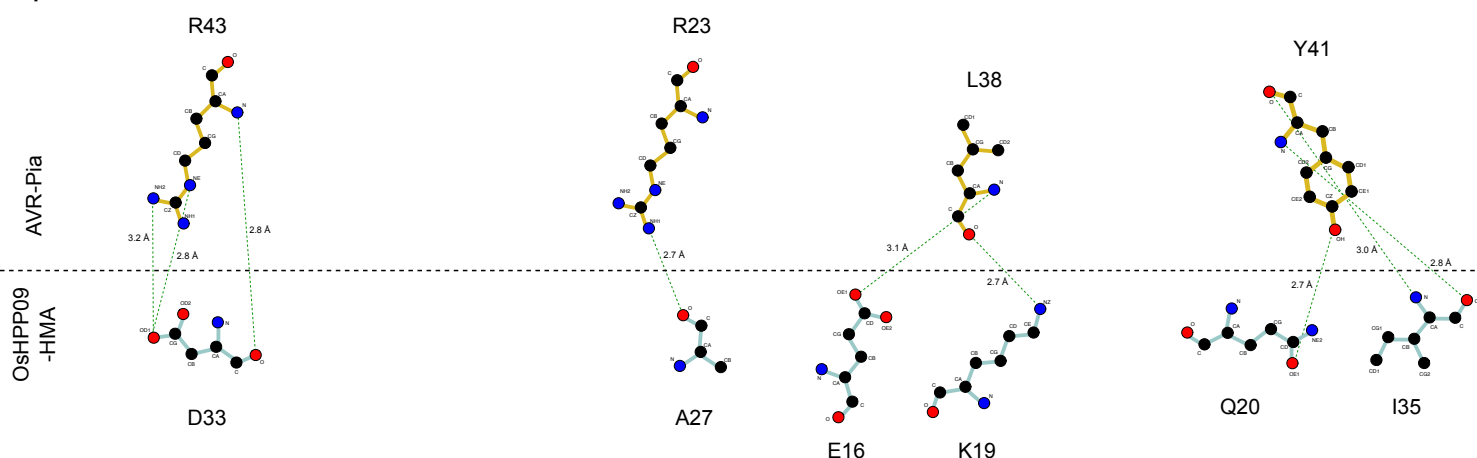

Supplement: S26 Fig — 2D protein-protein interaction diagrams adapted from the output of the DimPlot module of LigPlot+ (v2.3.1; [95,96]). Hydrogen bonds present in the qtPISA [57] output but absent from the HBPLUS output (used by LigPlot) were added manually. Intermolecular hydrogen bonds between atoms in AVR-Pia and OsHPP09-HMA are represented as green dashed lines, while hydrogen bonds involving a water molecule are represented as purple dashed lines. Water molecules are indicated by sky blue circles. Bond lengths are indicated. (PDF) [file ppat.1014382.s026.pdf]

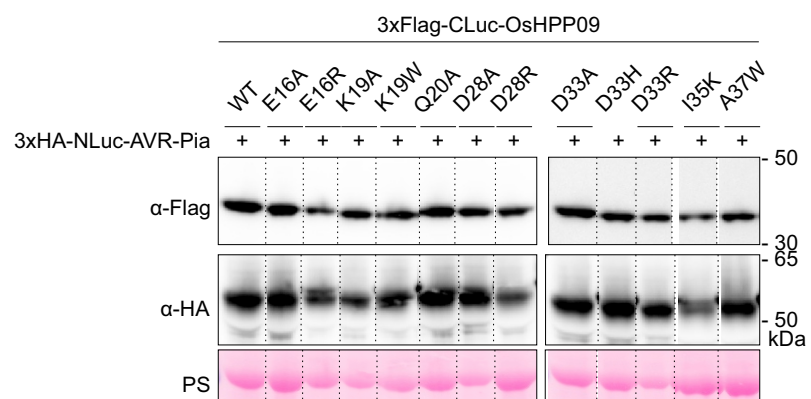

Supplement: S27 Fig — Immunoblot analysis of transiently expressed proteins in N. benthamiana, including OsHPP09 wildtype (WT) and mutant versions N-terminally tagged with a 3xFlag epitope fused to the C-terminal part of luciferase (CLuc), and AVR-Pia N-terminally tagged with 3xHA and the N-terminal part of luciferase (NLuc). This experiment corresponds to replicate 2 of Fig 7. Detection was performed using anti-Flag and anti-HA antibodies. Membrane was stripped after anti-Flag detection and re-probed with anti-HA. Protein loading is indicated by the Rubisco band visualized by Ponceau S. staining (PS). Dashed lines between bands indicate that lanes were cut from the same blot but were non-adjacent. (PDF) [file ppat.1014382.s027.pdf]

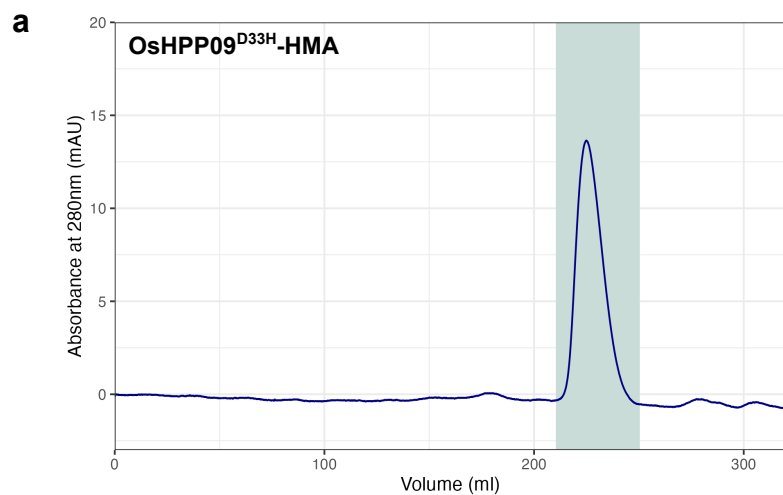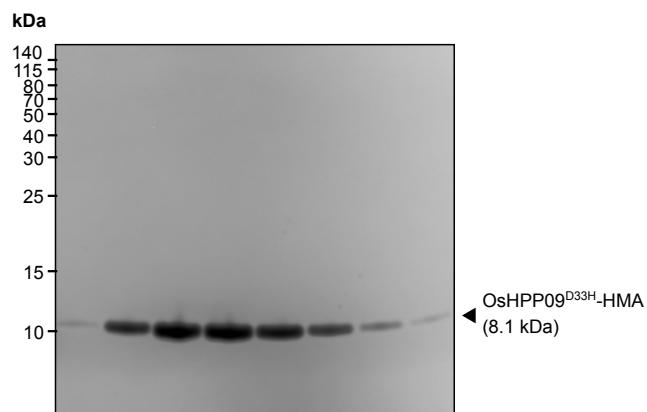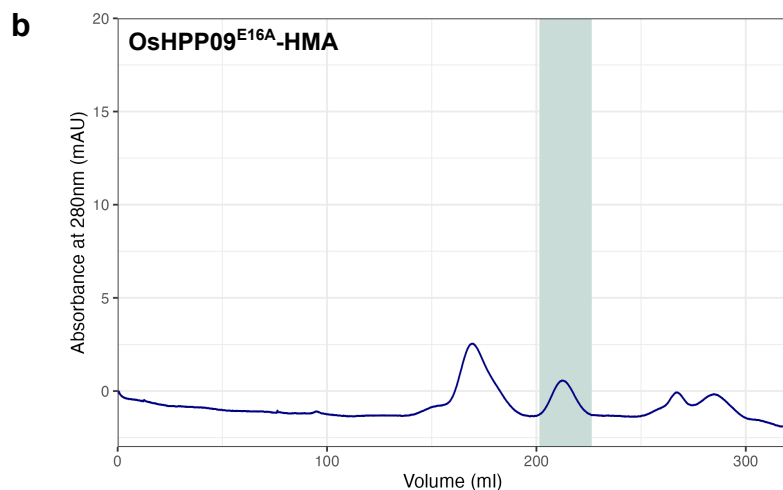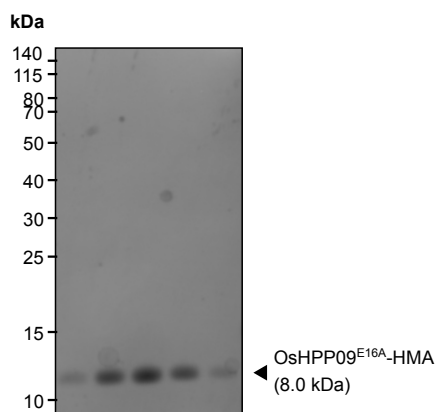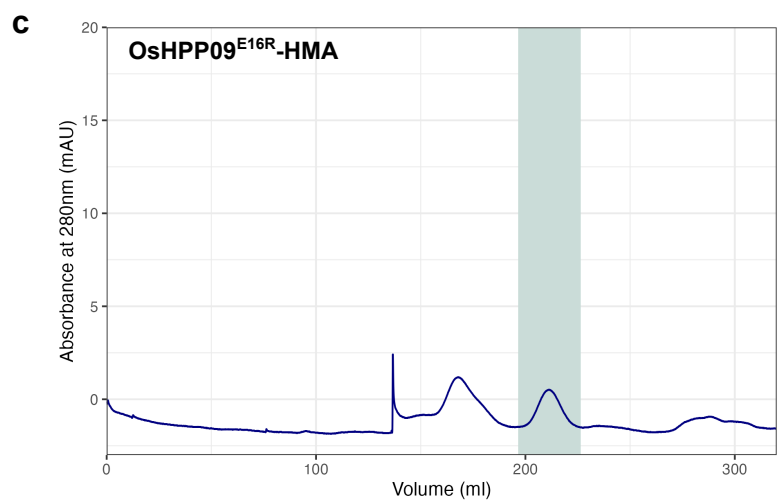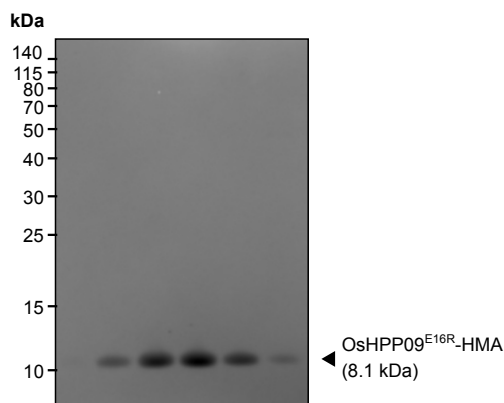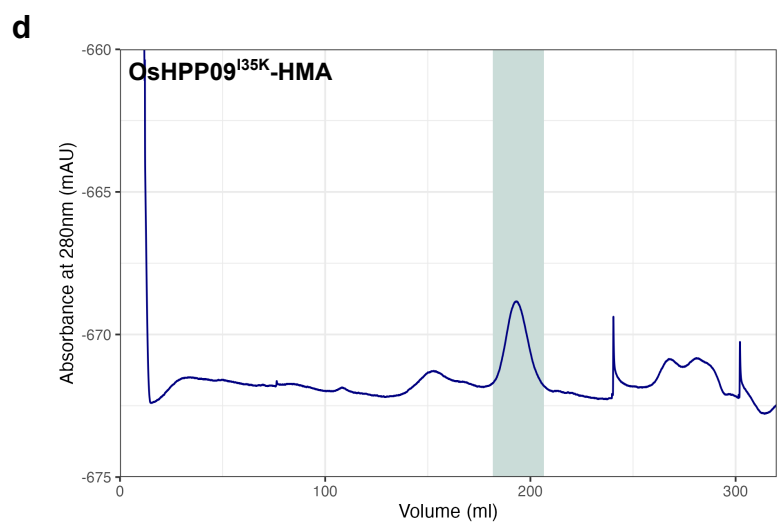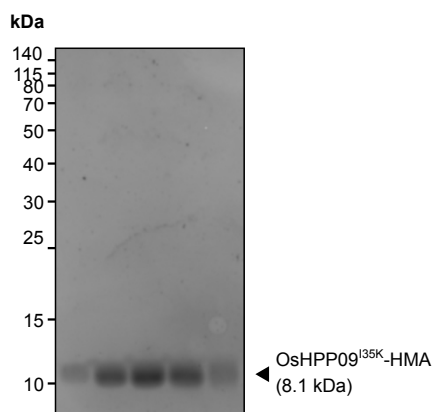

Supplement: S28 Fig — Representative size exclusion chromatography elution traces for a OsHPP09-HMAD33H, b OsHPP09-HMAE16A, c OsHPP09-HMAE16R and d OsHPP09-HMAI35K. The HMA domains absorb light at 280 nm poorly (molar extinction coefficient of 1490 M-1 cm-1) so the corresponding peaks are small. SDS-PAGE gels show fractions corresponding to the shaded areas of the trace. These fractions were pooled and concentrated to obtain the purified protein for subsequent analyses. (PDF) [file ppat.1014382.s028.pdf]

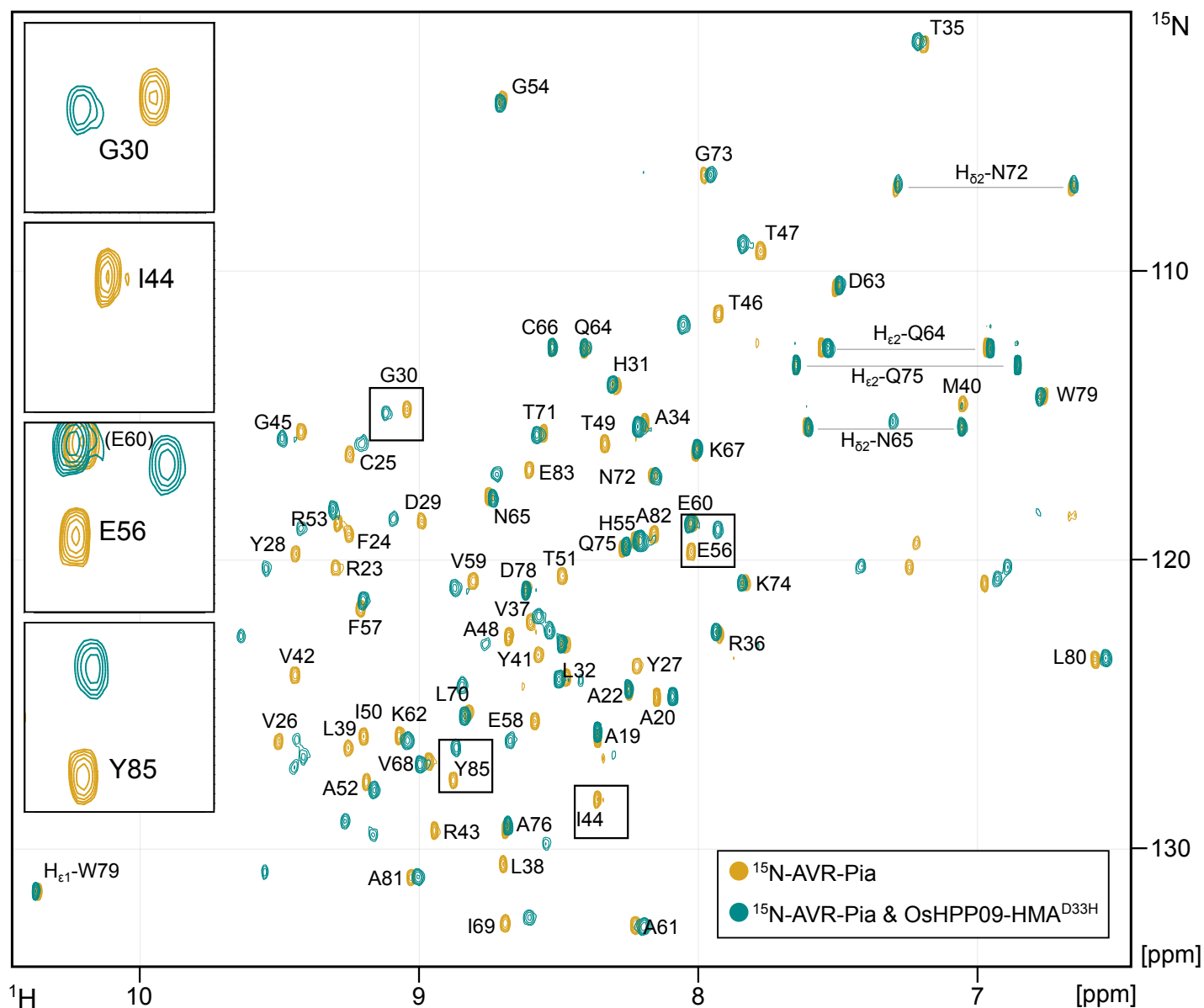

Supplement: S29 Fig — Cross-peak labels correspond to the 15N-AVR-Pia spectra (previously assigned [4]). Zoom inset panels show cross-peaks corresponding to residues G30, I44, E56 and Y85 to illustrate the chemical shift perturbations between the free (15N-AVR-Pia alone) and bound (15N-AVR-Pia with OsHPP09D33H-HMA) as presented in Fig 7. Zoomed areas are indicated by boxes on the spectra. (PDF) [file ppat.1014382.s029.pdf]

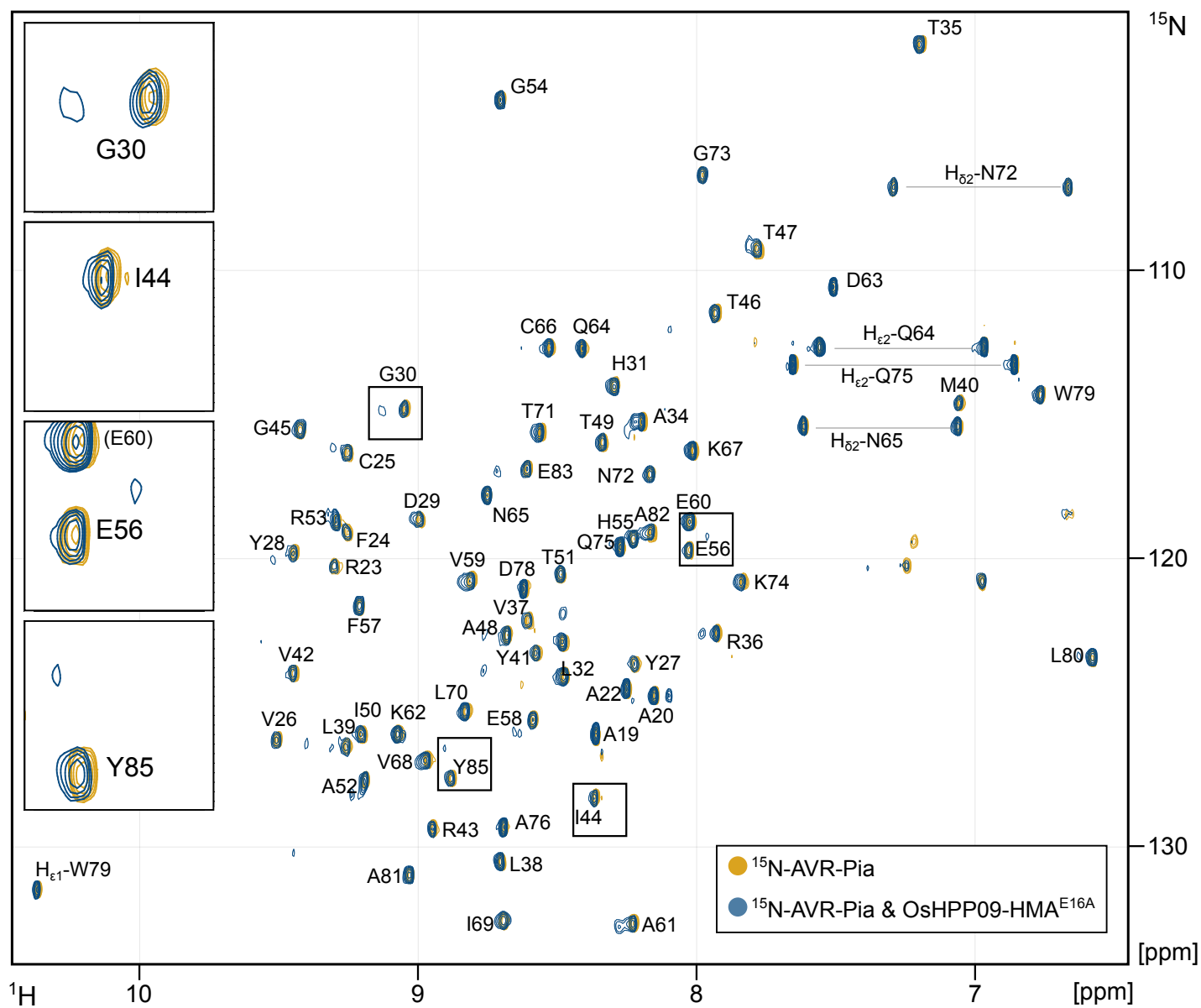

Supplement: S30 Fig — Cross-peak labels correspond to the 15N-AVR-Pia spectra (previously assigned [4]). Zoom inset panels show cross-peaks corresponding to residues G30, I44, E56 and Y85 to illustrate the chemical shift perturbations between the free (15N-AVR-Pia alone) and bound (15N-AVR-Pia with OsHPP09E16A-HMA) as presented in Fig 7. Zoomed areas are indicated by boxes on the spectra. (PDF) [file ppat.1014382.s030.pdf]

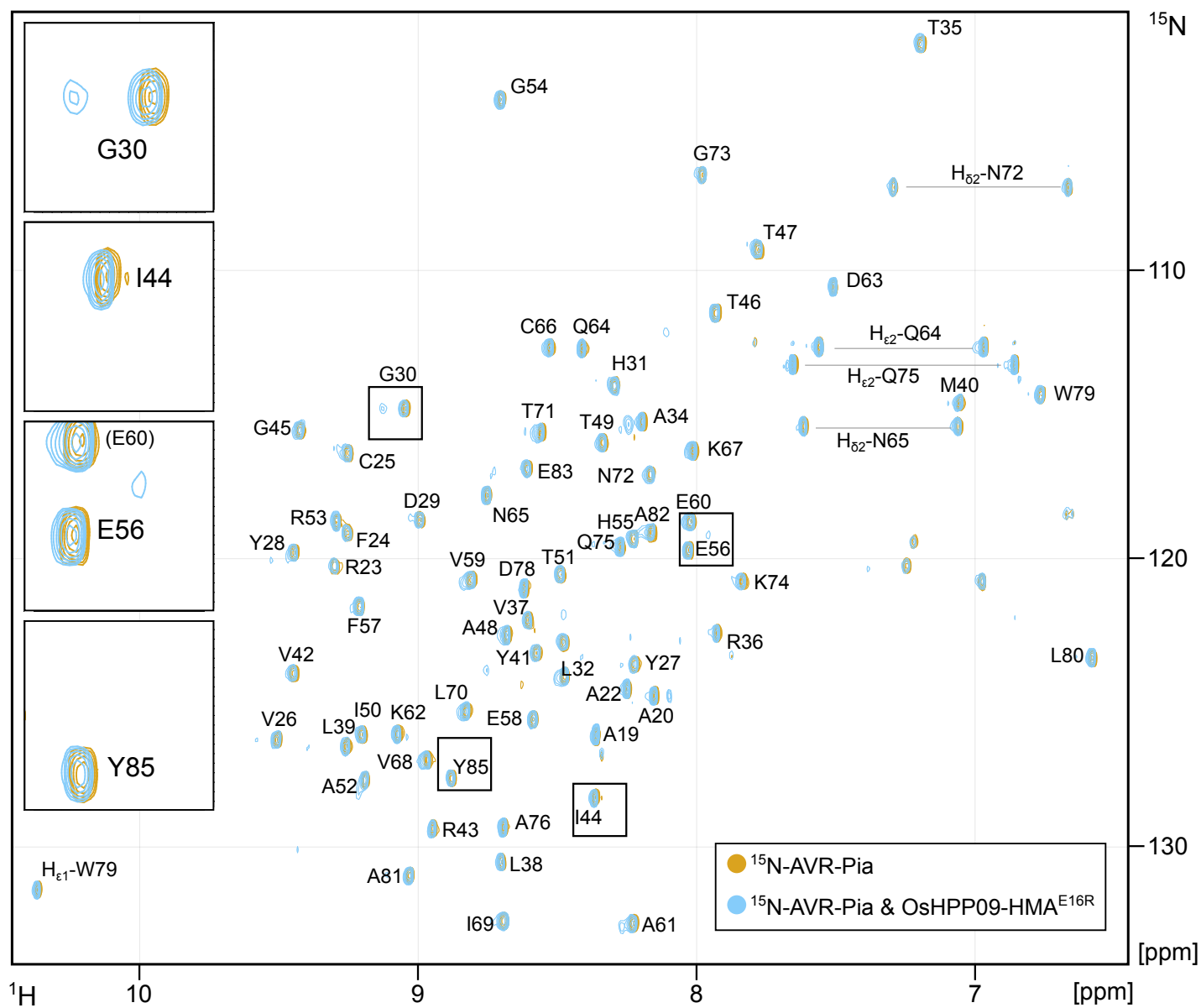

Supplement: S31 Fig — Cross-peak labels correspond to the 15N-AVR-Pia spectra (previously assigned [4]). Zoom inset panels show cross-peaks corresponding to residues G30, I44, E56 and Y85 to illustrate the chemical shift perturbations between the free (15N-AVR-Pia alone) and bound (15N-AVR-Pia with OsHPP09E16R-HMA) as presented in Fig 7. Zoomed areas are indicated by boxes on the spectra. (PDF) [file ppat.1014382.s031.pdf]

**a**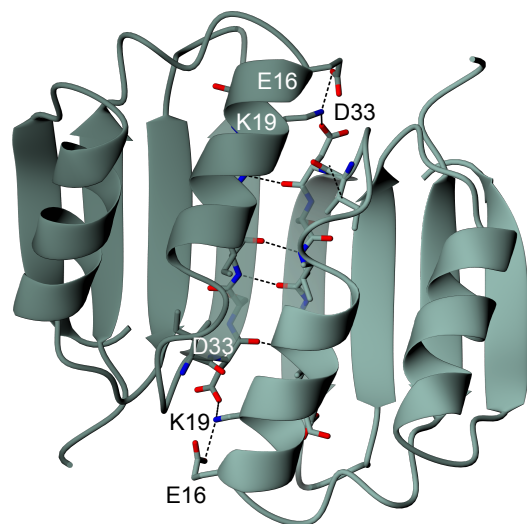**b**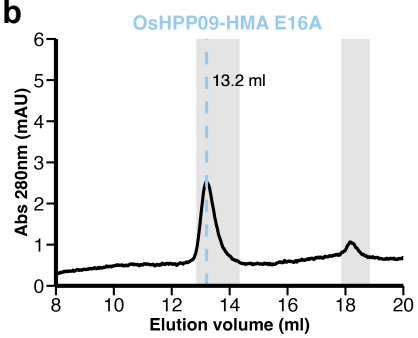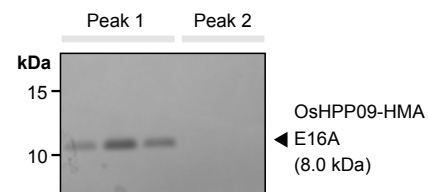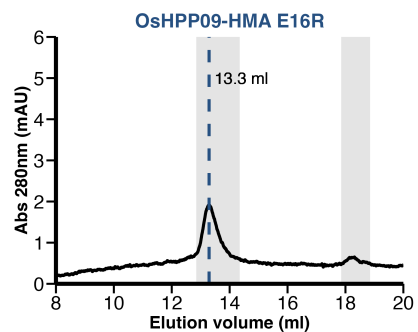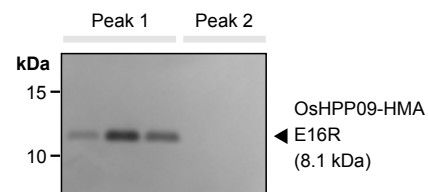

Supplement: S32 Fig — a Ribbon representation of the AlphaFold3 model (presented in S12 Fig coloured by pLDDT score) of a homodimer of OsHPP09-HMA (teal). Residues forming hydrogen bonds with the opposite protomer are represented as cylinders and hydrogen bonds are represented as black dashed lines. b Analytical gel filtration traces obtained from injection of OsHPP09-HMAE16A (top panel) or OsHPP09-HMAE16R (bottom panel). Significant peaks are indicated by coloured dashed lines with elution volume labelled. SDS-PAGE gel inserts show fractions from peak elution volumes indicated by grey shaded regions. OsHPP09-HMAE16A and OsHPP09-HMAE16R absorb light at 280 nm poorly (molar extinction coefficient: 1490 M-1 cm-1) so the corresponding peak is small. (PDF) [file ppat.1014382.s032.pdf]

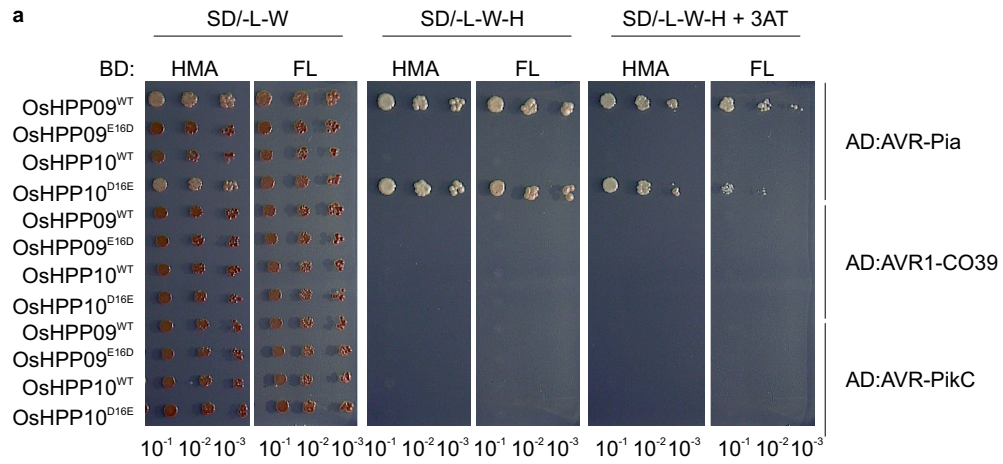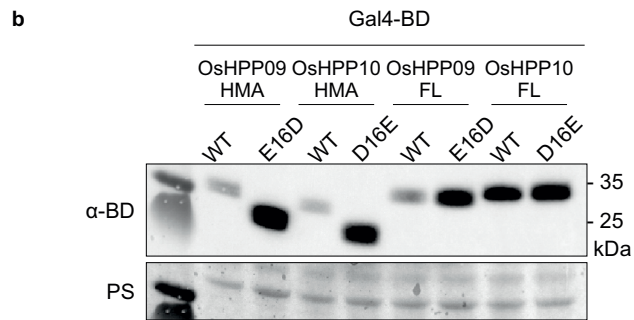

Supplement: S33 Fig — a Y2H assay testing interactions between AVR-Pia, AVR1-CO39, and AVR-PikC (all lacking signal peptide, ΔSP) and the HMA domains or full-length (FL) forms of OsHPP09 and OsHPP10. Both wild-type proteins and reciprocal mutants at position 16 were analysed, including OsHPP09E16D and OsHPP10D16E. Serial dilutions of diploid yeast were spotted onto synthetic defined (SD) media to assess growth (SD/ − LW) and protein–protein interactions (SD/ − LWH and SD/ − LWH supplemented with 0.5 mM 3-amino-1,2,4-triazole [3-AT]). Images were captured after 7 days of incubation. AD, GAL4 activation domain; BD, GAL4 DNA-binding domain. b Total protein extracts from haploid yeast cells were separated on a 10–20% Tricine SDS–PAGE gel and analysed by immunoblot using an anti-BD antibody to detect Gal4-BD fusion proteins containing wild-type or mutant HMA domains of OsHPP09 and OsHPP10, or the corresponding full-length (FL) proteins. Protein loading is indicated by Ponceau S staining (PS). (PDF) [file ppat.1014382.s033.pdf]
