# Supplementary material for: Two folds, many faces: The Magnaporthe oryzae MAX effector AVR-Pia targets novel rice HMA domain-containing proteins
Source: PLoS Pathog. 2026 Jul 13;22(7):e1014382. doi: 10.1371/journal.ppat.1014382 (PMC13395435; doi:10.1371/journal.ppat.1014382)
Supplement: S12 Fig — Ribbon representation of the crystal structures of homodimers formed by the integrated HMA domains of a OsPikp-1-HMA and b OsRGA5-HMA and c the AlphaFold3 model of a homodimer of OsHPP09-HMA coloured by pLDDT score using the classical AlphaFold colour scheme. (PDF) [file ppat.1014382.s012.pdf]

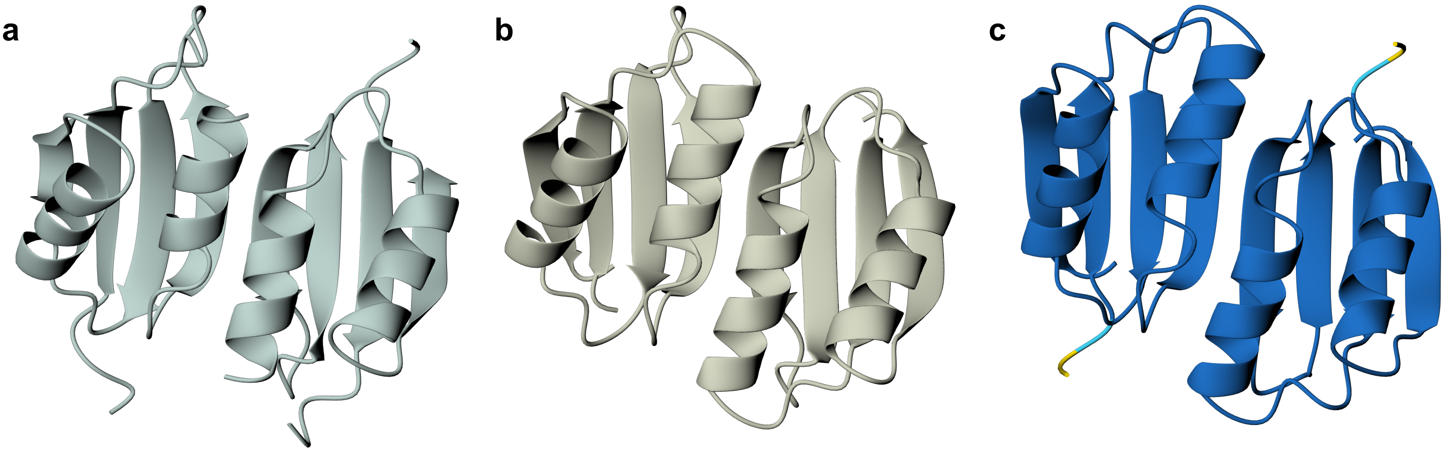

OsPikp-1-HMA homodimer
(PDB 5A6P)

OsRGA5-HMA homodimer
(PDB 5ZNE)

OsHPP09-HMA homodimer
(AlphaFold3 model)
