# Supplementary material for: Two folds, many faces: The Magnaporthe oryzae MAX effector AVR-Pia targets novel rice HMA domain-containing proteins
Source: PLoS Pathog. 2026 Jul 13;22(7):e1014382. doi: 10.1371/journal.ppat.1014382 (PMC13395435; doi:10.1371/journal.ppat.1014382)
Supplement: S35 Fig — The structures of AVR-Pia in complex with OsHPP09-HMA (PDB accession code 9RSV), AVR-PikF in complex with OsHIPP19-HMA (PDB accession code 7B1I [8]) and Pwl2 in complex with OsHIPP43-HMA (PDB accession code 8R7A [9]) are represented as gold (AVR-Pia), teal (OsHPP09-HMA), brown (Pwl2), green (OsHIPP43-HMA), pink (AVR-PikF) and grey (OsHIPP19-HMA) ribbons with relevant secondary structure elements indicated. Superposition of MAX effectors (left) and HMA domains (right) was carried out in CCP4mg [93] using secondary structure matching. (PDF) [file ppat.1014382.s035.pdf]

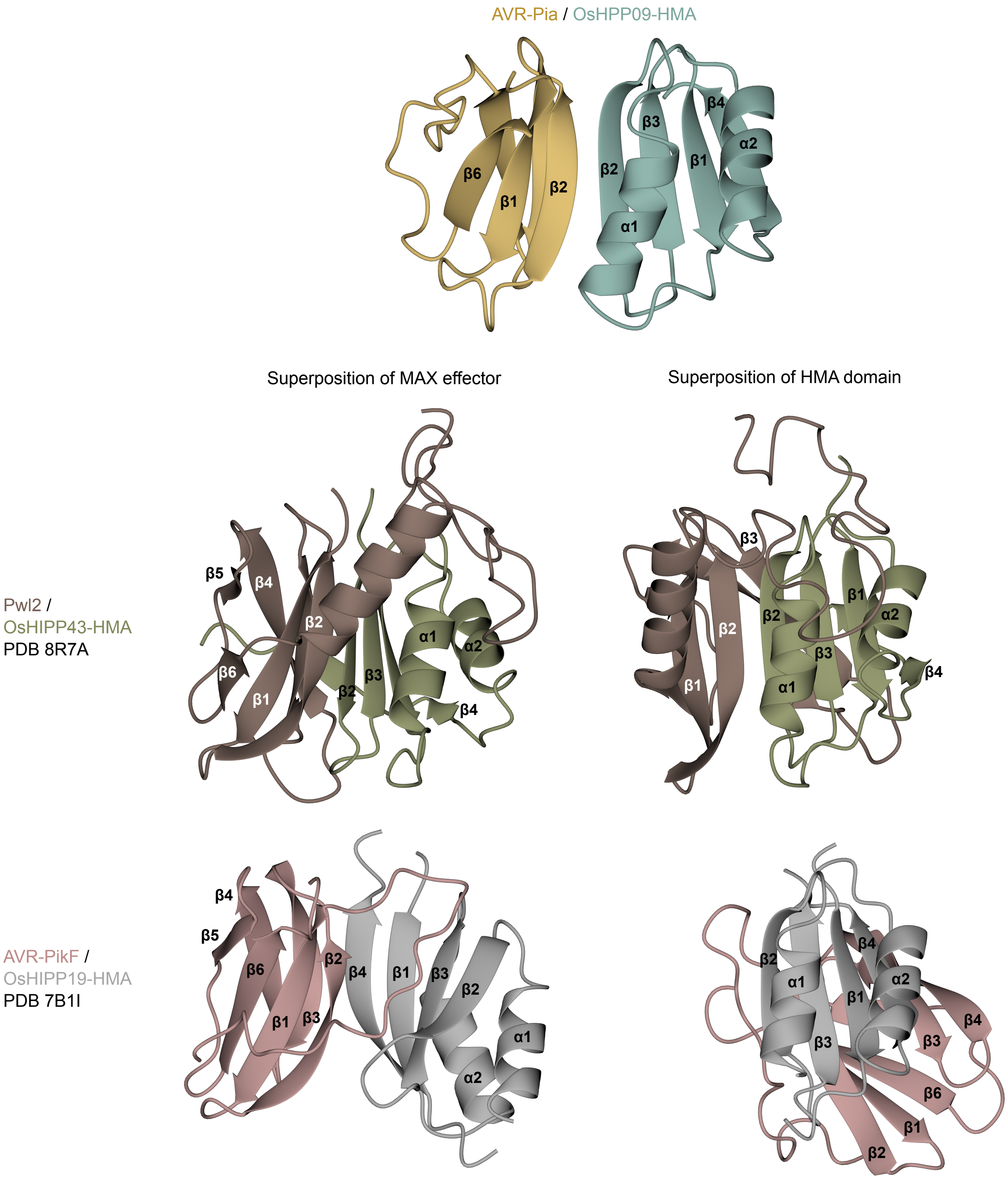

AVR-Pia / OsHPP09-HMA
Superposition of MAX effector
Superposition of HMA domain
Pwl2 /
OsHIPP43-HMA
PDB 8R7A
AVR-PikF /
OsHIPP19-HMA
PDB 7B1I
