# Supplementary material for: Two folds, many faces: The Magnaporthe oryzae MAX effector AVR-Pia targets novel rice HMA domain-containing proteins
Source: PLoS Pathog. 2026 Jul 13;22(7):e1014382. doi: 10.1371/journal.ppat.1014382 (PMC13395435; doi:10.1371/journal.ppat.1014382)
Supplement: S36 Fig — a Hydrogen bonds between residues in β2 of the effector and β2 of the HMA domain. Structures are represented as gold (AVR-Pia), teal (OsHPP09-HMA), green (OsHPP10-HMA), purple (OsHPP11-HMA) and turquoise (OsHIPP21-HMA) ribbons Main chain atoms are represented as cylinders (only the main chain, and not the side chains, are included for clarity). Hydrogen bonds are represented as black dashed lines with lengths (determined by qtPISA [57]) indicated. b-e Comparison of residues involved in forming intermolecular contacts at the effector/HMA interface in the crystal structure of AVR-Pia/OsHPP09-HMA with the corresponding residues in OsHPP10-HMA, OsHPP11-HMA and OsHIPP21-HMA. Structures are presented in ribbon representation with relevant residues shown as cylinders. Hydrogen bonds are represented as black dashed lines with lengths (determined by qtPISA [92]) indicated. Where hydrogen bonds are present in the OsHPP09-HMA/AVR-Pia crystal structure but the corresponding atoms in the AlphaFold3 models are separated by a distance greater than 3.5 Å, the distance (measured with PyMOL [92]) is indicated in brackets. (PDF) [file ppat.1014382.s036.pdf]

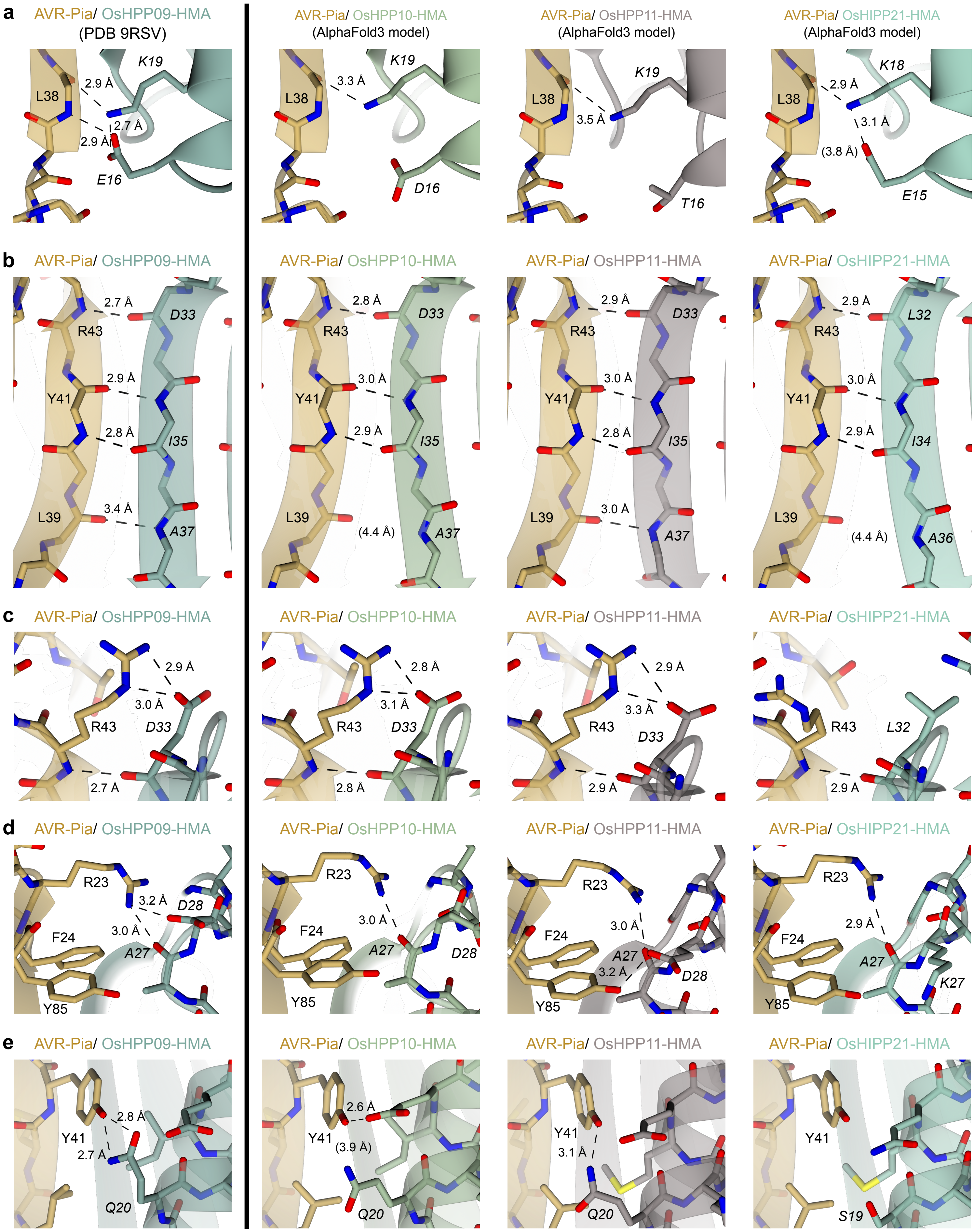

a
AVR-Pia/ OsHPP09-HMA
(PDB 9RSV)
AVR-Pia/ OsHPP10-HMA
(AlphaFold3 model)
AVR-Pia/ OsHPP11-HMA
(AlphaFold3 model)
AVR-Pia/ OsHIPP21-HMA
(AlphaFold3 model)
L38
K19
E16
D16
T16
K18
E15
2.9 Å
2.7 Å
3.3 Å
3.5 Å
3.1 Å
(3.8 Å)
b
AVR-Pia/ OsHPP09-HMA
AVR-Pia/ OsHPP10-HMA
AVR-Pia/ OsHPP11-HMA
AVR-Pia/ OsHIPP21-HMA
R43
Y41
L39
D33
I35
A37
L32
I34
A36
2.7 Å
2.8 Å
2.9 Å
3.0 Å
3.4 Å
(4.4 Å)
c
AVR-Pia/ OsHPP09-HMA
AVR-Pia/ OsHPP10-HMA
AVR-Pia/ OsHPP11-HMA
AVR-Pia/ OsHIPP21-HMA
R43
D33
L32
2.9 Å
3.0 Å
2.7 Å
2.8 Å
3.1 Å
3.3 Å
d
AVR-Pia/ OsHPP09-HMA
AVR-Pia/ OsHPP10-HMA
AVR-Pia/ OsHPP11-HMA
AVR-Pia/ OsHIPP21-HMA
R23
F24
Y85
A27
D28
K27
3.2 Å
3.0 Å
2.9 Å
e
AVR-Pia/ OsHPP09-HMA
AVR-Pia/ OsHPP10-HMA
AVR-Pia/ OsHPP11-HMA
AVR-Pia/ OsHIPP21-HMA
Y41
Q20
S19
2.8 Å
2.7 Å
2.6 Å
(3.9 Å)
3.1 Å
