# Supplementary material for: Two folds, many faces: The Magnaporthe oryzae MAX effector AVR-Pia targets novel rice HMA domain-containing proteins
Source: PLoS Pathog. 2026 Jul 13;22(7):e1014382. doi: 10.1371/journal.ppat.1014382 (PMC13395435; doi:10.1371/journal.ppat.1014382)
Supplement: S1 Table — (DOCX) [file ppat.1014382.s037.docx]

| **H(I)PP name** | **MSU/RGAP^1^ ID** | **RAP-DB/IRGSP^2^ ID** | **Amino acid sequence^3^** |
| --- | --- | --- | --- |
| OsHPP09 | LOC_Os03g02070.1 | Os03t0111400-01 | **MAQQKVVLKVPTMTDEKTKQKAIEAVADIYGIDSIAADLKDNKMTIIGDMDTVEIAKKLRKIGKIDIVSVGPA**KEEKKPEKKEEKKEEKKEEKKEEKKEEKKGKK* |
| OsHPP10 | LOC_Os10g36200.2 | [Os10t0506100-01]^4^ | **MAQQKVVLRVPTMTDDKIKQKAIEAVADIYGIDSIAADLKDNKMTIIGEMDTVAIAKKLKKIGKIDIVSVGPA**KEEKKEEKKEEKKEEKKEEKKEEKKEEKKEEKK* |
| OsHPP11 | LOC_Os04g45130.1 | [Os04t0533900-01]^5^ | **MAPQKVILKVSSMSDTKMKQKAMETVADIYGIDSIAADHKDQKMTVIGEVDTVEIAKKLKKFGKVDIISVGPA**KEEKKDDKKGDKK* |
| OsHIPP05 (Pi21) | LOC_Os04g32850.1 | Os04t0401000-01 | **MGILVILVDLQCCRCDAKIRKVLGCLEEEYCIEKVEYDVKNNRVIVRGKFDPEKLCKKIWCKAGKIIKEILIVD**VWPPPLPQPPPPCKPPPCEKPPEDCKPKPCHCCSCEKPKPKPKPCHCEKPKPCHCEKPKPCEKPPPCKPEEPPKPPPEKPPPKPECKLVPYPYPVPYPYAGQWCCPKPEPPKPPPEPPKEPEPPKPCGCSHAFVCVCKPAPPPPPPCGCSGGHGNCGCGIRPWPPQVWPPPPVCPPPPWCYTEDNANACSIM* |
| OsHIPP21 | LOC_Os09g09830.1 | Os09t0271100-02 | **MGKIKVEIKVPMTDERKKSKVMQIIAKHSGILSITADRDKDKVTIVGNENMDVTCLTMELRKQMRRTHIVIDTVTPV**DEKKEKEEKEKKEKEEKEKKEKEEKEKKKKEEEQNNPKIVCTPYYVHMVDEPSPSCCQM* |
| OsHIPP14 | LOC_Os04g39320.4 | Os04t0468600-02 | MR**KEIVIRLQSSEKGHKKAIKVAAAVSGVESVTLAGEDKNLLLVIGFGVDSNDLTEKLRRKVGHAEVVELRTV**DADELMRVAAANQYPYRYYPGAPPPAPYYGNGGYPPPHQRGGGGGGSGGGYYTPMTMATGGYYGGGGGGYPQYGQSSSYPQYGQSSSYYPPAAAATTNTHTVVHHQYANNDPDSCAIM* |
| OsHIPP19 | LOC_Os04g39350.1 | Os04t0469000-01 | **MKQKIVIKVSMPCEKSRSKAMKLVVMASGVSSVEVTGDGKDRLQVVGDGVDAACLVTCLRKKIGHAELVQVEEVKEK**KPEEKKPEEKKPEPCYCPHPCYYHHHYGGIPVAVGDQPSDPCSIM* |
| OsHIPP39 | LOC_Os03g02860.1 | Os03t0120400-01 | MSTVSSALSSFLYCCFSPTGGHRHGHRAGAYYYSSHPTSTNTYYYEGGLAGRRMGRSRPLS**LQTVELKVRMCCSGCERVVKHALMKLRGVDSVEVELEMEKVTVTGYVERQRVLKEVRRAGKKAEFW**PNPDLPLYFTSAKDYFHDEESFRPSYNYYRHGYNGDKHGHLPEPHRGADPVSNLFNDDDVNACSIM* |
| OsHIPP41 | LOC_Os03g06080.1 | Os03t0156600-01 | MGVDDIIAELRVLPAKILLKKKPKQF**QKVEVKVRMDCEGCERKVRKAVEEMKGVSSVEVDAKQNKVTVTGYVEQEEVVGRLRRRAGKKAEPW**PYVPYDVVPHPYAPGAYDKKAPPGYVRNALADPDAAPLARATEEEEKLASAFSDENPNSCAVM* |
| OsHIPP43 | LOC_Os01g32330.1 | Os01t0507700-01 | MGVLDSLSDMCSLTETKEALKLRKKRP**LQTVNIKVKMDCEGCERRVKNAVKSMRGVTSVAVNPKQSRCTVTGYVEASKVLERVKSTGKAAEMW**PYVPYTMTTYPYVGGAYDKKAPAGFVRGNPAAMADPSAPEVRYMTMFSDENVDSCSIM* |

^1^ https://rice.uga.edu/

^2^ https://rapdb.dna.affrc.go.jp/

^3^ HMA domain is in **bold**. MxCxxC motif (in the HMA domain) is **bold underlined**. CααX motif is underlined.

^4^ RAP-DB annotation matches the MSU .1 annotation. The MSU .2 annotation is supported by RNAseq data. No RAP-DB annotation matches MSU.2.

^5^ RAP-DB annotation is missing the start codon and first exon.
