# Supplementary material for: Two folds, many faces: The Magnaporthe oryzae MAX effector AVR-Pia targets novel rice HMA domain-containing proteins
Source: PLoS Pathog. 2026 Jul 13;22(7):e1014382. doi: 10.1371/journal.ppat.1014382 (PMC13395435; doi:10.1371/journal.ppat.1014382)
Supplement: S2 Table — Results of a Y2H screen using BD:AVR-Pia as bait and a cDNA library from the rice cultivar CO39. The number of positive clones with insert sequences (cloned in frame with Gal4 AD) corresponding to the designated proteins are shown. * Potential false positives found in >50% of the Y2H screens performed in the lab using various bait proteins. (DOCX) [file ppat.1014382.s038.docx]

| **Identified cDNA (MSU/RGAP ID)** | **Encoded rice protein** | **Number of clones** | **Comment** |
| --- | --- | --- | --- |
| LOC_Os04g48310.1 | RING-H2 finger protein, putative, expressed | 117 | *** |
| LOC_Os01g60350.1 | expressed protein | 11 | *** |
| LOC_Os02g22130.1 | expressed protein | 8 | *** |
| LOC_Os01g13760.1 | dnaJ domain containing protein, expressed | 3 |  |
| LOC_Os01g48280.1 | ubiquitin-conjugating enzyme, putative, expressed | 3 |  |
| LOC_Os03g16860.2 | DnaK family protein, putative, expressed | 3 |  |
| LOC_Os04g37619.1 | zeaxanthin epoxidase, chloroplast precursor, putative, expressed | 3 |  |
| LOC_Os05g50710.1 | late embryogenesis abundant protein, putative, expressed | 3 | *** |
| LOC_Os07g40580.1 | eukaryotic translation initiation factor 5A, putative, expressed | 3 |  |
| LOC_Os12g14070.1 | DnaK family protein, putative, expressed | 3 |  |
| LOC_Os01g05060.1 | mitochondrial glycoprotein, putative, expressed | 2 |  |
| LOC_Os01g06660.1 | thiamine pyrophosphate enzyme, C-terminal TPP binding domain containing protein, expressed | 2 |  |
| LOC_Os02g52290.1 | peptidyl-prolyl cis-trans isomerase, FKBP-type, putative, expressed | 2 | *** |
| LOC_Os03g16860.1 | DnaK family protein, putative, expressed | 2 |  |
| LOC_Os05g03630.1 | dnaJ domain containing protein, expressed | 2 |  |
| LOC_Os05g38370.1 | peptidyl-prolyl cis-trans isomerase, FKBP-type, putative, expressed | 2 |  |
| LOC_Os06g04290.1 | S10/S20 domain containing ribosomal protein, putative, expressed | 2 |  |
| LOC_Os09g24210.1 | expressed protein | 2 |  |
| LOC_Os01g13470.1 | KH domain containing protein, putative, expressed | 1 |  |
| LOC_Os01g62610.1 | peptidyl-prolyl cis-trans isomerase, FKBP-type, putative, expressed | 1 |  |
| LOC_Os02g02410.1 | DnaK family protein, putative, expressed | 1 |  |
| LOC_Os02g08380.1 | CR084 protein, putative, expressed | 1 |  |
| LOC_Os02g49150.3 | RNA polymerase Rpb4, putative, expressed | 1 |  |
| LOC_Os02g57305.1 | disease resistance protein, putative, expressed | 1 |  |
| LOC_Os03g38640.1 | expressed protein | 1 |  |
| LOC_Os03g61630.2 | WD domain, G-beta repeat domain containing protein, expressed | 1 |  |
| LOC_Os04g01780.1 | uncharacterized ACR, COG1399 family protein, expressed | 1 |  |
| LOC_Os04g39560.3 | expressed protein | 1 |  |
| LOC_Os05g23740.1 | DnaK family protein, putative, expressed | 1 |  |
| LOC_Os07g32380.1 | protein phosphatase 2C, putative, expressed | 1 |  |
| LOC_Os07g41810.4 | stress responsive A/B Barrel domain containing protein, expressed | 1 |  |
| LOC_Os09g09830.1 | heavy-metal-associated domain-containing protein, putative, expressed | 1 | OsHIPP21 |
| LOC_Os09g36770.2 | NTMC2Type1.2 protein, putative, expressed | 1 |  |
| LOC_Os10g08930.1 | S10/S20 domain containing ribosomal protein, putative, expressed | 1 |  |
| LOC_Os11g47760.4 | DnaK family protein, putative, expressed | 1 |  |
| LOC_Os12g38170.1 | osmotin, putative, expressed | 1 |  |
