# Supplementary material for: Two folds, many faces: The Magnaporthe oryzae MAX effector AVR-Pia targets novel rice HMA domain-containing proteins
Source: PLoS Pathog. 2026 Jul 13;22(7):e1014382. doi: 10.1371/journal.ppat.1014382 (PMC13395435; doi:10.1371/journal.ppat.1014382)
Supplement: S3 Table — (DOCX) [file ppat.1014382.s039.docx]

|  | | *K*_D_ (M) | ∆H  (kcal/mol) | ∆G (kcal/mol) | -T∆S (kcal/mol) | N (sites) |
| --- | --- | --- | --- | --- | --- | --- |
| **OsHPP09-HMA & AVR-Pia** | **1** | 1.15 x 10^-7^ ± 1.42 x 10^-8^ | -13.7 ± 0.175 | -9.47 | 4.27 | 0.972 ± 0.0052 |
|  | **2** | 1.25 x 10^-7^ ± 3.02 x 10^-8^ | -11.2 ± 0.277 | -9.42 | 1.79 | 0.859 ± 0.0098 |
|  | **3** | 1.85 x 10^-7^ ± 5.09 x 10^-8^ | -13.3 ± 0.436 | -9.19 | 4.08 | 0.528 ± 0.01 |
| **OsHPP10-HMA & AVR-Pia** | **1** | 5.26 x 10^-6^ ± 1.47 x 10^-6^ | -3.46 ± 0.292 | -7.2 | -3.74 | 1.18 ± 0.055 |
|  | **2** | 6.51 x 10^-6^ ± 2.04 x 10^-6^ | -3.47 ± 0.345 | -7.08 | -3.61 | 1.17 ± 0.066 |
|  | **3** | 7.47 x 10^-6^ ± 3.69 x 10^-6^ | -3.18 ± 0.644 | -7 | -3.81 | 1.4 ± 0.177 |
| **OsHPP11-HMA & AVR-Pia** | **1** | 7.72 x 10^-7^ ± 2.33 x 10^-7^ | -5.7 ± 0.263 | -8.34 | -2.64 | 0.697 ± 0.018 |
|  | **2** | 9.25 x 10^-7^ ± 1.78 x 10^-7^ | -5.63 ± 0.182 | -8.23 | -2.6 | 0.727 ± 0.012 |
|  | **3** | 9.29 x 10^-7^ ± 1.83 x 10^-7^ | -6.28 ± 0.192 | -8.23 | -1.95 | 0.749 ± 0.013 |
| **OsHIPP21-HMA & AVR-Pia** | **1** | 1.29 x 10^-6^ ± 1.70 x 10^-7^ | -5.66 ± 0.145 | -8.03 | -2.37 | 0.576 ± 0.0083 |
|  | **2** | 1.97 x 10^-6^ ± 4.81 x 10^-7^ | -4.84 ± 0.3 | -7.79 | -2.95 | 0.533 ± 0.017 |
|  | **3** | 2.33 x 10^-6^ ± 3.83 x 10^-7^ | -7.04 ± 0.271 | -7.68 | -0.642 | 0.789 ± 0.016 |
