# Supplementary material for: Two folds, many faces: The Magnaporthe oryzae MAX effector AVR-Pia targets novel rice HMA domain-containing proteins
Source: PLoS Pathog. 2026 Jul 13;22(7):e1014382. doi: 10.1371/journal.ppat.1014382 (PMC13395435; doi:10.1371/journal.ppat.1014382)
Supplement: S4 Table — (DOCX) [file ppat.1014382.s040.docx]

|  | **OsHPP09-HMA / AVR-Pia** |
| --- | --- |
| **Data collection statistics** |  |
| Wavelength (Å) | 0.9655 |
| Space group | *P* 6_1_ 2 2 |
| Cell dimensions: |  |
| *a*, *b*, *c* (Å) | 93.67, 93.67, 72.87 |
| α, β, γ (°) | 90.00, 90.00, 120.00 |
| Resolution (Å)* | 46.83-1.65 (1.68-1.65) |
| *R_meas_ (%)^#^* | 4.5 (105.6) |
| *R*_merge_ (%)^#^ | 4.0 (96.2) |
| *I*/σ*I*^#^ | 19.2 (1.9) |
| Completeness (%)^#^ | 99.8 (100.0) |
| Unique reflections^#^ | 23168 (1125) |
| Redundancy^#^ | 5.6 (5.9) |
| CC^(1/2)^ (%)^#^ | 99.9 (83.7) |
| **Refinement statistics** |  |
| Resolution (Å) | 46.88-1.65 (1.69-1.65) |
| *R*_work_/*R*_free_ (%)^^^ | 17.6/20.2 (29.1/30.4) |
| No. atoms (Protein) | 2125 |
| No. atoms (Ligand/ion) | 10 |
| No. atoms (Water) | 194 |
| B-factors (Protein) | 35.8 |
| B-factors (Ligand/ion) | 33.5 |
| B-factors (Water) | 43.7 |
| R.m.s. deviations:^^^ |  |
| Bond lengths (Å) | 0.0145 |
| Bond angles (º) | 2.243 |
| Ramachandran plot (%): ** |  |
| Favoured | 99.2 |
| Allowed | 0.8 |
| Outliers | 0.0 |
| Rotamer outliers (%) ** | 2.65 |
| Clashscore ** | 1.39 |
| MolProbity Score ** | 1.19 |

*The highest resolution shell is shown in parenthesis.

^#^As calculated by Aimless, ^^^As calculated by Refmac5, **As calculated by MolProbity
