# Supplementary material for: Two folds, many faces: The Magnaporthe oryzae MAX effector AVR-Pia targets novel rice HMA domain-containing proteins
Source: PLoS Pathog. 2026 Jul 13;22(7):e1014382. doi: 10.1371/journal.ppat.1014382 (PMC13395435; doi:10.1371/journal.ppat.1014382)
Supplement: S5 Table — Interface analysis was performed using qtPISA [57]. (DOCX) [file ppat.1014382.s041.docx]

| **Complex** | | **HMA domain** | OsHPP09 | OsHIPP19 | OsHIPP43 | Pikp-1 | RGA5 |
| --- | --- | --- | --- | --- | --- | --- | --- |
|  |  | **MAX effector** | AVR-Pia | AVR-PikF | HMA PWL2 | AVR-Pia | AVR1-CO39 |
|  | **PDB accession code** | | 9RSV | 7B1I | 8R7A | 6Q76 | 5ZNG |
| **Interface parameter** | **Interface area (Å^2^)** | | 521.6 | 1044.3 | 1965.2 | 460.7 | 492.8 |
|  | **Solvation energy (kcal/mol)** | | -1.9 | -0.1 | -7.6 | -4.7 | -4.6 |
|  | **Binding energy (kcal/mol)** | | -7.5 | -11.3 | -27.8 | -8.6 | -7.3 |
|  | **Hydrophobic P-value** | | 0.5066 | 0.6998 | 0.3771 | 0.3701 | 0.3317 |
|  | **Hydrogen bonds** | | 10 | 17 | 38 | 7 | 6 |
|  | **Salt bridges** | | 3 | 10 | 9 | 2 | 0 |
|  | **Disulphide bonds** | | 0 | 0 | 0 | 0 | 0 |
