# Supplementary material for: Two folds, many faces: The Magnaporthe oryzae MAX effector AVR-Pia targets novel rice HMA domain-containing proteins
Source: PLoS Pathog. 2026 Jul 13;22(7):e1014382. doi: 10.1371/journal.ppat.1014382 (PMC13395435; doi:10.1371/journal.ppat.1014382)
Supplement: S34 Fig — a Split luciferase complementation assays assessing interaction between AVR-Pia (ΔSP; fused to the N-terminal luciferase fragment, 3 × HA:NLuc:AVR-Pia) and full-length H(I)PP proteins carrying mutations at the position equivalent to OsHPP09 E16 (fused to the C-terminal luciferase fragment, 3 × Flag:CLuc:OsH(I)PPs). Co-expression of AVR-Pia with wild-type OsH(I)PPs served as a positive control, whereas co-expression with OsHIPP43 served as a negative control. Leaves infiltrated with P19 alone were used as background (BG) control. Box plots display the median (line), mean (cross), and interquartile range (box limits); whiskers extend to the most extreme values within 1.5 × the interquartile range, with outliers shown individually. Three independent experiments were performed, each with n = 6 per combination. Data points are shown with symbols corresponding to each replicate. Different letters indicate statistically significant differences based on pairwise Wilcoxon tests (α = 0.05). b Immunoblot analysis of transiently expressed proteins in N. benthamiana. Full-length OsH(I)PPs were fused to a C-terminal 3 × Flag tag and the C-terminal luciferase fragment (CLuc), while AVR-Pia was fused N-terminally to 3 × HA and the N-terminal luciferase fragment (NLuc). Shown is replicate 3. Proteins were detected using anti-Flag and anti-HA antibodies; the membrane was stripped after anti-Flag detection and reprobed with anti-HA. Protein loading is indicated by the Rubisco band visualized by Ponceau S staining (PS). (PDF) [file ppat.1014382.s034.pdf]

a

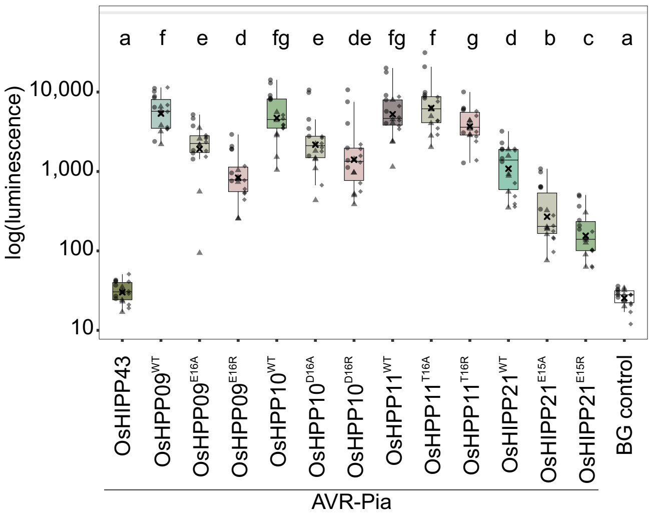
Rep. 1 2 3
a f e d fg e de fg f g d b c a
10,000
1,000
100
10
log(luminescence)
OsHIPP43
OsHPP09WT
OsHPP09E16A
OsHPP09E16R
OsHPP10WT
OsHPP10D16A
OsHPP10D16R
OsHPP11WT
OsHPP11T16A
OsHPP11T16R
OsHIPP21WT
OsHIPP21E15A
OsHIPP21E15R
BG control
AVR-Pia

b

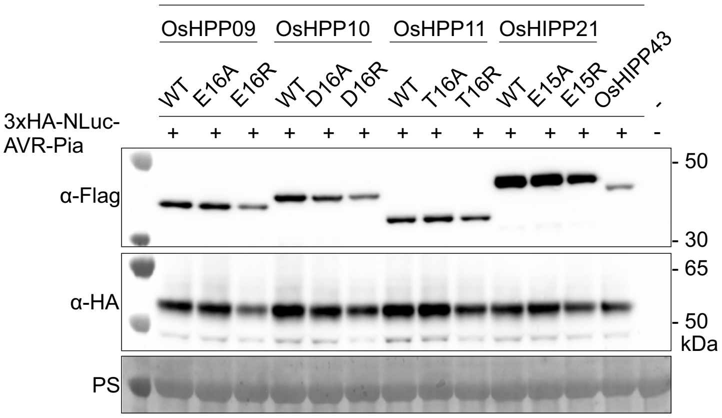
3xFlag-CLuc-
OsHPP09 OsHPP10 OsHPP11 OsHIPP21
WT E16A E16R WT D16A D16R WT T16A T16R WT E15A E15R OsHIPP43 -
3xHA-NLuc-
AVR-Pia
+ + + + + + + + + + + + + -
α-Flag
α-HA
PS
50
30
65
50
kDa
