# Supplementary material for: Two folds, many faces: The Magnaporthe oryzae MAX effector AVR-Pia targets novel rice HMA domain-containing proteins
Source: PLoS Pathog. 2026 Jul 13;22(7):e1014382. doi: 10.1371/journal.ppat.1014382 (PMC13395435; doi:10.1371/journal.ppat.1014382)
Supplement: Attachment — Submitted filename: 2026-04-30_response-to-reviewers_v7.pdf [file ppat.1014382.s044.pdf]

Dear Dr Krasileva,

Thank you for the opportunity to submit a revised version of our manuscript "Two folds, many faces: the *Magnaporthe oryzae* MAX effector AVR-Pia targets novel rice HMA domain-containing proteins" by Maidment, Saile et al. (PPATHOGENS-D-25-02941) for consideration by PLOS Pathogens.

We appreciate the considered, insightful and constructive feedback from the reviewers and have acted on their comments and suggestions, which we believe has significantly improved the manuscript.

Please find below, a point-by-point response to the reviewers' comments. The changes we have made are highlighted within the manuscript and all line numbers refer to the revised manuscript file with tracked changes.

Sincerely,

Stella Cesari and Karine de Guillen

MAJOR ISSUES

Reviewer #1

A central opportunity for this manuscript is to clarify whether AVR-Pia engages diverse HMA domains (host targets and/or integrated decoys) through a conserved binding mode. The current work convincingly solves one interaction (e.g., AVR-Pia–OsHPP09-HMA) and could compare that with the interaction with the integrated HMA of RGA5. Despite this it stops short of establishing whether the identified interface is representative across interactors like 10, 11 and 21. Is E16 conserved within any of the other OsHPP proteins, how would mutation of this, or the equivalent residue, impact these interactions?

We thank the reviewer for this suggestion, and have performed additional experiments to address this question, specifically:

1. NMR titration of ^{15}N-labelled AVR-Pia with the purified HMA domains of OsHPP10, OsHPP11, and OsHIPP21 (**Supplementary Fig. 23, lines 355-362**). The results demonstrated that, consistent with the AlphaFold models, the same interface of AVR-Pia is involved in interaction with all four HMA domains.

   "^{1}H-^{15}N-HSQC NMR 2D spectra were also recorded during NMR titration of ^{15}N-labelled AVR-Pia with unlabelled OsHPP10-HMA, OsHPP11-HMA or OsHIPP21-HMA (**Supplementary Fig. 23**). In these cases, a decrease in intensity was observed for certain peaks, indicating a rapid exchange regime. This is typical for interactions with weaker affinity (*K*D in the micromolar range), and is consistent with the ITC results. Notably, in all three cases, significant changes in intensity were detected for peaks corresponding to residues in the β1, β2 and β3 regions, whereas peaks corresponding to residues on the opposite face remained unaffected. This indicates that similar residues in AVR-Pia are involved in binding to all four HMA domains. "

2. Alanine and arginine substitution of the residue corresponding to OsHPP09^{E16} in OsHPP10 (D16A, D16R), OsHPP11 (T16A, T16R), and OsHIPP21 (E15A, E15R), and assessment of in planta interaction with AVR-Pia by split-luciferase. This experiment showed that mutation of the equivalent residue in OsHPP10 and OsHIPP21 significantly reduced

interaction with AVR-Pia. However, equivalent substitutions in OsHPP11 had no significant effect (**Supplementary Fig. 34, lines 514-522**).

"To establish the importance of the residue corresponding to OsHPP09^{E16} in OsHPP10 (D16), OsHPP11 (T16) and OsHIPP21 (E15) for the interaction of the H(I)PPs with AVR-Pia, we mutated each residue to alanine (A) and arginine (R) and tested for an interaction with AVR-Pia in planta using split luciferase complementation assays. Consistent with the effect observed for the E16A and E16R substitutions in OsHPP09, mutation of the equivalent residue in OsHPP10 and OsHIPP21 significantly reduced their interaction with AVR-Pia. In contrast, the T16A and T16R substitutions in OsHPP11 did not significantly impact AVR-Pia binding (**Supplementary Fig. 34a**). Immunoblot analysis confirmed that all proteins were expressed at detectable levels (**Supplementary Fig. 34b**)."

3. Glutamate substitution of the OsHPP10^{D16} and assessment of interaction with AVR-Pia by Y2H. The results showed that the D16E substitution in OsHPP10 restores interaction with AVR-Pia (**Supplementary Fig. 33, lines 504-513**).

   "Despite differing by an order of magnitude in their affinity for AVR-Pia binding, OsHPP10-HMA and OsHPP09-HMA vary in just six amino acid positions. Intriguingly, the only residue of the six that is located at the interface with AVR-Pia is OsHPP09^{E16}/OsHPP10^{D16} (**Supplementary Fig. 5**). To test whether this polymorphism is responsible for the observed difference in strength of binding to AVR-Pia, we generated the reciprocal mutants OsHPP09^{E16D} and OsHPP10^{D16E} and tested for interaction with AVR-Pia in pairwise Y2H assays. Strong yeast growth on interaction-selective media was observed when AVR-Pia was paired with WT OsHPP09 (HMA or FL), or OsHPP10^{D16E} (HMA or FL). By contrast, no yeast growth was observed for OsHPP09^{E16D} nor OsHPP10 (**Supplementary Fig. 33a**). Expression of all proteins was confirmed by immunoblot analyses (**Supplementary Fig. 33b**)."

The authors make the observation that AF2 and AF3 are unable to distinguish the true/false interactors, an important result. Despite this it appears to me based on the figures provided that AF does predict the interface itself correctly. In fact, almost all the models predict the same binding mode. I don't think this is mentioned in the current manuscript, I do believe further comparisons between the solved structure and AF prediction should be included.

We thank the reviewer for this suggestion. We have included the following sentence in the results section (**lines 380-383**):

"Globally, the AVR-Pia/OsHPP09-HMA complex resembles the previously determined structures of AVR-Pia/OsPikp-1-HMA (PDB 6Q76) [48] and AVR1-CO39/OsRGA5-HMA (PDB 5ZNG) [12] and the AlphaFold models of AVR-Pia/OsRGA5-HMA and AVR-Pia/OsH(I)PP-HMA complexes (**Supplementary Fig. 24**)."

To illustrate the similarities and differences between the crystal structure and AlphaFold models of the AVR-Pia/OsHPP09-HMA complex, we have added to the results section the following paragraph (**lines 420-432**) and additional figures (**Supplementary Fig. 25, 26**):

"Comparisons revealed that the crystal structure of OsHPP09-HMA/AVR-Pia was highly similar to the AlphaFold2 and AlphaFold3 models (global RMSD of 0.514 Å and 0.606 Å, respectively). Of the 12 hydrogen bonds and salt bridges in the experimental structure, 8 were present in the AlphaFold2 model and 9 in the AlphaFold3 model, with differences largely due to variation in the orientation of side chains (**Supplementary Fig. 25, 26**). For example, the AVR-Pia^{R23} sidechain is predicted to form a hydrogen bond with the sidechain of OsHPP09-HMA^{D28} by AlphaFold3, and with the backbone of OsHPP09-HMA^{A27} by AlphaFold2. In the crystal structure, the positions of the AVR-Pia^{R23} and OsHPP09-HMA^{D28} sidechains enables AVR-Pia^{R23} to form hydrogen bonds with both OsHPP09-HMA^{D28} and OsHPP09-HMA^{A27} (**Supplementary Fig. 25, 26**). As AlphaFold does not predict the

positions of water molecules, the AlphaFold models lack the water-mediated interface contacts at the interface which can be identified in the crystal structure”.

Finally, we have added and a more detailed discussion of the solved structure and AF predictions (**lines 639-650**):

> “In recent years, advances in structural modelling have offered new insights and driven new lines of research in molecular plant-microbe interactions. Given reports that AlphaFold confidence metrics can be used to discriminate interacting protein pairs from those which do not interact [50,51,63], we were curious as to whether AlphaFold could identify the subset of H(I)PPs bound by AVR-Pia. Almost all AVR-Pia/HMA complexes, real and imagined, were modelled with high confidence scores to bind at a similar interface. The presence of an AVR-Pia/HMA structure in the PDB, used in the training of AlphaFold2 and AlphaFold3, may have influenced the results, highlighting a potential limitation of AlphaFold modelling. However, the intermolecular contacts were broadly well predicted, with a few discrepancies resulting from differences in the orientation of side chains. It should also be noted that the crystal structure of a protein (or protein complex) reflects a conformation of the protein that is amenable to crystallisation, which may not be identical to the structure of the protein in solution.”

If the interfaces are predicted correctly, it would not be a stretch to use the AF models of the other true interactors to probe further the interactions. Validating by mutagenesis that they all share the same interface (or not) would strength the scope and overall conclusions that could be drawn from the work.

To complement our new NMR titration data and interface mutagenesis results, we have added a detailed comparison of the resolved AVR-Pia/OsHPP09-HMA interface with the predicted AVR-Pia/OsHPP10/11 and OsHIPP21 interfaces (**Supplementary Fig. 36**) and have included the following paragraph in the discussion (**lines 718-742**):

> “Structure-guided mutagenesis of OsHPP09 identified that a single point mutation, E16A or E16R, could prevent AVR-Pia binding without perturbing the HMA domain fold. The OsHPP09^{E16} side chain forms an intermolecular hydrogen bond with the backbone of AVR-Pia and a salt bridge with the side chain of OsHPP09^{K19}, which also forms a hydrogen bond with the AVR-Pia backbone. This arrangement of hydrogen bonds likely stabilises the interaction between the two proteins and contributes to the high affinity binding of OsHPP09-HMA by AVR-Pia. By contrast, in the AlphaFold model of OsHPP10-HMA/AVR-Pia, the shorter aspartate side chain is too far from the AVR-Pia backbone to form an intermolecular hydrogen bond (**Supplementary Fig. 36**). This polymorphism is sufficient to significantly reduce the affinity of OsHPP10-HMA for AVR-Pia. However, mutating the aspartate to either alanine or arginine appears to further reduce the strength of interaction between OsHPP10 and AVR-Pia. The residue corresponding to OsHPP09^{E16} in OsHPP11 is threonine (T16); in the AlphaFold model of OsHPP11-HMA/AVR-Pia, the corresponding threonine side chain is too far from the effector to form an intermolecular hydrogen bond. As the side chains of both aspartate and threonine are hydrogen bond acceptors, it is possible that a water molecule could bridge the space between these residues and the AVR-Pia backbone. However, mutating the threonine to either alanine or arginine did not significantly affect the interaction between OsHPP11 and AVR-Pia. The glutamate residue is conserved in the HMA domain of OsHIPP21 (E15) and likely supports a similar network of hydrogen bonding to that observed in the OsHPP09-HMA/AVR-Pia crystal structure. This may explain why, despite lacking the D33 (L32 in OsHIPP21), Q20 (S19 in OsHIPP21) and D28 (K27 in OsHIPP21) residues which are conserved in the three HPPs and each form intermolecular hydrogen bonds in the OsHPP09-HMA/AVR-Pia structure, OsHIPP21-HMA is still bound by AVR-Pia. Consistent with this, mutation of OsHIPP21^{E15} to alanine or arginine reduced the strength of the interaction with AVR-Pia.”

The manuscript currently overplays engineering/susceptibility implications. Given AVR-Pia binds multiple host proteins, and since the functional relevance of these targets and AVR-Pia's contribution to virulence are not established here, claims about engineering host targets for durable resistance are speculative.

We agree with the reviewer and have significantly modified our discussion (**lines 701-708**).

> "It remains to be determined whether *OsHPP09*, *OsHPP10*, *OsHPP11,* and/or *OsHIPP21* are also *S* genes for rice blast disease, and whether deletion of any of these genes increases blast resistance. Notably, AVR-Pia contributes to virulence on rice [10]. Targeted mutagenesis of OsH(I)PPs to prevent AVR-Pia binding may reduce pathogen virulence without compromising the endogenous function of OsH(I)PPs. However, the extent of functional specificity and redundancy of both OsH(I)PPs and the effectors targeting them remains unknown, and may hamper efforts to improve resistance through modification of OsH(I)PPs."

MINOR ISSUES

Reviewer #1

1. The manuscript includes details concerning the purity of OsHPP09 prior to SEC, but analogous purification or protein purity information is not currently included for other proteins tested (OsHPP10/11/21) or mutants, although CD is performed on the mutants. I think these should be added as a supplementary figure, even just a gel of the purified proteins so readers can interpret the purity of what is going into ITC assays.

   We have included new supplementary figures (**Supplementary Fig. 10, 11, 28**) showing the final SEC elution trace and SDS-PAGE gel of a representative purification of each of the recombinant proteins produced for the experiments presented in this paper. Multiple purifications were carried out for most proteins; in each case the purity was confirmed by SDS-PAGE analysis.

2. Although I do not question the interaction or derived Kd values, ITC experiments are typically accompanied by key controls (e.g., AVR-Pia titrated into buffer alone, or buffer into protein) to demonstrate baseline heat of dilution. If not already performed, these should be included.

   We agree, and confirm that these controls were performed. The data have now been included as **Supplementary Fig. 15**.

3. AVR-Pia binding to the HMA domain via analytical SEC is included as a supplementary figure (Fig S7.), while the negative controls AVR-Pia-H3 and AVR1-CO39 are include in the main figures (Fig 3 and 4). This does not make much sense to me, I think Fig S7 should be included in main Fig 2.

   We have moved **Supplementary Fig. S7** to **main Fig. 1**.

4. The authors speculate that OsHPP09-HMA alone is a dimer (line 223) based on its elution profile on SEC. It subsequently appears as though that dimer must be disrupted to accommodate a 1:1 binding between the HMA and AVR-Pia. This would suggest that the HMA dimer and HMA-AVR-Pia interface are shared. Do the authors have any evidence that supports this? do mutations in OsHPP09 that impact interaction with AVR-Pia, also impact dimerization of the HMA domain.

We thank the reviewer for this question. We have added (**lines 244-255**) a comparison between the experimental structures of homodimers of OsPikp-1-HMA and OsRGA5-HMA, and the modelled OsHPP09-HMA homodimer (**Supplementary Fig. 12**), showing that OsHPP09-HMA predicted homodimer interface overlaps with the AVR-Pia/OsHPP09-HMA interface.

> "The elution volume of OsHPP09-HMA is consistent with dimerization, as has been reported for the integrated HMA domains of the NLR proteins OsPikp-1 and OsRGA5 in solution [12,45]. Modelling an OsHPP09-HMA dimer with AlphaFold3 predicts a similar dimerization interface formed from backbone hydrogen bonding between the β2 strands of the two protomers, which are arranged in opposing directions such that a continuous antiparallel β-sheet is formed from the eight β-strands of the two OsHPP09-HMA domains (**Supplementary Fig. 12**). This dimerization interface overlaps with the AVR-Pia binding surface predicted by AlphaFold. Following incubation with OsHPP09-HMA (1:1 effector:HMA ratio), the peak elution volume of AVR-Pia shifted to 12.7 ml. This is consistent with disruption of the OsHPP09-HMA dimer and formation of a 1:1 AVR-Pia/OsHPP09-HMA complex (**Fig. 1c**). The co-elution of the two proteins was supported by SDS-PAGE analysis of elution fractions (**Fig. 1c**)."

In addition, we have performed analytical size exclusion chromatography with the HMA domains of OsHPP09^{E16A} or OsHPP09^{E16R} (**Supplementary Fig. 32**). The elution volume was comparable to WT OsHPP09-HMA, leading us to conclude that these mutations do not impact dimerization (**lines 497-501**).

> "Given the overlap between the AVR-Pia-binding surface of OsHPP09-HMA and the putative dimerization interface, we used analytical size exclusion chromatography to investigate whether OsHPP09^{E16A} or OsHPP09^{E16R} dimerise in solution. Both mutated HMA domains eluted at a similar volume to WT OsHPP09-HMA, suggesting that the mutation does not interfere with dimerization (**Supplementary Fig. 32**)."

Finally, we have added discussion of this in **lines 743-754**:

> "Despite the predicted overlap of the dimerization interface with the effector binding surface, the E16A and E16R mutants do not appear to disrupt dimerization of OsHPP09-HMA. The AlphaFold3 model of the OsHPP09-HMA dimer predicts that the side chain of K19 forms an intermolecular salt bridge with the side chain of D33 of the opposing protomer. E16 is not directly involved in intermolecular bonds at the dimerization interface, though the side chain of E16 forms an intramolecular salt bridge with the side chain of neighbouring K19 which may stabilise the interface, and could also be involved in water-mediated contacts between protomers which are not revealed by AlphaFold modelling. While dimerization of HMA-IDs is not required for OsPik-1- or OsRGA5-mediated effector recognition and immune activation, it is unknown whether dimerization of OsHPP09-HMA holds biological significance. If dimerization is related to function, mutations that block effector binding without disrupting dimer formation could be of interest for engineering."

Reviewer #2:

1. This reviewer cannot locate Western blot results showing protein production of all fragments corresponding to Y2H of Suppl. Fig. 1.

   We thank the reviewer for highlighting this, these have been included as **Supplementary Fig. 2**.

2. Discrepancy of results of Y2H and split luciferase assay for OsHPP10 should be discussed.

We thank the reviewer for raising this point. The discrepancy between the Y2H and split-luciferase assays for OsHPP10 likely reflects differences in assay sensitivity and stringency rather than a true absence of interaction. Protein-protein interactions span a wide range of binding affinities, and different experimental systems operate within distinct detection windows.

We have added the following paragraph to the Discussion (**lines 626-638**):

> "We observed a discrepancy in OsHPP10-AVR-Pia interaction between Y2H and split-luciferase assays, with interaction detected in split-luciferase but not in Y2H. This likely reflects differences in assay sensitivity and stringency rather than a true absence of interaction [62]. Y2H requires stable interaction and transcriptional activation in the yeast nucleus and is generally more suited to detecting relatively strong interactions. In contrast, split-luciferase complementation is a highly sensitive in planta assay that benefits from proximity-induced signal amplification and can detect lower-affinity interactions. ITC showed that AVR-Pia binds OsHPP09-HMA with high affinity (K_D = 115 nM), whereas binding to OsHPP10-HMA is substantially weaker (K_D = 5.26 µM), with OsHPP11-HMA and OsHIPP21-HMA showing intermediate affinities. This affinity series is consistent with assay-dependent detection patterns. Stronger interactions (AVR-Pia with OsHPP09, OsHPP11, and OsHIPP21) are detected in Y2H, whereas weaker micromolar interactions fall below the detection range of Y2H but are detected in split-luciferase due to its higher sensitivity."

3. L460: "Consistent with this, the crystal structure of AVR-Pia/OsHPP09 revealed that AVR-Pia binds OsHPP09-HMA and OsRGA5-HMA through similar interfaces [13]." However, in L475, "This suggests that the RGA5-ID did not arise through duplication and integration of these AVR-Pia candidate targets, but instead from another H(I)PP". These interesting findings suggest that RGA5-HMA was derived from non-OsHPP09-type HMA, but binds and recognizes AVR-Pia using an interface which is similar to that used by AVR-Pia to bind OsHPP09. This indicates a convergent evolution of the interface structures. A bit more elaboration on this issue by including AVR-Pia/RGA-HMA interaction structure (or model) and its comparison with AVR-Pia/OsHPP09 structure would benefit the understanding of the readers.

   We thank the reviewer for this insightful comment, which highlights an interesting and somewhat unexpected aspect of our study. To better address this point, we have expanded the discussion with a more detailed and explicit paragraph (**lines 599-613**). We have also included in **Supplementary Fig. 24** a structural model of the AVR-Pia/OsRGA5-HMA interaction, together with a comparison to the AVR-Pia/OsHPP09 complex structure, to improve clarity for the reader.

   > "Interestingly, phylogenetic analysis of rice H(I)PP proteins (including NLRs with an HMA ID), based on the alignment of their HMA domains clusters OsRGA5-HMA within Clade A, which notably includes the H(I)PPs targeted by AVR-Pik and the HMA ID of OsPik-1 [27]. In contrast, the HMA domains of OsHPP09, OsHPP10, OsHPP11, and OsHIPP21 do not belong to this clade and are phylogenetically distant from that of OsRGA5. This indicates that the HMA ID of OsRGA5 did not arise through duplication and integration of these AVR-Pia candidate targets, but instead originated from another H(I)PP, likely belonging to Clade A. Future work aimed at reconstructing the evolutionary history of OsRGA5 will be necessary to further address this question. However, the fact that OsRGA5-HMA is able to bind AVR-Pia via an interface similar to that used by AVR-Pia to bind OsHPP09 (**Supplementary Fig. 24**) suggests a case of convergent evolution of the interface structures."

Reviewer #3:

1. Why was only LCI used to verify the interaction? There is a higher probability of false positives, so Co-IP should be added for further validation.

We thank the reviewer for this suggestion. We agree that Co-IP can provide valuable support for protein-protein interactions. However, in our system, we have consistently observed that FL HIPPs show a high level of non-specific association with MAX effectors in Co-IP experiments, which complicates interpretation and increases the risk of false-positive interactions. In contrast, the split-luciferase complementation assay provided more consistent results for assessing these interactions under our experimental conditions.

To address this, we focused on the HMA domains, which show improved specificity in Co-IPs. Using Co-IP with HMA domains, we observed strong binding between AVR-Pia and OsHPP09-HMA, as well as weaker interactions with OsHPP11-HMA and OsHIPP21-HMA (**Response Fig. 1**). In contrast, we did not detect an interaction between AVR-Pia and OsHPP10-HMA, consistent with our Y2H results and the lower binding affinity measured by ITC. The absence of detectable interaction in Co-IP is likely due to the weak, micromolar affinity of this interaction, which may not be retained under our wash conditions.

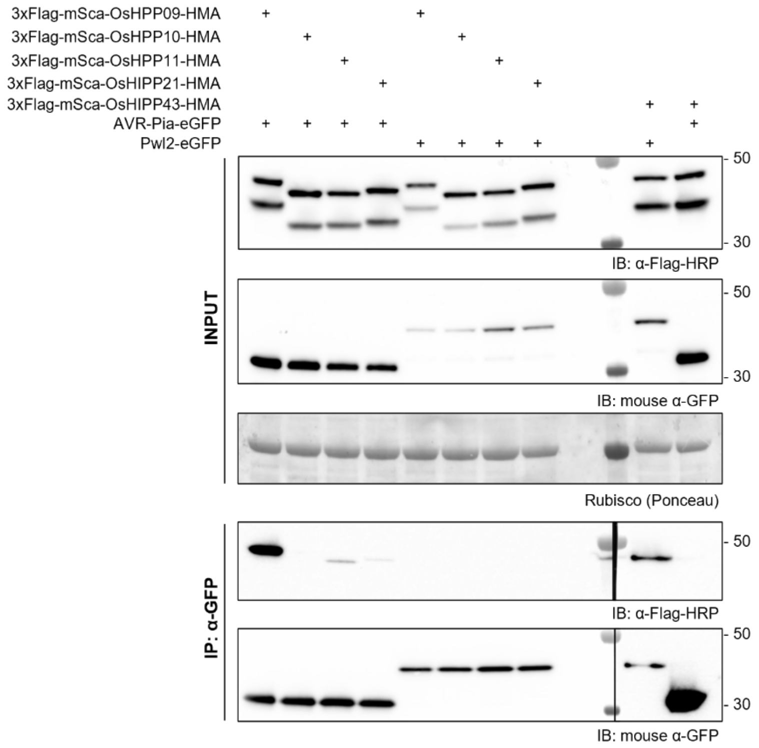

Response Fig. 1. Results of co-immunoprecipitation experiments with OsH(I)PP-HMA domains and AVR-Pia are consistent with results from Y2H assays. The indicated proteins were transiently co-expressed in *N. benthamiana*, and leaf tissue was harvested at 2 dpi. Total protein extracts were prepared using a low-stringency buffer, followed by immunoprecipitation with anti-GFP beads for 2 h. Immunoprecipitates were analysed by immunoblot using anti-GFP and anti-FLAG antibodies. Input samples (upper panel) represent total protein extracts prior to immunoprecipitation, while IP samples (lower panel) show the immunoprecipitated fractions. Equal protein loading was verified by Rubisco detection via Ponceau staining. For input samples, 10 µl per lane were loaded; for IP samples, 15 µl per lane were loaded, except for the positive control (3xFlag-mSca-OsHIPP43 / Pwl2-eGFP), for which 1.5 µl was loaded due to strong signal intensity. The IP membrane was cut prior to imaging to prevent signal saturation from the positive control and to allow detection of weaker signals. The experiment was performed three independent times with similar results; a representative replicate is shown here. mSca, mScarlet; eGFP, enhanced green fluorescent protein.

Together, these results are consistent with our existing data but do not add further support beyond what is already demonstrated, and therefore we have not included them in the manuscript.

2. Based on the interaction between MAX effectors and HIPPs, whether it could be inferred that HIPP09 is the virulence target of AVR-Pia? What is the relationship between HIPP09 and RGA5? Can in vivo genetic data be provided to explain this?

   We agree that this remains an open question, and have modified our discussion in response to this comment and the comments of reviewer 1 above (**lines 701-708**). Future work will address this through generation of rice knockouts for OsHPP09 (and OsHPP10, OsHPP11 and OsHIPP21). However, we hope the reviewer agrees that this is beyond the scope of the present study.

3. The author should explain from a structural perspective why AVR-Pia-H3 cannot interact with four OsHIPPs.

   We have added the following text to the discussion (**lines 585-591**):

   > "Both AVR-Pia^{F24} and AVR-Pia^{T46} are located at the HMA binding interface of AVR-Pia. AVR-Pia^{F24} contributes to a hydrophobic patch on AVR-Pia, and the replacement of phenylalanine by a polar serine residue presumably disrupts this hydrophobic patch. This substitution alone was sufficient to disrupt the interaction between AVR-Pia and OsRGA5-HMA [13]. Exchange of AVR-Pia^{T46} for the slightly larger asparagine may introduce steric hindrance, increasing the distance between the effector and HMA domain."

4. In Fig 4B, the interaction strengths of RGA5cterm and the other HIPPs with AVR1-CO39 are quite similar. Technique such as ITC or analytical gel filtration could be used to distinguish whether HIPPs actually bind to AVR1-CO39.

   We thank the reviewer for this suggestion. AVR1-CO39 is known to bind to RGA5cterm. However, in our split-luciferase assays, this interaction produced only a weak signal, marginally above background levels. This suggests that, under our experimental conditions, the split-luciferase system has limited sensitivity for detecting AVR1-CO39/RGA5cterm interactions. Importantly, we did not detect interactions between AVR1-CO39 and OsH(I)PPs in Y2H assays (**Fig. 4a**). In addition, analytical SEC did not reveal interaction between AVR1-CO39 and OsHPP09-HMA (**Fig. 4c**). These independent approaches do not support binding between AVR1-CO39 and H(I)PPs.

   Given the consistent lack of interaction across these independent assays, we consider that additional ITC or SEC experiments are unlikely to add substantially to the conclusions drawn from the existing data.

5. AVR1-CO39, AVR-Pik, and Pwl2 do not interact with HIPPs. This lack of interaction should be explained in the discussion from a structural perspective.

   AVR-Pik and Pwl2 interact with distinct groups of H(I)PPs to those bound by AVR-Pia. We have shown that AVR1-CO39 does not interact with the four H(I)PPs bound by AVR-Pia, yet it is unknown whether AVR1-CO39 binds other H(I)PPs. To complement our discussion on the differences between the interfaces between AVR-Pik/OsHIPP19-HMA and Pwl2/OsHIPP43-HMA (**lines 544-565, Supplementary Fig. 35**), we have modified and added the following text (**lines 531-543**):

   > "Like AVR-Pia, AVR1-CO39 is recognised by direct binding to the HMA ID of OsRGA5. Although the interaction surfaces between AVR-Pia/OsHPP09-HMA and AVR1-CO39/OsRGA5-HMA share similarities (**Supplementary Fig. 24**), as the β2 strand of the MAX effector interacts with the β2 strand of the HMA domain, the crystal structure of AVR-Pia/OsHPP09-HMA reveals that the side chains of several residues in the α1 helix of the HMA domain form intermolecular hydrogen bonds

with the effector. In contrast, the AVR1-CO39/OsRGA5-HMA interface is predominantly mediated by hydrogen bonding between the main chains."

6. From the structure of the HIPP09 and AVR-Pia complex in Fig 6, the interaction mode is similar to RGA5-HMA recognizing AVR1-CO39 and Pik-HMA recognizing AVR-Pia. All utilize the β2-α1 interface, suggesting that HMA domains recognize MAX effectors in a conserved manner. This differs from the distinct interface proposed by the authors. On the other hand, HMA domains appear to be multifaceted, recognizing non-MAX effectors via other interfaces, such as HMA120 recognizing AVR-Pita.

   We agree at a global level, the complex between AVR-Pia with OsHPP09-HMA resembles the complexes between AVR1-CO39/OsRGA5-HMA and AVR-Pia/OsPikp-1-HMA (**Supplementary Fig. 24a)**. In contrast, AVR-Pik binds the HMA-IDs of OsHIPP19 and Pik-1 at a different interface, mediated by hydrogen bonding between β3 of AVR-Pik and β4 of the HMA domain. While the Pwl2/HMA interface involves hydrogen bonding between β2 of the effector and β2 of the HMA domain, β3, β4, and the β3-β4 loop of the MAX fold also contribute to the HMA-binding interface. We therefore consider the AVR-Pia/OsHPP09-HMA, AVR-Pik/OsHIPP19-HMA and Pwl2/OsHIPP43-HMA interfaces to be distinct. We have clarified this prior to discussion of the different interfaces (**lines 544-545)**:

   > "Strikingly, the interactions between AVR-Pik, Pwl2, and AVR-Pia and the HMA domains of the H(I)PPs bound by these effectors involve distinct interfaces."

   We agree that it is highly interesting that HMA domains appear to be bound by multiple non-MAX effectors, such as AVR-Pita, from diverse pathogens and we have expanded on this idea in **lines 662-671** of the discussion.

   > "Further, the non-MAX *M. oryzae* effector AVR-Pita interacts with a HIPP HMA domain designated OsHMA120 [64]. Replacing the HMA-ID of OsRGA5 with OsHMA120 resulted in an engineered NLR protein conferring resistance to AVR-Pita. Together, this indicates that the manipulation of H(I)PPs is required for infection by diverse pests and pathogens and that H(I)PPs are targeted by structurally diverse pathogen proteins. Understanding how pathogen effectors bind H(I)PPs can offer opportunities to engineer novel resistance phenotypes [65]. However, little is known about the molecular mechanisms through which H(I)PPs affect plant resistance and/or susceptibility, and the precise functions of the effectors which target H(I)PPs."

7. E16 is critical for the interaction between HIPP09 and AVR-Pia. However, in HIPP11, this residue is replaced by Thr, which is neutral amino acid and has a shorter side chain. However, ITC results show that HIPP11 has a higher affinity than HIPP21 and HIPP10. Please provide a clear structural explanation for this.

   To address this comment and the comment of reviewer 1 (major revisions, above) we have added discussion of this to **lines 718-742**.

   > "Structure-guided mutagenesis of OsHPP09 identified that a single point mutation, E16A or E16R, could prevent AVR-Pia binding without perturbing the HMA domain fold. The OsHPP09^{E16} side chain forms an intermolecular hydrogen bond with the backbone of AVR-Pia and a salt bridge with the side chain of OsHPP09^{K19}, which also forms a hydrogen bond with the AVR-Pia backbone. This arrangement of hydrogen bonds likely stabilises the interaction between the two proteins and contributes to the high affinity binding of OsHPP09-HMA by AVR-Pia. By contrast, in the AlphaFold model of OsHPP10-HMA/AVR-Pia, the shorter aspartate side chain is too far from the AVR-Pia backbone to form an intermolecular hydrogen bond (**Supplementary Fig. 36**). This polymorphism is sufficient to significantly reduce the affinity of OsHPP10-HMA for AVR-Pia. However, mutating the aspartate to either alanine or arginine appears to further reduce the strength of interaction between OsHPP10 and AVR-Pia.

The residue corresponding to OsHPP09^{E16} in OsHPP11 is threonine (T16); in the AlphaFold model of OsHPP11-HMA/AVR-Pia, the corresponding threonine side chain is too far from the effector to form an intermolecular hydrogen bond. As the side chains of both aspartate and threonine are hydrogen bond acceptors, it is possible that a water molecule could bridge the space between these residues and the AVR-Pia backbone. However, mutating the threonine to either alanine or arginine did not significantly affect the interaction between OsHPP11 and AVR-Pia. The glutamate residue is conserved in the HMA domain of OsHIPP21 (E15) and likely supports a similar network of hydrogen bonding to that observed in the OsHPP09-HMA/AVR-Pia crystal structure. This may explain why, despite lacking the D33 (L32 in OsHIPP21), Q20 (S19 in OsHIPP21) and D28 (K27 in OsHIPP21) residues which are conserved in the three HPPs and each form intermolecular hydrogen bonds in the OsHPP09-HMA/AVR-Pia structure, OsHIPP21-HMA is still bound by AVR-Pia. Consistent with this, mutation of OsHIPP21^{E15} to alanine or arginine reduced the strength of the interaction with AVR-Pia."

8. OsHPP10 did not interact with AVR-Pia in Y2H (Fig. 1a) but showed strong interaction in split luciferase assays (Fig. 1b). Please discuss this discrepancy in more detail.

We thank the reviewer for this comment, which was also raised by Reviewer 2. Please see our response to Reviewer 2, Comment 2. Briefly, we attribute this discrepancy to differences in assay sensitivity and detection thresholds. ITC measurements revealed that AVR-Pia binds OsHPP10-HMA with lower affinity compared to the other OsH(I)PP-HMA domains, placing the interaction below the detection range of Y2H assays while remaining detectable in the more sensitive split-luciferase system, which relies on proximity-induced reconstitution of luciferase activity and can therefore capture lower-affinity interactions. We have added a paragraph to the discussion to clarify this point (**lines 626-638**):

"We observed a discrepancy in OsHPP10-AVR-Pia interaction between Y2H and split-luciferase assays, with interaction detected in split-luciferase but not in Y2H. This likely reflects differences in assay sensitivity and stringency rather than a true absence of interaction [62]. Y2H requires stable interaction and transcriptional activation in the yeast nucleus and is generally more suited to detecting relatively strong interactions. In contrast, split-luciferase complementation is a highly sensitive in planta assay that benefits from proximity-induced signal amplification and can detect lower-affinity interactions. ITC showed that AVR-Pia binds OsHPP09-HMA with high affinity (K_D = 115 nM), whereas binding to OsHPP10-HMA is substantially weaker (K_D = 5.26 µM), with OsHPP11-HMA and OsHIPP21-HMA showing intermediate affinities. This affinity series is consistent with assay-dependent detection patterns. Stronger interactions (AVR-Pia with OsHPP09, OsHPP11, and OsHIPP21) are detected in Y2H, whereas weaker micromolar interactions fall below the detection range of Y2H but are detected in split-luciferase due to its higher sensitivity."

9. In the Discussion, please summarise more clearly that while prenylation does not explain the lack of HMA only interaction, the full length context (possibly including the C terminal region) appears necessary for stable binding. This could be linked to the observation that other MAX effectors (e.g., Pwl2) use extensions beyond the core fold for HMA binding.

We thank the reviewer for this comment. However, we are not entirely certain that we have fully understood its intent, and we hope that the response below adequately addresses the point raised.

Among the AVR-Pia interactors we analysed in yeast two-hybrid assays, OsHIPP21 is the only case in which the HMA domain alone does not appear to be sufficient, and where the full-length protein is required for detectable binding. This observation is restricted to yeast two-hybrid assays as, in contrast, our in vitro analyses clearly indicate that AVR-Pia can

bind to the isolated HMA domain of OsHIPP21, suggesting that the C-terminal region, including the prenylation motif, is not essential for this interaction.

In addition, as noted in our response to the first comment of reviewer #3, our co-immunoprecipitation experiments show a weak but detectable association between AVR-Pia and the HMA domain of OsHIPP21, further reinforcing the conclusion that the HMA domain alone is sufficient for binding in a cellular context.

That said, we cannot exclude the possibility that the C-terminal region contributes to enhancing or stabilising the interaction. In the absence of structural or biochemical evidence demonstrating a direct role for this region in binding to AVR-Pia or in increasing interaction stability, we consider it premature to draw firm conclusions regarding its functional necessity.

Finally, the proposed link with MAX effectors that possess extensions beyond the canonical MAX fold does not appear directly applicable in this case, as AVR-Pia does not contain such an extension.

10. The I35K mutation abolished interaction, but CD spectroscopy shows it disrupts the HMA fold (Fig. 7c). It would be helpful to explicitly state in the Results (around lines 382–383) that I35K is unsuitable as an editing target because the loss of binding is due to misfolding, in contrast to E16A/E16R which retain fold but lose binding. This reinforces the value of the E16 mutations for potential engineering of resistance.

    We thank the reviewer for this comment. We added the following sentence to the results section (**lines 474-476**):

    > “Disruption of AVR-Pia binding to the mutated HMA domains may be caused by a specific loss of interaction at the interface or by broader destabilisation of the HMA domain fold” to the results section.”
