# Supplementary material for: Two folds, many faces: The Magnaporthe oryzae MAX effector AVR-Pia targets novel rice HMA domain-containing proteins
Source: PLoS Pathog. 2026 Jul 13;22(7):e1014382. doi: 10.1371/journal.ppat.1014382 (PMC13395435; doi:10.1371/journal.ppat.1014382)
Supplement: Peer review history

# Author comment: Author response to Decision Letter 0

**DOI:** 10.1371/journal.ppat.1014382.r001

**Transfer Alert**

This paper was transferred from another journal. As a result, its full editorial history (including decision letters, peer reviews and author responses) may not be present.

1 Dec 2025

# Peer review: Decision Letter 0

**DOI:** 10.1371/journal.ppat.1014382.r002

**Authors:** Shou-Wei Ding, Ksenia Krasileva

3 Mar 2026

-->PPATHOGENS-D-25-02941

Two folds, many faces: the *Magnaporthe oryzae*  MAX effector AVR-Pia targets novel rice HMA domain-containing proteins

PLOS Pathogens

Dear Dr. Cesari,

Thank you for submitting your manuscript to PLOS Pathogens. After careful consideration, we feel that it has merit but does not fully meet PLOS Pathogens's publication criteria as it currently stands. Therefore, we invite you to submit a revised version of the manuscript that addresses the points raised during the review process.

Please submit your revised manuscript by May 02 2026 11:59PM. If you will need more time than this to complete your revisions, please reply to this message or contact the journal office at plospathogens@plos.org. When you're ready to submit your revision, log on to https://www.editorialmanager.com/ppathogens/ and select the 'Submissions Needing Revision' folder to locate your manuscript file.

Please include the following items when submitting your revised manuscript:

* A letter that responds to each point raised by the editor and reviewer(s). You should upload this letter as a separate file labeled 'Response to Reviewers'. This file does not need to include responses to any formatting updates and technical items listed in the 'Journal Requirements' section below.

* A marked-up copy of your manuscript that highlights changes made to the original version. You should upload this as a separate file labeled 'Revised Manuscript with Track Changes'.

* An unmarked version of your revised paper without tracked changes. You should upload this as a separate file labeled 'Manuscript'.

If you would like to make changes to your financial disclosure, competing interests statement, or data availability statement, please make these updates within the submission form at the time of resubmission. Guidelines for resubmitting your figure files are available below the reviewer comments at the end of this letter.

We look forward to receiving your revised manuscript.

Kind regards,

Ksenia Krasileva

Guest Editor

PLOS Pathogens

Shou-Wei Ding

Section Editor

PLOS Pathogens

Sumita Bhaduri-McIntosh

Editor-in-Chief

PLOS Pathogens

orcid.org/0000-0003-2946-9497

Michael Malim

Editor-in-Chief

PLOS Pathogens

orcid.org/0000-0002-7699-2064

**Additional Editor Comments :**

As you can see from the reviewers’ comments, all of the reviewers recognize the high technical quality of the work an have only minor suggestions for improving the presented data. However, they also raise a major concern that the scope of the study could be broadened and strengthened for publication in PLOS Pathogens. Please consider all of the reviewers’ suggestions, especially those that would increase the work’s relevance to a broader audience and enhance the general applicability of the findings, such as comparisons between the solved structure and AF prediction, examining the shared interaction interface through computational analyses, and selected genetic testing for its validity (negative or positive).

**Journal Requirements:**

1) We ask that a manuscript source file is provided at Revision. Please upload your manuscript file as a .doc, .docx, .rtf or .tex. If you are providing a .tex file, please upload it under the item type u2018LaTeX Source Fileu2019 and leave your .pdf version as the item type u2018Manuscriptu2019.

2) We noticed that you used the phrase 'unpublished ' in the manuscript. We do not allow these references, as the PLOS data access policy requires that all data be either published with the manuscript or made available in a publicly accessible database. Please amend the supplementary material to include the referenced data or remove the references.

3) We do not publish any copyright or trademark symbols that usually accompany proprietary names, eg ©,  ®, or TM  (e.g. next to drug or reagent names). Therefore please remove all instances of trademark/copyright symbols throughout the text, including:

- ® on pages: 18, 19, 20, 21, and 22

- TM on pages: 18, 21, 22, and 23.

4) Please upload all main figures as separate Figure files in .tif or .eps format. For more information about how to convert and format your figure files please see our guidelines:

https://journals.plos.org/plospathogens/s/figures

5) We have noticed that you have uploaded Supporting Information files, but you have not included a list of legends. Please add a full list of legends for your Supporting Information files after the references list.

6) Please ensure that the funders and grant numbers match between the Financial Disclosure field and the Funding Information tab in your submission form. Note that the funders must be provided in the same order in both places as well. Currently, "French Infrastructure for Integrated Structural Biology" is missing from the Financial Disclosure field.

Note: If the reviewer comments include a recommendation to cite specific previously published works, please review and evaluate these publications to determine whether they are relevant and should be cited. There is no requirement to cite these works unless the editor has indicated otherwise.

**Reviewers' Comments:**

Reviewer's Responses to Questions

**Part I - Summary**

Please use this section to discuss strengths/weaknesses of study, novelty/significance, general execution and scholarship.

Reviewer #1: Maidment and colleagues present a well-structured and experimentally robust study that identifies host targets of the Magnaporthe oryzae effector AVR-Pia. The authors use complementary approaches, including targeted (HMA containing host gene library) and non-targeted yeast two-hybrid (Y2H) screens to initially identify interactors. This was supported by in planta split-luciferase assays in N. benthamiana and biochemical validation (ITC). Additional controls such as the MAX58 protein, a structural homolog of AVR-Pia, the AVR-Pia-H3 mutant and AVR1-CO39 show the specificity of the interaction between AVR-Pia and OsHPP09, 10, 11 and the more sequence diverse 21.

Much of the study focused on OsHPP09, which had the strongest binding affinity to AVR-Pia. Importantly the authors could resolve a complex of the two proteins via SEC and were able to co-crystalize the complex and solve the structure to high resolution using X-ray crystallography. Validation of the interface observed in the structure was supported using NMR titration experiments and rationale mutagenesis.

The major outcomes/novelty of the work is the observation that AVR-Pia targets are different to host HMA domains to other MAX effectors. Another conclusion that can be drawn from the study is that AVR-Pia interacts with OsHPP09 in a similar binding mode to the interaction with the integrated HMA domain of RGA-5, albeit with different binding affinities.

A major strength of this manuscript is the rigor and breadth of validation. Ultimately the claims made by the authors within the results are very well supported by the data.

However, while the work is technically strong, I feel the conceptual advance is more incremental than transformative. A large body of literature underpins our current understanding regarding MAX effector and HMA interactions, including host targets and integrated decoy HMA domains. While the key advances here (identification of AVR-Pia host targets; conservation in binding mode between HMA host target and NLR decoy) are interesting the broader biological implications are not resolved.

It remains unclear if any of these interactions are important for the virulence function of AVR-Pia and while this is acknowledged within the manuscript, the suggestion that the work itself lays a foundation for engineering resistance by manipulating a susceptibility target is a massive jump, particularly given there is no evidence to even suggest these interactions have any real impact on disease.

The work itself does not establish if the interactions between AVR-Pia and the HMA interactors are conserved, which I believe could have been addressed in the paper. Considering the number of AVR-Pia interactors and the potential for all or any of these interactions to contribute to disease one could easily mount a counter argument that altering the AVR-Pia targets could be an inefficient and poor strategy to improve disease resistance in this pathosystem.

I don’t wish to be overly negative, I enjoyed the paper, the quality of the work is very high but I do question the novelty of the findings and implications of the work across the host-pathogen field.

Clearly others may disagree with me concerning my conclusion about novelty/significance. I would like to highlight some changes that I believe could help extended the conclusions made (detailed below), although major additions to this work would be required to provide any biological or functional relevance to the findings.

Reviewer #2: Review of the manuscript entitled “Two folds, many faces: the Magnaporthe oryzae MAX effector AVR-Pia targets novel rice HMA domain-containing proteins” by Maidment et al. submitted to PLoS Pathogens

This paper describes molecular interactions between rice blast fungal effector AVR-Pia and rice proteins belonging to a family of the heavy-metal associated (HMA) domain-containing proteins. Previous studies showed that blast fungal effector AVR-Pia, a member of MAX-fold effectors, is recognized by RGA5 sensor NLR via its binding to HMA-integrated domain of RGA5. However, host target proteins of AVR-Pia have not been identified. In this paper, the authors show four rice HMA-containing proteins, OsHPP09, OsHPP10, OsHPP11 and OsHIPP21, bind AVR-Pia. Crystal structure of AVR-Pia/OsHPP09 protein complex was solved. Interestingly, the interaction surface of AVR-Pia/OsHIPP09-HMA is different from these of AVR-Pik/Pik1-HMA and Pwl2/OsHIPP43-HMA, indicating that different surfaces are used for recognition interactions and possible virulence interactions.

Interactions between AVR-Pia and rice HMA proteins were studied by Y2H (Fig. 1) using the HMA-domain fragments and full-length proteins, revealing five proteins, OsHPP09, OsHPP41, OsHPP11, OsHPP14 and OsHIPP39, interact with AVR-Pia. In planta interactions between AVR-Pia and HMA proteins were tested by split luciferase complementation assay using Nicotiana benthamiana transient expression (Fig. 1b), revealing interactions of AVR-Pia with OsHPP09, OsHPP10, OsHPP11 and OsHIPP21 (Fig. 1b). Affinity of interactions between AVR-Pia and OsHPP09, OsHPP10, OsHPP11 and OsHIPP21 was tested by ITC experiments (Fig. 2), confirming the strong interaction of AVR-Pia to OsHPP09. Alphafold structure prediction was not able to differentiate AVR-Pia-binding HMA proteins from non-binding proteins (Suppl. Fig. 10,11). A variant of AVR-Pia, AVR-Pia-H3, did not bind OsHPP09, OsHPP10, OsHPP11 and OsHIPP21 (Fig. 3). AVR1-CO39, an effector recognized by RGA5 via its binding to HMA-ID, showed no interaction with OsHPP09, OsHPP10, OsHPP11 and OsHIPP21 (Fig. 4). The two previously characterized Magnaporthe effectors, AVR-Pik and Pwl2 did not interact with OsHPP09, OsHPP10, OsHPP11 and OsHIPP21 (Fig. 5). Crystal structure of AVR-Pia/ OsHPP09 -HMA domain complex was obtained (Fig. 6), revealing that the interaction is structurally similar to AVR-Pia/OsPikp-1-HMA and AVR1-CO39/OsRGA5-HMA interactions (Suppl. Fig. 16). Further analysis was done with NMR analysis (Suppl. Fig.17,18,19). Based on the structure information, the authors replaced key amino acid residues of OsHPP09, resulting in the reduction of interaction as validated by the experiments (Fig. 7).

The finding of four HMA proteins OsHPP09, OsHPP10, OsHPP11 and OsHIPP21 as AVR-Pia effector interactors expands our understanding of Magnaporthe effector-rice host protein interactions. Structure study of AVR-Pia/OsHPP09 points to the diversity of interaction surfaces between the effectors and the host proteins. The data are solid. The manuscript is well written, but is overall descriptive without in-depth analysis of the possible function of OsHIPP09 and how AVR-Pia binding affects the function of OsHIPP9. However, it may be an important contribution as a solid record of interactions between pathogen effectors and host proteins.

Reviewer #3: This study identifies novel host targets of the Magnaporthe oryzae MAX effector AVR-Pia – four rice HMA domain‑containing proteins (OsHPP09, OsHPP10, OsHPP11 and OsHIPP21). Using a combination of Y2H screens, in planta validation (split‑luciferase), quantitative binding assays (ITC), X‑ray crystallography and NMR, the authors demonstrate that AVR-Pia binds these targets with distinct affinities. The crystal complex structure of AVR-Pia/OsHPP09 reveals the molecular basis of high‑affinity binding and, through comparison with other MAX effector/HMA complexes. Structure‑guided mutagenesis further identifies a single residue in OsHPP09 that is critical for effector binding without compromising protein folding. However, some concerns remain that need to be addressed to strengthen the manuscript.

**********

**Part II – Major Issues: Key Experiments Required for Acceptance**

Please use this section to detail the key new experiments or modifications of existing experiments that should be absolutely required to validate study conclusions.

Generally, there should be no more than 3 such required experiments or major modifications for a "Major Revision" recommendation. If more than 3 experiments are necessary to validate the study conclusions, then you are encouraged to recommend "Reject".

Reviewer #1: A central opportunity for this manuscript is to clarify whether AVR-Pia engages diverse HMA domains (host targets and/or integrated decoys) through a conserved binding mode. The current work convincingly solves one interaction (e.g., AVR-Pia–OsHPP09-HMA) and could compare that with the interaction with the integrated HMA of RGA5. Despite this it stops short of establishing whether the identified interface is representative across interactors like 10, 11 and 21. Is E16 conserved within any of the other OsHPP proteins, how would mutation of this, or the equivalent residue, impact these interactions?

The authors make the observation that AF2 and AF3 are unable to distinguish the true/false interactors, an important result. Despite this it appears to me based on the figures provided that AF does predict the interface itself correctly. In fact, almost all the models predict the same binding mode. I don’t think this is mentioned in the current manuscript, I do believe further comparisons between the solved structure and AF prediction should be included. If the interfaces are predicted correctly, it would not be a stretch to use the AF models of the other true interactors to probe further the interactions. Validating by mutagenesis that they all share the same interface (or not) would strength the scope and overall conclusions that could be drawn from the work.

The manuscript currently overplays engineering/susceptibility implications. Given AVR-Pia binds multiple host proteins, and since the functional relevance of these targets and AVR-Pia’s contribution to virulence are not established here, claims about engineering host targets for durable resistance are speculative.

Reviewer #2: (No Response)

Reviewer #3: none

**********

**Part III – Minor Issues: Editorial and Data Presentation Modifications**

Please use this section for editorial suggestions as well as relatively minor modifications of existing data that would enhance clarity.

Reviewer #1: The manuscript includes details concerning the purity of OsHPP09 prior to SEC, but analogous purification or protein purity information is not currently included for other proteins tested (OsHPP10/11/21) or mutants, although CD is performed on the mutants. I think these should be added as a supplementary figure, even just a gel of the purified proteins so readers can interpret the purity of what is going into ITC assays.

Although I do not question the interaction or derived Kd values, ITC experiments are typically accompanied by key controls (e.g., AVR-Pia titrated into buffer alone, or buffer into protein) to demonstrate baseline heat of dilution. If not already performed, these should be included.

AVR-Pia binding to the HMA domain via analytical SEC is included as a supplementary figure (Fig S7.), while the negative controls AVR-Pia-H3 and AVR1-CO39 are include in the main figures (Fig 3 and 4). This does not make much sense to me, I think Fig S7 should be included in main Fig 2.

The authors speculate that OsHPP09-HMA alone is a dimer (line 223) based on its elution profile on SEC. It subsequently appears as though that dimer must be disrupted to accommodate a 1:1 binding between the HMA and AVR-Pia. This would suggest that the HMA dimer and HMA-AVR-Pia interface are shared. Do the authors have any evidence that supports this? do mutations in OsHPP09 that impact interaction with AVR-Pia, also impact dimerization of the HMA domain.

Reviewer #2: 1. This reviewer cannot locate Western blot results showing protein production of all fragments corresponding to Y2H of Suppl. Fig. 1.

2. Discrepancy of results of Y2H and split luciferase assay for OsHPP10 should be discussed.

3. L460: “Consistent with this, the crystal structure of AVR-Pia/OsHPP09 revealed that AVR-Pia binds OsHPP09-HMA and OsRGA5-HMA through similar interfaces[13].” However, in L475, “This suggests that the RGA5-ID did not arise through duplication and integration of these AVR-Pia candidate targets, but instead from another H(I)PP. These interesting findings suggest that RGA5-HMA was derived from non-OsHPP09-type HMA, but binds and recognizes AVR-Pia using an interface which is similar to that used by AVR-Pia to bind OsHPP09. This indicates a convergent evolution of the interface structures. A bit more elaboration on this issue by including AVR-Pia/RGA-HMA interaction structure (or model) and its comparison with AVR-Pia/OsHPP09 structure would benefit the understanding of the readers.

Reviewer #3: 1.Why was only LCI used to verify the interaction? There is a higher probability of false positives, so Co-IP should be added for further validation.

2.Based on the interaction between MAX effectors and HIPPs, whether it could be inferred that HIPP09 is the virulence target of AVR-Pia? What is the relationship between HIPP09 and RGA5? Can in vivo genetic data be provided to explain this?

3.The author should explain from a structural perspective why AVR-Pia-H3 cannot interact with four OsHIPPs.

4.In Fig 4B, the interaction strengths of RGA5cterm and the other HIPPs with AVR1-CO39 are quite similar. Techniquesuch as ITC or analytical gel filtration could be used to distinguish whether HIPPs actually bind to AVR1-CO39.

5.AVR1-CO39, AVR-Pik, and Pwl2 do not interact with HIPPs. This lack of interaction should be explained in the discussion from a structural perspective.

6.From the structure of the HIPP09 and AVR-Pia complex in Fig 6, the interaction mode is similar to RGA5-HMA recognizing AVR1-CO39 and Pik-HMA recognizing AVR-Pia. All utilize the β2-α1 interface, suggesting that HMA domains recognize MAX effectors in a conserved manner. This differs from the distinct interface proposed by the authors. On the other hand, HMA domains appear to be multifaceted, recognizing non-MAX effectors via other interfaces, such as HMA120 recognizing AVR-Pita.

7.E16 is critical for the interaction between HIPP09 and AVR-Pia. However, in HIPP11, this residue is replaced by Thr, which is neutral amino acid and has a shorter side chain. However, ITC results show that HIPP11 has a higher affinity than HIPP21 and HIPP10. Please provide a clear structural explanation for this.

8.OsHPP10 did not interact with AVR-Pia in Y2H (Fig. 1a) but showed strong interaction in split‑luciferase assays (Fig. 1b). Please discuss this discrepancy in more detail.

9.In the Discussion, please summarise more clearly that while prenylation does not explain the lack of HMA‑only interaction, the full‑length context (possibly including the C‑terminal region) appears necessary for stable binding. This could be linked to the observation that other MAX effectors (e.g., Pwl2) use extensions beyond the core fold for HMA binding.

10.The I35K mutation abolished interaction, but CD spectroscopy shows it disrupts the HMA fold (Fig. 7c). It would be helpful to explicitly state in the Results (around lines 382–383) that I35K is unsuitable as an editing target because the loss of binding is due to misfolding, in contrast to E16A/E16R which retain fold but lose binding. This reinforces the value of the E16 mutations for potential engineering of resistance.

**********

PLOS authors have the option to publish the peer review history of their article (what does this mean?). If published, this will include your full peer review and any attached files.

If you choose “no”, your identity will remain anonymous but your review may still be made public.

**Do you want your identity to be public for this peer review?**  For information about this choice, including consent withdrawal, please see our Privacy Policy.

Reviewer #1: No

Reviewer #2: No

Reviewer #3: No

[NOTE: If reviewer comments were submitted as an attachment file, they will be attached to this email and accessible via the submission site. Please log into your account, locate the manuscript record, and check for the action link "View Attachments". If this link does not appear, there are no attachment files.]

**Figure resubmission:**

-->While revising your submission, we strongly recommend that you use PLOS’s NAAS tool (https://ngplosjournals.pagemajik.ai/artanalysis) to test your figure files. NAAS can convert your figure files to the TIFF file type and meet basic requirements (such as print size, resolution), or provide you with a report on issues that do not meet our requirements and that NAAS cannot fix.

After uploading your figures to PLOS’s NAAS tool - https://ngplosjournals.pagemajik.ai/artanalysis, NAAS will process the files provided and display the results in the "Uploaded Files" section of the page as the processing is complete. If the uploaded figures meet our requirements (or NAAS is able to fix the files to meet our requirements), the figure will be marked as "fixed" above. If NAAS is unable to fix the files, a red "failed" label will appear above. When NAAS has confirmed that the figure files meet our requirements, please download the file via the download option, and include these NAAS processed figure files when submitting your revised manuscript.

**Reproducibility:**

To enhance the reproducibility of your results, we recommend that authors of applicable studies deposit laboratory protocols in protocols.io, where a protocol can be assigned its own identifier (DOI) such that it can be cited independently in the future. Additionally, PLOS ONE offers an option to publish peer-reviewed clinical study protocols. Read more information on sharing protocols at https://plos.org/protocols?utm_medium=editorial-email&utm_source=authorletters&utm_campaign=protocols

# Author comment: Author response to Decision Letter 1

**DOI:** 10.1371/journal.ppat.1014382.r003

6 May 2026

Attachment Submitted filename: 2026-04-30_response-to-reviewers_v7.pdf

# Peer review: Decision Letter 1

**DOI:** 10.1371/journal.ppat.1014382.r004

**Authors:** Shou-Wei Ding, Ksenia Krasileva

15 Jun 2026

Dear Dr Cesari,

Congratulations!

We are happy to inform you that your manuscript 'Two folds, many faces: the *Magnaporthe oryzae*  MAX effector AVR-Pia targets novel rice HMA domain-containing proteins' has been provisionally accepted for publication in PLOS Pathogens.

Before your manuscript can be formally accepted you will need to complete some formatting changes, which you will receive in a follow up email. A member of our team will be in touch with a set of requests.

Please note that your manuscript will not be scheduled for publication until you have made the required changes, so a swift response is appreciated.

IMPORTANT: The editorial review process is now complete. PLOS will only permit corrections to spelling, formatting or significant scientific errors from this point onwards. Requests for major changes, or any which affect the scientific understanding of your work, will cause delays to the publication date of your manuscript.

Should you, your institution's press office or the journal office choose to press release your paper, you will automatically be opted out of early publication. We ask that you notify us now if you or your institution is planning to press release the article. All press must be co-ordinated with PLOS.

Thank you again for supporting Open Access publishing; we are looking forward to publishing your work in PLOS Pathogens.

Best regards,

Ksenia Krasileva

Guest Editor

PLOS Pathogens

Shou-Wei Ding

Section Editor

PLOS Pathogens

Sumita Bhaduri-McIntosh

Editor-in-Chief

PLOS Pathogens

orcid.org/0000-0003-2946-9497

Michael Malim

Editor-in-Chief

PLOS Pathogens

orcid.org/0000-0002-7699-2064

***********************************************************

Reviewer Comments (if any, and for reference):

Reviewer's Responses to Questions

**Part I - Summary**

Please use this section to discuss strengths/weaknesses of study, novelty/significance, general execution and scholarship.

Reviewer #1: The authors have address the comments raised in my initial review, with the additional of a significant amount of new data that generally support the manuscripts findings. They have also changed some of the language concerning the advances in engineer that better reflect the outcomes of the work. Collectively, the authors have taken on the comments of the reviewers and present an improved revised manuscript that reflects that.

Reviewer #2: In this revised version, the authors adequately addressed all the comments of this reviewer.

Reviewer #3: I am content with this version.

**********

**Part II – Major Issues: Key Experiments Required for Acceptance**

Please use this section to detail the key new experiments or modifications of existing experiments that should be absolutely required to validate study conclusions.

Generally, there should be no more than 3 such required experiments or major modifications for a "Major Revision" recommendation. If more than 3 experiments are necessary to validate the study conclusions, then you are encouraged to recommend "Reject".

Reviewer #1: (No Response)

Reviewer #2: In this revised version, the authors adequately addressed all the comments of this reviewer.

Reviewer #3: None

**********

**Part III – Minor Issues: Editorial and Data Presentation Modifications**

Please use this section for editorial suggestions as well as relatively minor modifications of existing data that would enhance clarity.

Reviewer #1: (No Response)

Reviewer #2: No minor issues remain.

Reviewer #3: None

**********

PLOS authors have the option to publish the peer review history of their article (what does this mean?). If published, this will include your full peer review and any attached files.

If you choose “no”, your identity will remain anonymous but your review may still be made public.

**Do you want your identity to be public for this peer review?**  For information about this choice, including consent withdrawal, please see our Privacy Policy.

Reviewer #1: No

Reviewer #2: No

Reviewer #3: No

# Editor report: Acceptance letter

**DOI:** 10.1371/journal.ppat.1014382.r005

**Authors:** Shou-Wei Ding, Ksenia Krasileva

Dear Dr Cesari,

We are delighted to inform you that your manuscript, "

Two folds, many faces: the *Magnaporthe oryzae*  MAX effector AVR-Pia targets novel rice HMA domain-containing proteins," has been formally accepted for publication in PLOS Pathogens.

We have now passed your article onto the PLOS Production Department who will complete the rest of the pre-publication process. All authors will receive a confirmation email upon publication.

The corresponding author will soon be receiving a typeset proof for review, to ensure errors have not been introduced during production. Please review the PDF proof of your manuscript carefully, as this is the last chance to correct any scientific or type-setting errors. Please note that major changes, or those which affect the scientific understanding of the work, will likely cause delays to the publication date of your manuscript. Note: Proofs for Front Matter articles (Pearls, Reviews, Opinions, etc...) are generated on a different schedule and may not be made available as quickly.

Soon after your final files are uploaded, the early version of your manuscript, if you opted to have an early version of your article, will be published online. The date of the early version will be your article's publication date. The final article will be published to the same URL, and all versions of the paper will be accessible to readers.

For Research Articles, you will receive an invoice from PLOS for your publication fee after your manuscript has reached the completed accept phase. If you receive an email requesting payment before acceptance or for any other service, this may be a phishing scheme. Learn how to identify phishing emails and protect your accounts at https://explore.plos.org/phishing.

Thank you again for supporting open-access publishing; we are looking forward to publishing your work in PLOS Pathogens.

Best regards,

Sumita Bhaduri-McIntosh

Editor-in-Chief

PLOS Pathogens

orcid.org/0000-0003-2946-9497

Michael Malim

Editor-in-Chief

PLOS Pathogens

orcid.org/0000-0002-7699-2064
